# Enrichment and Quantitation of Dipeptidyl Peptidase IV Inhibitory Peptides in Quinoa upon Systematic Malting

**DOI:** 10.1021/acs.jafc.4c00570

**Published:** 2024-05-11

**Authors:** Tabea D. U. Kröber, Magdalena Holzer, Roland Kerpes, Verena K. Mittermeier-Kleßinger, Corinna Dawid, Thomas Becker

**Affiliations:** † Chair of Brewing and Beverage Technology, School of Life Sciences Weihenstephan, Technical University of Munich, Weihenstephaner Steig 20, 85354 Freising, Germany; ‡ Chair of Food Chemistry and Molecular Sensory Science, School of Life Sciences Weihenstephan, Technical University of Munich, Lise-Meitner-Strasse 34, 85354 Freising, Germany; § Professorship for Functional Phytometabolomics, School of Life Sciences Weihenstephan, Technical University of Munich, Lise-Meitner-Strasse 34, 85354 Freising, Germany

**Keywords:** type 2 diabetes, dipeptidyl peptidase IV inhibitor, malting, quinoa, simulated gastrointestinal digest, bioactive peptides

## Abstract

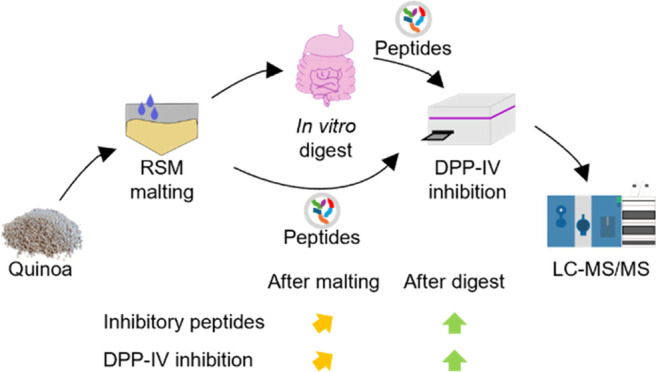

Food-derived peptides with an inhibitory effect on dipeptidyl peptidase IV (DPP-IV) can be used as an additive treatment for type 2 diabetes. The inhibitory potential of food depends on technological protein hydrolysis and gastrointestinal digestion, as the peptides only act after intestinal resorption. The effect of malting as a hydrolytic step on the availability of these peptides in grains has yet to be investigated. In this study, quinoa was malted under systematic temperature, moisture, and time variations. In the resulting malts, the DPP-IV inhibition reached a maximum of 45.02 (±10.28) %, whereas the highest overall concentration of literature-known inhibitory peptides was 4.07 μmol/L, depending on the malting parameters. After *in vitro* gastrointestinal digest, the inhibition of most malts, as well as the overall concentration of inhibitory peptides, could be increased significantly. Additionally, the digested malts showed higher values in both the inhibition and the peptide concentration than the unmalted quinoa. Concerning the malting parameters, germination time had the highest impact on the inhibition and the peptide concentration after digest. An analysis of the protein sizes before and after malting gave first hints toward the origin of these peptides, or their precursors, in quinoa.

## 1 Introduction

Diabetes mellitus type 2 is one of the most common metabolic disorders in modern society, and its occurrence is still increasing.^1^ The disease is caused by a lack of insulin secretion in the pancreas and a loss of sensitivity in tissues responding to insulin. This leads to an increased glucose level in the blood, also called hyperglycemia, which can be lethal. Risk factors for developing diabetes mellitus type 2 are lack of exercise, obesity, unhealthy diet, and oxidative stress.^2,3^ The peptide hormones responsible for the production and release of insulin are the so-called incretins, namely, glucagon-like peptide 1 (GLP-1), released from the ileum and colon, as well as glucose-dependent insulinotropic peptide (GIP), released from the duodenum and proximal jejunum.^1^ Their production in the small intestine is dependent on glucose uptake. However, these peptide hormones are degraded within 2–7 min by the enzyme dipeptidyl peptidase IV (DPP-IV). The action of this serine peptidase in insulin control can be influenced by DPP-IV inhibitors, which reduce its activity and thus lead to an enhanced release of insulin into the blood.^2,4,5^

Synthetic DPP-IV inhibitors, so-called gliptins, have been used as oral therapy for diabetes for over a decade. Synthetic DPP-IV inhibitors, however, can lead to side effects, such as nasopharyngitis, pancreatitis, and upper respiratory tract infections.^6,7^ Thus, investigations have been conducted to identify DPP-IV inhibitory peptides, which positively impact the insulin level without causing adverse effects. However, their effect on the enzyme is reduced compared to gliptins.^7,8^ Several sources for these peptides have been identified, including rice bran,^9^ wheat gluten,^10^ amaranth,^11,12^ and quinoa.^12−14^ The inhibitory peptides reported in the literature vary in length (2–17 amino acids)^15^ and amino acid sequence, both factors influencing the strength of the inhibition, generally given as IC_50_ value. Overall, peptides with high inhibitory effects often carry tryptophan, threonine, asparagine, or valine at the N-terminus or proline at their penultimate position.^16^ Two of the most effective peptide inhibitors are IPI (IC_50_ = 4.5 μm) and WR (IC_50_ = 20 μm).^16−18^

In general, DPP-IV inhibitory peptides are released from proteins during hydrolysis. In previous studies, this process has been performed with different enzymes like alcalase,^19,20^ trypsin,^21,22^ or flavourzyme.^19,20,23^ In some studies, the inhibitory effect of hydrolysates produced by a combination of multiple enzymes was investigated.^15,19,24^ Another factor affecting the DPP-IV inhibitory activity *in vivo* is the further hydrolysis of peptides during gastrointestinal digestion, which can be simulated *in vitro* through a digest with pepsin, trypsin, and chymotrypsin. Although this process might be necessary for predicting how the food hydrolysate is taken up in the body and if it retains its inhibitory effect, it has only been performed in a limited number of studies.^15,25−28^

Another type of protein hydrolysis, naturally occurring in plant seeds, is germination. During this, storage proteins are degraded to obtain amino acids essential for metabolic processes, such as seedling growth.^29^ Germination can be technologically used and controlled *via* malting, consisting of steeping, germination, and kilning. The role of steeping is the water uptake by the mature grain to induce and accelerate germination,^30,31^ which is conducted at a defined temperature, air humidity (moisture), and time. At kilning, the grains are dried for enhanced storage stability, and the biochemical processes are fixated.^32^ During germination, storage proteins are hydrolyzed into (oligo-)peptides by endopeptidases, mainly of the cysteine class, and degraded into dipeptides and amino acids by carboxypeptidases.^33^ Especially after short germination, when the degradation of storage proteins into peptides has only started, the inhibitory potential of the grains increases, as has been shown for cowpea beans before.^34^ You et al. investigated the influence of short germination times (2 h) and subsequent enzymatic hydrolysis on the DPP-IV inhibitory potential of different quinoa cultivars. They showed that white quinoa, germinated for 2 h and additionally hydrolyzed by pepsin and trypsin, had the highest amount of DPP-IV inhibitory peptides and the most potent effect on DPP-IV.^14^ No study so far has investigated the influence of systematically varied malting conditions on the DPP-IV inhibitory effect of (pseudo)cereals. Malting conditions of quinoa have been investigated^35^ but not yet optimized for the enrichment of bioactive peptides. This investigation will enable us to selectively increase the concentration of these naturally occurring bioactive peptides on a large scale.

In the present study, white quinoa was malted under different conditions. The malts were investigated using ultra high-performance liquid chromatography-tandem mass spectrometry (UHPLC-MS/MS), DPP-IV inhibitory assay, and automated electrophoresis. The aim of the study was (i) to investigate the effect of malting on DPP-IV inhibitory peptides in quinoa, (ii) to determine the influence of the combination of technological hydrolysis and simulated gastrointestinal digestion, and (iii) to apply a new proteomics approach for the quantification of DPP-IV inhibitory peptides.

## 2 Materials and Methods

### 2.1 Chemicals

Formic acid and acetonitrile in LC-MS grade were obtained from Merck (Darmstadt, Germany). The synthetic reference peptides (purity ≥80%) were purchased from Peptides and Elephants (Hennigsdorf, Germany). Deuterium oxide (D_2_O) was supplied from Sigma-Aldrich (Steinheim, Germany), and deionized water for chromatography was purified using a Milli-Q Reference A+ system from Merck Millipore (Schwalbach, Germany). RotiQuant assay solution was purchased from Carl Roth (Karlsruhe, Germany). The quinoa grains were obtained from Münchner Bauern Genossenschaft (Munich, Germany). Buffer constituents were purchased from Carl Roth (Karlsruhe, Germany) or Merck.

### 2.2 Quinoa Malting

The conditions for quinoa malting were based on preliminary research^35^ and further developed using a Response Surface Methodology type setup. The following conditions were chosen for the malting procedure: Temperatures at 8, 11.5, or 15 °C; moistures at 44, 48, or 52%; and germination time of 3, 5, or 7 days. On the first day of malting, 500 g of white quinoa (*Chenopodium quinoa* Willd.) were steeped in water for 5 h and put in climate chambers with the temperatures indicated in Table 2 and a humidity of 95%. On day 2, the grains were weighed, thoroughly mixed, and again steeped if necessary for high moisture. On days three and four, the grains were weighed, and if the weight was too low, water was added to reach the calculated weight for the respective moisture. Afterward, it was thoroughly mixed. The five- and seven-day samples were mixed twice daily for the following days until day 5 or 7, respectively. Kilning was done at 50 °C for 16 h, 1 h at 60 °C, and 5 h at 74 °C, followed by removing the germs from the grains.

**Table 1:** Recovery Rates at Three Different Levels at Different Spiking Levels (30, 50, 100, 200, and 300%)

	recovery rate				
compound	30%	50%	100%	200%	300%
APF	116.59	116.48	119.39	120.89	120.84
HI	107.40	99.45	95.34	98.77	93.08
HL	105.81	105.02	89.19	114.90	115.05
RL	112.98	99.29	111.69	97.80	107.53
RI	118.07	115.07	94.72	88.57	114.05
IR	89.51	85.28	92.63	96.91	93.55
LR	n. d.	113.96	n. d.	114.23	119.47

### 2.3 Protein and Peptide Extraction

500 mg of the quinoa malt or the unmalted grains was milled with a Tissuelyser II from Qiagen (Hilden, Germany) for 1 min at 25 Hz to extract proteins and peptides from cereals and pseudocereals. The flour was then mixed with ammonium bicarbonate buffer (100 mM, pH 8.0) at 4 °C and stirred at 300 rpm for 30 min. Afterward, the mixture was centrifuged for 20 min at 10,000*g*, and the supernatant was filtered through 0.45 μm filters (Macherey-Nagel, Düren, Germany). Before the activity-based assay, the extracts were heat-inactivated at 90 °C for 10 min to denature enzymes that might interfere. The protein concentration was measured using the RotiQuant assay (Carl Roth). For this, 50 μL of the protein solution was mixed with 200 μL diluted RotiQuant solution, and after 5 min of incubation at room temperature, the absorption was measured at 595 nm. The protein concentration was then adjusted to 1 mg/mL for further procedures.

### 2.4 Simulated Gastrointestinal Digest

The simulated gastrointestinal digest was performed as described by Minekus et al.^36^ In short, 0.5 mL of the sample was mixed with 375 μL of simulated gastric fluid, 80 μL of pepsin stock solution, 0.25 μL of CaCl_2_ (0.3 M), and 5 μL of HCl (25%) and filled up to 1 mL with water. The mixture was then incubated at 37 °C and 300 rpm in a Biometra TSC Thermoshaker (Goettingen, Germany) for 2 h. After this, 550 μL of simulated intestinal fluid, 250 μL of the enzyme stock (trypsin and chymotrypsin), 2 μL of CaCl_2_ (0.3 M), and 198 μL of deionized water were added. The mixture was again incubated for 2 h at the above-mentioned conditions. Hereafter, the enzymes were heat-inactivated at 90 °C for 10 min.

### 2.5 Fluorescence-Based DPP-IV Inhibitory Assay

The fluorescence-based assay from Cayman Chemical Company (Michigan) was used to analyze the inhibition of DPP-IV.^22^ 10 μL of the sample was mixed with 10 μL of a human DPP-IV recombinant, 50 μL of the assay substrate (H-Gly-Pro-aminomethyl coumarin), and 30 μL of assay buffer (20 mM Tris–HCl, 100 mM NaCl, 1 mM EDTA, pH 8.0). This was incubated at 37 °C for 20 min while measuring the fluorescence intensity at 450 nm after excitation at 350 nm every 30 s in a Cytation 5 microplate reader (Biotek, Winooski). For each measurement, a background control without the enzyme and a full activity control without inhibitor or extract was added. The results were analyzed using Microsoft Excel (v. 365; Redmont), where the slope over time was analyzed for every sample, minus the background control. The percentage inhibition was then calculated in relation to the full activity control without the inhibitor.

### 2.6 Lab-on-a-Chip Protein Analysis

For the analysis of the proteins contained in the quinoa malt samples, 400 μL of urea buffer (2 M urea, 15% glycerol, 0.1 M Tris, 0.1 M DTT, pH 8.8) was added to 40 mg of quinoa flour. The samples were then mixed and put into a sonication bath for 10 min. After this, they were centrifuged at 10,000*g* for 10 min, and 2 μL of a 1:2 dilution of the supernatant was transferred to a prepared protein chip (Protein 230 Kit, Agilent, Santa Clara). The analysis was performed using Agilent 2100 Expert (v. B.02.11.SI824).^37^

### 2.7 Targeted Proteomics

#### 2.7.1 Ultra High-Performance Liquid Chromatography-Tandem Mass Spectrometry (UHPLC–MS/MS)

All targeted measurements were performed on an ExionLC (Sciex, Darmstadt, Germany) connected to a QTrap 6500+ mass spectrometer (Sciex, Darmstadt, Germany) running in positive electrospray ionization (ESI) and multiple reaction monitoring mode (MRM). Chromatography was acquired on a 2.1 mm × 150 mm, 1.7 μm ACQUITY UPLC BEH Amide column (Waters, Aschaffenburg, Germany) and a gradient of 5 mM ammonium acetate in water, pH 2 (solvent A), and 5 mM ammonium acetate in acetonitrile/water (v/v, 95:5), pH 2 (solvent B), and a flow rate of 0.4 mL/min was used: 1 min, 88% B; 12 min; 88% B, 12.5 min, 5% B; 13.5 min, 5% B; 14 min, 88% B; 17 min, 88% B. The column oven was tempered at 40 °C, and the injection volume was 1 μL per sample. The QTrap 6500+ mass spectrometer was operated in a low molecular mass configuration. Ion spray voltage was set at 5500 eV in positive ionization mode, the source temperature was 450 °C, zero grade air served as nebulizing gas (55 psi), heating gas for solvent drying (65 psi), while nitrogen was utilized as curtain gas (35 psi) as well as collision gas (4.5 × 10–5 Torr). Parameters for the declustering potential (DP) and collision energy (CE) for each substance were optimized using Skyline (64-bit, 21.1.0.146). The software was used to compute singly and doubly charged precursor ions with a-, b-, c-, and x-, y-, z-product ions.^38^ Instrument control and data acquisition were performed using Sciex Analyst software (v 1.6.3). All data evaluation was completed with Skyline software (64-bit, 21.1.0.146) and MultiQuant (v. 3.0.2, Sciex).

#### 2.7.2 In Silico Development of Selected Reaction Monitoring (SRM) Methods

The software Skyline (64-bit, 21.1.0.146) was used to calculate and optimize the selected reaction monitoring (SRM) methods for identifying DPP-IV inhibitory peptides *in silico.*(38) The inhibitory target peptides were obtained from the literature, ranging from 2–15 amino acids in length (Table S1). The list of references can be found in the Supporting Information. In total, 1703 mass transitions were calculated for singly and doubly charged precursor ions, with a-, b-, c-, and x-, y-, z-product ions, and Q1 and Q3 resolutions were set to unit (0.7). Using a maximum of 200 transitions per method, 14 multiple SRM methods (Table S2) were exported from Skyline and used to analyze selected quinoa malt samples. The digested and undigested quinoa malt samples were screened using the *in silico*-calculated UHPLC-MS/MS methods. After UHPLC-MS/MS analysis, the data were imported into Skyline and analyzed manually to evaluate the signal quality. Peptides with equivocal peaks were removed, resulting in seven target peptides. After filtering the transition list, the five most intense transitions of each peptide were selected, and their collision energy (CE), declustering potential (DP), and collision cell exit potential (CXP) were optimized directly on MS using a syringe pump. The final method contained 35 mass transitions for the seven DPP-IV inhibitory peptides (APF, HI, HL, RI, RL, IR, and LR) and is available in the Supporting Information (Table S3).

### 2.8 Quantitation of DPP-IV Inhibitory Peptides

Aliquots (1 μL) of *in vitro* gastrointestinally digested or undigested quinoa malt samples, unmalted quinoa, both digested and undigested, and of the enzyme control from chapters 2.3 and 2.4 were directly injected into the UHPLC–MS/MS system. Quantitation was performed using external calibration with standard solutions containing the target peptides in the range of 1000–0.005 μM (17-point calibration). Therefore, a stock solution of the peptides APF (9.33 mM), HI (9.98 mM), HL (9.87 mM), IR (16.94 mM), LR (27.48 mM), RI (20.91 mM), and RL (21.36 mM) was prepared in water, after their exact concentrations were determined using quantitative ^1^H nuclear magnetic resonance (qHNMR). This stock solution was then diluted 1:2, 1:5, 1:10, 1:20, 1:50, 1:100, 1:200, 1:500, 1:1000, 1:2000, 1:5000, 1:10000, 1:20000, and 1:50000 with water. Calibration curves were obtained by plotting the concentration of the analyte *versus* the peak area of the analyte using linear regression.

For recovery experiments, the stock solution was spiked into digested (**2**, **9**, **18**, **19**, **23**) and undigested (**2**, **19**, **15**, **12**, **8**) quinoa malt samples referring to additional peptide concentrations of 30, 50, 100, 200, and 300% as triplicates. To determine the recovery rate, the concentration of the individual analytes calculated in the spiked samples was corrected by the amount determined in the control sample. This value was divided by the predicted concentration and the recovery rate was given in %. The quantitative analysis was performed using the above-mentioned UHPLC/MS-MS parameters. The calculated recovery rates are shown in Table 1.

To evaluate the intraday and interday precision, three spiked aliquots of the same quinoa malt were analyzed. For intraday precision, the spiked samples were analyzed on the same day. To determine the interday precision, the same quinoa samples were analyzed on three different days. The intraday and interday precision were determined as relative standard deviations and are shown in Table S4.

For the determination of the limit of detection (LoD) and limit of quantitation (LoQ), the stock solution was further diluted and was evaluated using MultiQuant (v. 3.0.2). The LoD was determined at a signal-to-noise ratio of 3, and the LoQ was determined at a signal-to-noise ratio of 10.

### 2.9 Quantitative Proton Nuclear Magnetic Resonance Spectroscopy (qHNMR)

Quantitative ^1^H nuclear magnetic resonance (qHNMR) experiments were performed on a Bruker AVANCE III 400.13 MHz system (Bruker, Rheinstetten, Germany), equipped with a Broadband Observe BBFO plus probe. The peptides were dissolved in D_2_O, and an aliquot of 600 μL was filled in 5 × 178 mm^2^ NMR tubes (USC tubes, Bruker, Faellanden, Switzerland). For quantitative ^1^H NMR experiments, the spectrometer was calibrated using the ERETIC 2 software tool using the PULCON methodology, as reported earlier.^39^ The specific proton resonance signal of l-tyrosine (6.68 mM) at 7.10 ppm (m, 2H) was used for external calibration. For data analysis, each peptide was assigned a specific signal for integration.^39^ Instrument control and data processing were performed using Topspin software (version 3.3; Bruker).

### 2.10 Statistical Analysis

#### 2.10.1 Design of Experiments

The malting experiment was designed using the response surface methodology (RSM).^40^ A face-centered cube was chosen, where the center point was conducted in triplicates, the axial conditions in duplicates, and the corner conditions as single samples. The variables were germination temperature (8–15 °C), germination time (3–7 days), and moisture (44–52%).

#### 2.10.2 Analysis of Experiments

An outlier analysis was performed using the Grubbs test. The data were normalized, and a stepwise regression was performed with JMP Pro 16 (SAS Institute, Cary). The coefficients were tested on significance, and the formulas described in the Supporting Information (Table S5) were gained from the analysis. The root-mean-square deviation (RMSE) was used to determine the goodness of fit.
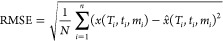
1where, *x*(*T_i_*, *t_i_*, *m_i_*) = measured value at temperature *T_i_*, time *t_i_*, and moisture *m_i_*. *x̂*(*T*_*i*_, *t*_*i*_, *m*_*i*_) = predicted value at temperature *T_i_*, time *t_i_*, and moisture *m_i_*.

## 3 Results and Discussion

### 3.1 Inhibitory Potential of Different Quinoa Malts

Quinoa was malted at different conditions, namely, 8–15 °C, 44–52%, and 3–7 days. After kilning, the malts were analyzed regarding their effect on human DPP-IV *in vitro*, both before and after *in vitro* gastrointestinal digest. Table 2 shows the results of the inhibition measurements of the different malt extracts before and after *in vitro* gastrointestinal digest. Concerning the undigested samples, the maximum inhibition of 45.02% can be observed for the malt germinated at 52%, 15 °C, and 3 days (baskets 24 and 25). In contrast, no inhibitory effect was achieved by the samples malted at 44%, 8 °C, and 3 days (baskets 6 and 7) and 48%, 11.5 °C, and 3 days (basket 16). A reason for the absent inhibitory effect of the mentioned malts might be the short germination time at the lower temperature compared to the sample with the highest inhibitory potential. In comparison, unmalted and undigested quinoa extracts showed an inhibitory effect of 15.15%. Thus, at lower temperatures, the inhibitory peptides present in the unmalted quinoa seem to be decreased, possibly due to the protease activity not being high enough to release new bioactive peptides from storage proteins. At a higher temperature of 15 °C, the degradation of the storage proteins led to an increase in DPP-IV inhibitory peptides. A comparison of the highest and lowest germination conditions through a *t*-test only showed a significant difference between the temperatures 8 and 15 °C but not the time or the humidity (*p* < 0.01).

**Table 2:** Measured Inhibitory Effect of Quinoa Malt Extracts and the *In Vitro* Gastrointestinal Digests^a^

baskets	moisture [%]	temperature [°C]	time [days]	inhibition before digest [%]	inhibition after digest [%]
1–2	44	8	7	2.80 (±2.39)	29.91 (±1.66)
3–4	52	8	7	8.43 (±5.01)	36.65 (±1.42)
5	48	8	5	22.45 (±0.91)	33.77 (±0.16)
6–7	44	8	3	0.00 (±0.00)	30.55 (±5.64)
8–9	52	8	3	0.96 (±1.66)	30.53 (±2.29)
10	48	11.5	7	10.99 (±0.32)	28.59 (±0.60)
11	44	11.5	5	2.76 (±0.20)	34.68 (±0.56)
12–14	48	11.5	5	14.92 (±17.77)	35.01 (±11.04)
15	52	11.5	5	9.31 (±1.95)	37.69 (±1.01)
16	48	11.5	3	0.00 (±0.00)	37.00 (±0.66)
17–18	44	15	7	15.98 (±15.77)	29.46 (±3.69)
19–20	52	15	7	13.46 (±12.29)	37.20 (±12.00)
21	48	15	5	17.29 (±2.70)	24.28 (±1.24)
22–23	44	15	3	24.92 (±5.40)	31.69 (±6.03)
24–25	52	15	3	45.02 (±10.28)	41.86 (±11.00)
A				15.15 (±1.63)	
B					16.66 (±2.02)
C					4.79 (±0.92)

a Given are the means of the technical replicates as well as the standard deviation. A = unmalted quinoa, B = digested unmalted quinoa, C = digested buffer control.

After simulated gastrointestinal digest, the sample showing the highest inhibitory effect (41.86%) was again the one malted at 52%, 15 °C, and 3 days (baskets 24 and 25), whereas basket 10 (48%, 11.5 °C, 7 days) showed the lowest inhibitory potential (28.59%). The statistical analysis between the singular germination conditions shows only a significant difference between the highest and the lowest moisture (*p* < 0.05). When the difference between the undigested and the digested malts is considered, the inhibitory potential of most samples after digest is significantly higher than in the undigested samples, the highest increase occurring in basket 16 (48%, 11.5 °C, 3 days). This increase in inhibitory potential after the digest might be explained by the degradation of noninhibitory oligopeptides generated through the hydrolysis of storage proteins during malting. The *in vitro* gastrointestinally digested, unmalted quinoa showed an inhibitory effect of 16.66%, which lies beneath the lowest inhibitory effect of digested quinoa malts measured here but again higher than the undigested quinoa adjunct. This finding confirms the hypothesis that oligopeptides are produced during malting, which are further degraded into inhibitory peptides during gastrointestinal digestion. This process cannot occur in unmalted quinoa, and only little degradation of the storage proteins takes place. Hence, the malting combined with a gastrointestinal digest positively influences the occurrence of DPP-IV inhibitory peptides in quinoa, while malting or digestion alone has a minor influence. Additionally, a buffer control of the digest was performed, where the extraction buffer was treated with gastrointestinal enzymes. This resulted in a low DPP-IV inhibition of 0.56%, which might be due to a potential self-digest of the peptidases, leading to potentially inhibitory peptides. Especially a digest of pepsin during the intestinal phase is possible since the enzyme is inactive at these conditions.

The results of the inhibition measurements were then related to the original protein concentrations of the quinoa samples, indicating the absolute potential the malt would have without being set to a protein concentration of 1 mg/mL, as conducted with all samples above. The data suggest that before the *in vitro* gastrointestinal digest, the most potent inhibitory sample is still the one germinated at 52% and 15 °C for 3 days. However, after the simulated human digest, the highest total inhibition can be seen in the malt germinated at 52% and 8 °C for 7 days (Table S6). Thus, this sample has a higher potential to release many DPP-IV inhibitory peptides in the intestine of diabetic patients due to its high protein concentration and total inhibitory effect.

The results of the inhibition measurement were statistically analyzed by stepwise regression to identify the parameters of germination having the highest impact on the change of inhibitory effect between unmalted and malted quinoa. In the undigested quinoa malt, the germination parameter having the highest impact on the inhibitory potential was the time (*p* < 0.05), followed by the nonsignificant terms of temperature and the cross term of both. After the simulated gastrointestinal digest, on the other hand, the parameter showing the strongest influence on the DPP-IV inhibitory potential was the humidity (*p* < 0.1), followed by the nonsignificant parameters humidity squared and the cross term of temperature and moisture. This suggests that before the *in vitro* gastrointestinal digest, the influence of the single parameters during malting is higher, and optimum malting conditions are more accessible than after digestion. However, during the enzymatic hydrolysis, the effects of the malting parameters on the inhibitory potential are leveled out, leaving an overall increased inhibition without a clear trend toward optimum malting conditions.

**Figure 1:**
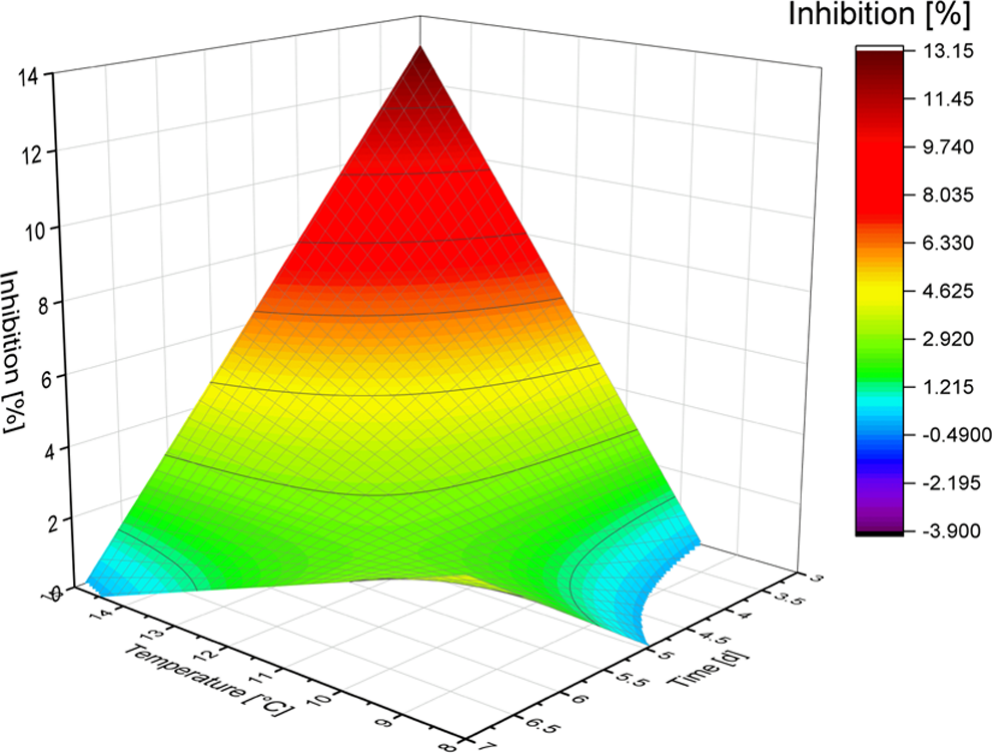
Surface plot of the calculated inhibitory effects for undigested malt germinated at different temperatures and for varying times. The moisture was excluded from this plot because of the statistical analysis, showing that it has no significant influence.

**Figure 2:**
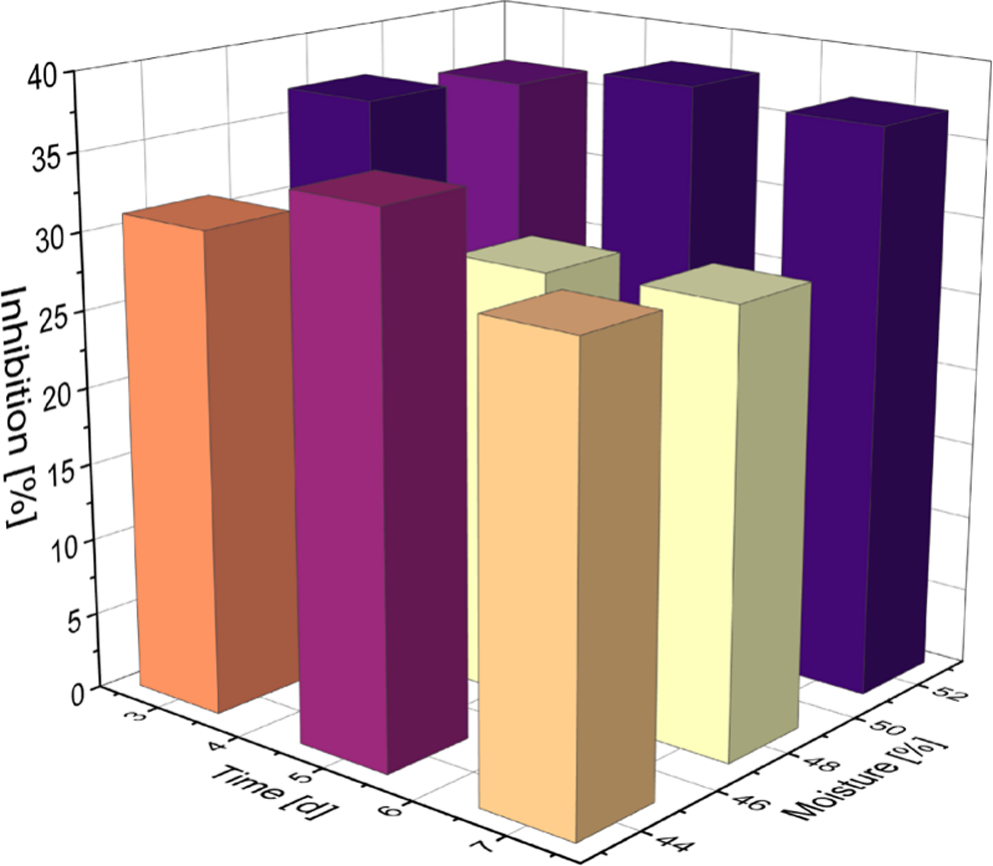
Bar chart of the measured inhibitions by *in vitro* gastrointestinal digested malts germinated at different moistures over varying time. The values shown here are the mean values from the grains malted at different temperatures.

During malting, storage proteins are degraded into peptides and amino acids needed for seedling growth.^41^ Research on the influence of germination on the occurrence of DPP-IV inhibitory peptides has so far only been conducted in beans and quinoa for short-term germination. de Souza Rocha et al. investigated the influence of bean germination (0, 24, or 48 h) on the occurrence of DPP-IV inhibitory peptides. They showed that especially short-term germination (24 h) of cowpea beans and subsequent Alcalase treatment leads to increased concentrations of DPP-IV inhibitors compared to nongerminated samples.^34^ This effect could not be reproduced in common beans.^42^ You et al., on the other hand, described the impact of short germination times on the DPP-IV inhibitory potential of quinoa.^14^ They showed that short-term germination of 2 h was advantageous for producing DPP-IV inhibitory peptides, while 24 or 48 h germination resulted in lower inhibitory effects. The drawback of these studies is that they only focused on the germination time but not the temperature or moisture. The present results also indicate that, before simulated gastrointestinal digest, the germination time plays a significant role, showing the highest DPP-IV inhibitory potential in malts germinated for 3 days (Figure 1). By simulating the gastrointestinal digest *in vitro*, it was possible to predict which effect quinoa malt might have after ingestion by diabetic patients. Here, the moisture during germination seems to be the only significant parameter. The measured inhibitions are visualized in Figure 2, showing the highest potential at a moisture of 52% and a germination time of 5 days. The present results show that a targeted increase of DPP-IV inhibition can be achieved by choosing the correct malting conditions. Although the predicted inhibition in the human body is higher than with the unmalted quinoa, the bioavailability of the peptides or the quinoa extract has not yet been investigated. It can be suggested from the literature that the peptides have a high chance of being taken up in the intestine and that the presence of other molecules, such as sugars or amino acids, might enhance this uptake,^43^ leading to the assumption that the quinoa malt extract has the potential to be a nutraceutical with a high impact on the insulin secretion in diabetes type II patients.

### 3.2 Targeted Analysis of Literature-Known DPP-IV Inhibitory Peptides Using UHPLC-MS/MS

After analyzing the DPP-IV inhibitory potential of the malt extracts as well as the unmalted quinoa, the samples (undigested as well as after simulated gastrointestinal digest) were investigated concerning the concentrations of literature-known DPP-IV inhibitory peptides. For this purpose, a rapid, selective, and sensitive quantification method for DPP-IV inhibitory peptides using UHPLC-MS/MS was developed.

#### 3.2.1 Method Development for UHPLC-MS/MS Measurements

**Figure 3:**
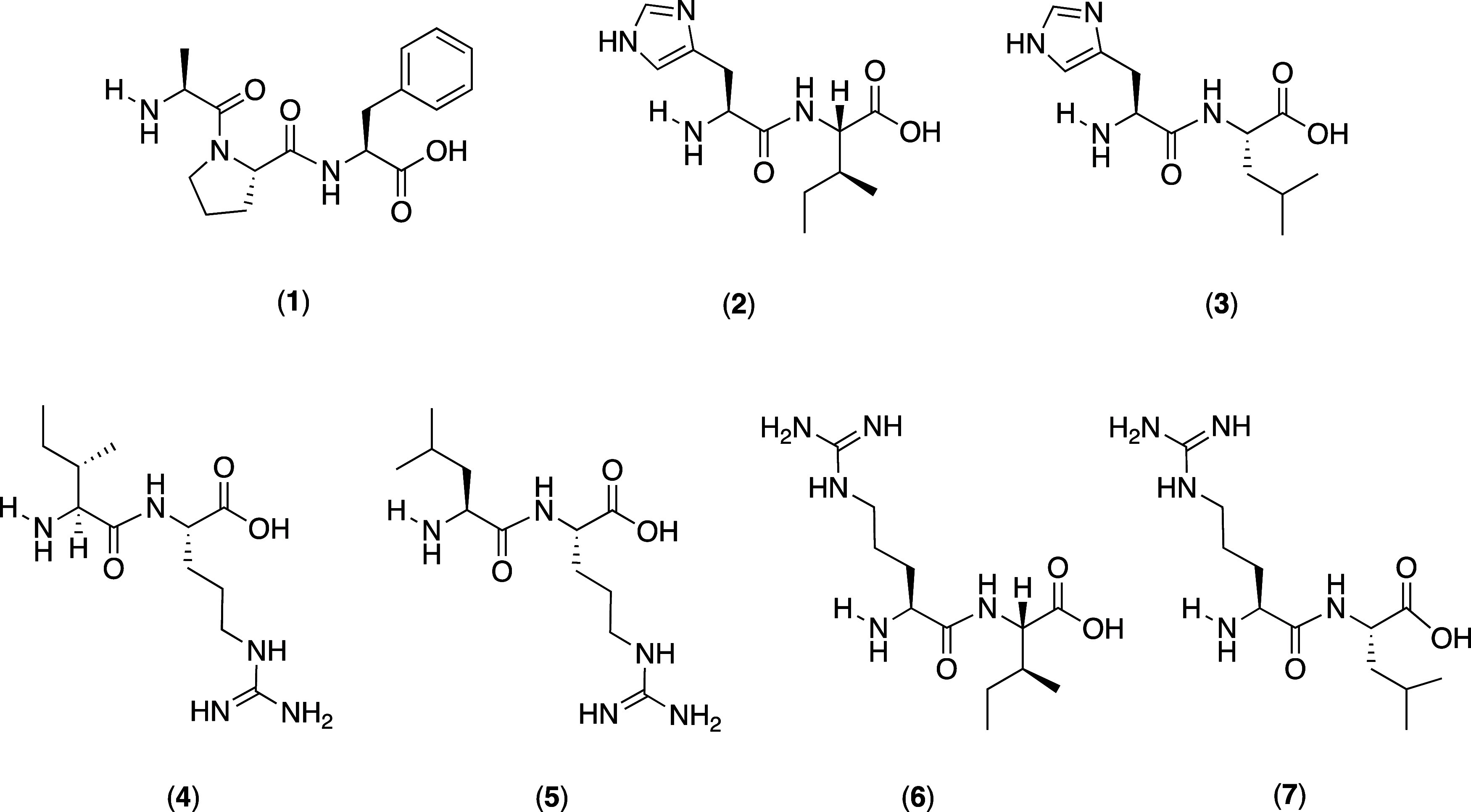
Chemical structures of DPP-IV inhibitory peptides analyzed in digested quinoa malt: APF (1), HI (2), HL (3), IR (4), LR (5), RI (6), and RL (7).

Several DPP-IV inhibitory peptides have been identified in different food sources like salmon,^44^ oat,^5^ or dairy^45^ products. In order to identify those peptides analytically in quinoa, 1703 mass transitions of 153 literature-known peptides (Table S2) were calculated *in silico* using the software Skyline. After the digested and undigested samples were screened using the *in silico* developed UHPLC-MS/MS methods, peptides were sorted out by application of different criteria: only peptides with high signal quality (signal-to-noise ratio >3) and at least five mass transitions were kept, while peptides with ambiguous signals were neglected. Following this approach, seven literature-known DPP-IV inhibitory peptides, APF, HI, HL, IR, LR, RI, and RL, were identified in digested quinoa malt (Figure 3). Afterward, the MS/MS parameters were optimized in ESI^+^ mode to quantify the peptides with maximum sensitivity. To separate the isobaric dipeptides, differing only by the presence of leucine or isoleucine, a suitable column material, and appropriate UHPLC parameters were selected. Due to the high polarity of these short-chained peptides, the best chromatographic separation was obtained on an ethylene-bridged hybrid amide column, and optimization of the solvent gradient enabled baseline separation of all peptides.

For external quantification, varying analyte concentrations between 1000 and 0.001 μm were measured. Linear calibration curves were obtained by plotting the peak areas of each analyte against its concentration. Thereby, correlation coefficients >0.99 were obtained for all calibration curves.

#### 3.2.2 Method Validation Experiments

**Figure 4:**
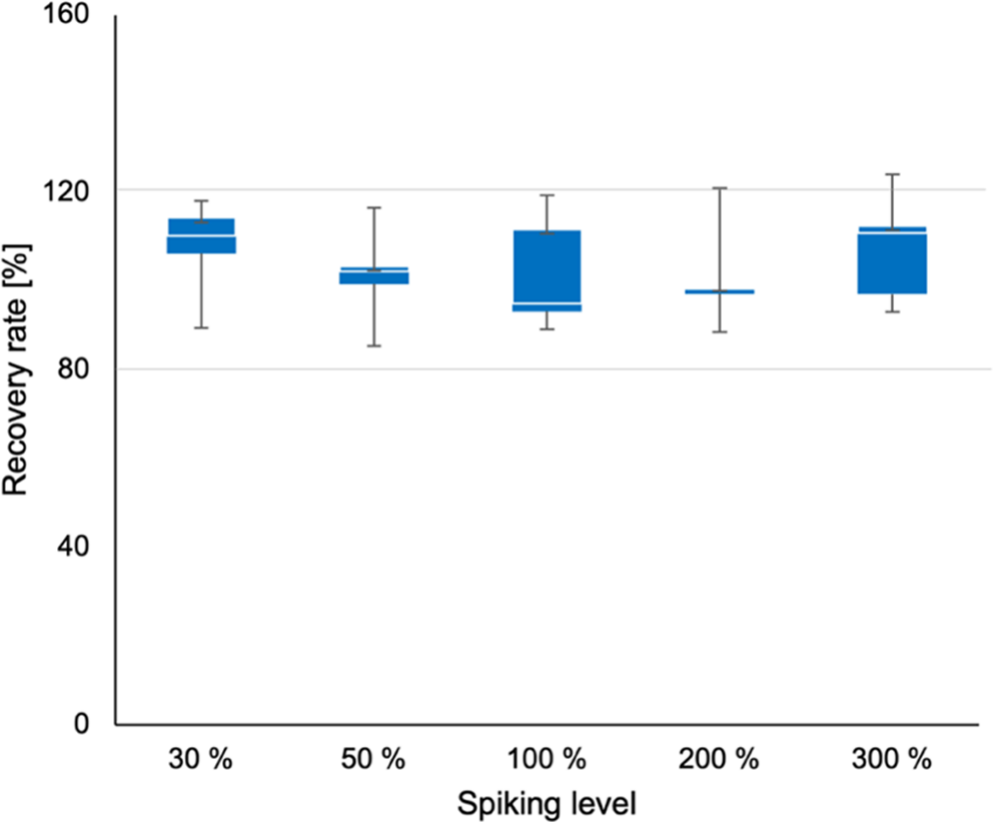
Determination of recovery rate according to DIN 32645 at different spiking levels (30, 50, 100, 200, and 300%).

After method development, accuracy experiments were performed to verify the trueness and robustness of the newly developed quantification method. Five different concentration levels were spiked into selected samples of the digested and undigested quinoa malt covering the complete calibration range, and the recovery rates were determined for the target peptides **1**-**7** (Figure 3). Recovery rates in the range of 91.58% (**4**), 98.81% (**2**), 105.86% (**7**), 105.99% (**3**), 106.09% (**6**), 114.23% (**5**), and 118.84% (**1**) were determined (Figure 4 and Table 1)

In addition, intra- and interday studies were performed to evaluate the precision of the developed LC-MS/MS method. For this purpose, the spiked samples were measured directly after each other on the same day (intraday) and three different days (interday). Intraday precision ranged from 2.14 to 40.51%, and interday precision from 1.86 to 11.06% (Table S4), confirming a high precision between and within different days and the applicability of the UHPLC-MS/MS method for quantitative analysis.

Moreover, the limits of detection (LoDs) and limits of quantitation (LoQs) were calculated for the seven peptides (Table S4). The LoDs ranged from 0.0007 μM (APF) to 0.0433 μM (LR). The LoQs of the peptides were in the range of 0.0031 μM (HI)–0.1182 μM (RI). Compared to the IC_50_ values described in the literature of APF (65.8 μM) and HL (143.2 μM),^46^ the LoD and LoQ were far below these, proving it to be a highly sensitive quantification method.

### 3.3 Quantitation of DPP-IV Inhibitory Peptides in (Hydrolyzed) Quinoa Samples

**Figure 5:**
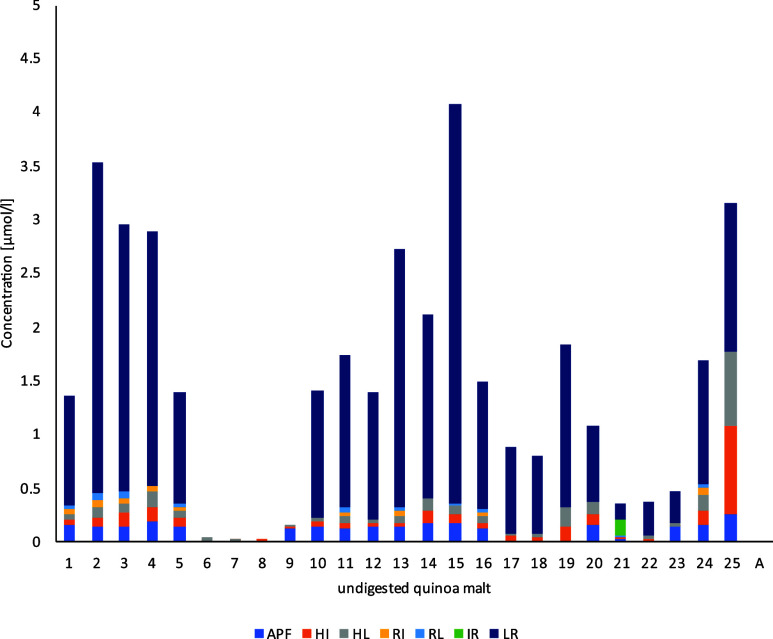
Concentrations of identified DPP-IV inhibitory peptides in undigested quinoa malt samples (basket 1–25), A = unmalted quinoa.

To date, very few DPP-IV inhibitory peptides have been identified and quantified in quinoa.^13,24,47−50^ Using a newly developed UHPLC-MS/MS screening method, covering 153 peptides known from the literature, seven literature-known DPP-IV inhibitory peptides (APF, HI, HL, IR, LR, RI, and RL; Figure 3) could be identified and quantified in digested quinoa malt samples for the first time. Furthermore, to our knowledge, these peptides have not previously been identified and quantified in quinoa. Among these peptides, the concentrations of APF, HI, IR, LR, and RL in undigested malted quinoa samples were lower than those in the *in vitro* digested samples. To predict the stability of quinoa malt peptides in the gastrointestinal tract, human digestion was simulated in the present study, and the concentrations of literature-known DPP-IV inhibitory peptides were measured hereafter. In addition to the hydrolyzed samples, the unmalted and undigested quinoa **A** was analyzed, showing no measurable amount of peptides. In contrast, all seven DPP-IV inhibitory peptides could be quantified at low concentrations in the unhydrolyzed malt samples (Table 3). The average concentrations of the identified peptides were 0.04 μM (RL), 0.05 μM (RI), 0.09 μM (HI), 0.10 μM (HL), 0.15 μM (APF), and 1.42 μM (LR), while the peptide IR could only be quantified in sample 21 (0.16 μM) (Figure 5 and Table S7).

**Table 3:** Average Concentrations of Quantified Peptides (APF, HI, HL, RI, RL, IR, and LR), and Their Relative Standard Deviations in μmol/L in Undigested Samples (1–25, A = Raw Fruit)

basket	APF [μM]	HI [μM]	HL [μM]	RI [μM]	RL [μM]	IR [μM]	LR [μM]
1–2	0.15 (±4.88)	0.07 (±32.64)	0.08 (±47.14)	0.06 (±12.86)	0.06 (±38.57)	n. d.	2.05 (±71.06)
3–4	0.17 (±21.43)	0.13 (±5.66)	0.12 (±43.04)	0.06 (±12.86)	0.03 (±141.42)	n. d.	2.44 (±3.78)
5	0.14^a^	0.08^a^	0.06^a^	0.04^a^	0.03^a^	n. d.	1.04^a^
6–7	n. d.	0.01 (±0.00)	0.02 (±47.14)	n. d.	n. d.	n. d.	n. d.
8–9	0.06 (±141.42)	0.01 (±0.00)	0.01 (±141.42)	n. d.	n. d.	n. d.	n. d.
10	0.14^a^	0.04^a^	0.03^a^	n. d.	n. d.	n. d.	1.20^a^
11	0.13^a^	0.04^a^	0.06^a^	0.04^a^	0.03^a^	n. d.	1.42^a^
12–14	0.15 (±14.19)	0.06 (±65.74)	0.06 (±65.74)	0.02 (±173.21)	0.01 (±173.21)	n. d.	1.76 (±34.38)
15	0.18^a^	0.08^a^	0.08^a^	n. d.	0.02^a^	n. d.	3.71^a^
16	0.12^a^	0.05^a^	0.07^a^	0.04^a^	0.03^a^	n. d.	1.18^a^
17–18	n. d.	0.05 (±15.71)	0.03 (±28.28)	n. d.	n. d.	n. d.	0.77 (±8.32)
19–20	0.08 (±141.42)	0.12 (±30.74)	0.15 (±34.14)	n. d.	n. d.	n. d.	1.12 (±51.77)
21	0.02^a^	0.02^a^	n. d.	n. d.	0.01^a^	0.16^a^	0.14^a^
22–23	0.07 (±141.42)	0.02 (±0.00)	0.03^a^	n. d.	n. d.	n. d.	0.31 (±6.96)
24–25	0.2 (±35.36)	0.49 (±100.60)	0.42 (±90.91)	0.03 (±141.42)	0.03 (±141.42)	n. d.	1.27 (±12.86)
A	n. d.	n. d.	n. d.	n. d.	n. d.	n. d.	n. d.

a No relative standard deviation was calculated due to the RSM calculation scheme. n. d. = not detected.

**Figure 6:**
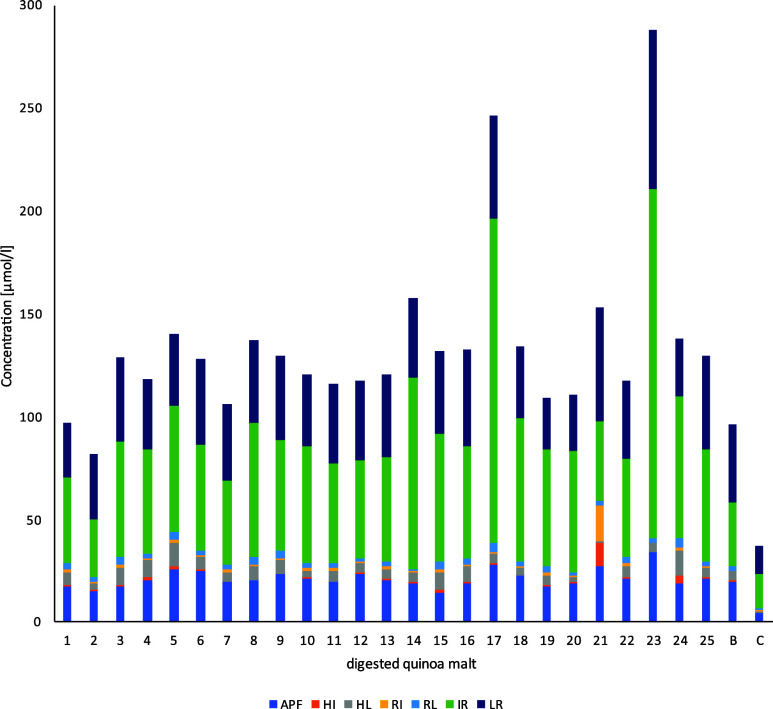
Concentrations of identified DPP-IV inhibitory peptides in digested quinoa malt samples (basket 1–25). B = malted quinoa, C = buffer control.

The peptides were quantified at much higher concentrations in the digested samples than in the undigested samples (Table 4).^51^ The average concentrations of the identified peptides in digested quinoa malt were 1.22 μM (HI), 1.77 μM (RI), 2.66 μM (RL), 5.68 μM (HL), 21.13 μM (APF), 39.48 μM (LR), and 63.62 μM (IR) (Figure 6 and Table S8). Similar findings could be observed after *in vitro* digesting Amaranth malt.^51^

**Table 4:** Average Concentrations of Quantified Peptides (APF, HI, HL, RI, RL, IR, and LR), and Their Relative Standard Deviations in μmol/L in Digested Samples (Baskets 1–25, B = Unmalted Quinoa, C = Enzyme Control)

basket	APF [μM]	HI [μM]	HL [μM]	RI [μM]	RL [μM]	IR [μM]	LR [μM]
1–2	15.97 (±8.06)	0.63 (±38.16)	4.95 (±47.33)	1.14 (±11.84)	2.42 (±46.55)	34.97 (±26.27)	29.31 (±10.42)
3–4	18.63 (±11.35)	1.35 (±17.81)	8.13 (±6.78)	1.27 (±41.2)	3.06 (±40.51)	53.56 (±6.75)	37.51 (±13.35)
5	25.85^a^	1.44^a^	11.26^a^	1.27^a^	3.86^a^	61.43^a^	35.15^a^
6–7	22.18 (±18.11)	0.28 (±18)	5.32 (±22.86)	1.09 (±38.45)	2.32 (±4.58)	46.39 (±15.5)	39.55 (±8.28)
8–9	21.62 (±10.73)	0.42 (±3.37)	6.38 (±6.1)	1.03 (±60.02)	3.65 (±4.26)	59.92 (±13.9)	40.41 (±1.87)
10	21.01^a^	0.45^a^	3.68^a^	1.40^a^	1.85^a^	57.29^a^	34.53^a^
11	19.37^a^	0.53^a^	5.03^a^	1.51^a^	2.14^a^	48.97^a^	38.57^a^
12–14	20.89 (±11.75)	0.63 (±29.38)	4.64 (±4.61)	1.03 (±39.47)	1.45 (±38.74)	64.15 (±39.8)	38.9 (±2.59)
15	14.54^a^	1.24^a^	8.33^a^	1.63^a^	3.87^a^	61.9^a^	40.54^a^
16	18.66^a^	0.63^a^	7.54^a^	1.17^a^	3.27^a^	54.41^a^	47.1^a^
17–18	25.23 (±16.45)	0.53 (±16.01)	4.26 (±16.12)	0.69 (±40.26)	3.14 (±55.85)	113.74 (±54.92)	42.5 (±24.66)
19–20	18.33 (±5.48)	0.54 (±19.83)	3.58 (±37.53)	0.94 (±31.01)	2.08 (±53.71)	58.13 (±2.30)	26.04 (±6.33)
21	27.23^a^	11.28^a^	1.09^a^	17.00^a^	2.70^a^	38.53^a^	54.92^a^
22–23	27.64 (±31.91)	0.44 (±19.28)	4.75 (±13.56)	0.86 (±85.18)	2.43 (±26.19)	108.96 (±79.29)	57.81 (±48.23)
24–25	19.81 (±6.68)	2.31 (±96.12)	8.22 (±63.48)	1.56 (±20.85)	3.10 (±57.94)	62.18 (±16.23)	36.61 (±33.92)
B	19.85^a^	0.25^a^	4.48^a^	0.53^a^	2.01^a^	31.30^a^	37.58^a^
C	4.34^a^	0.17^a^	0.86	0.40^a^	1.16^a^	16.07^a^	13.64^a^

a No relative standard deviation was calculated due to the RSM calculation scheme. n. d. = not detected.

**Figure 7:**
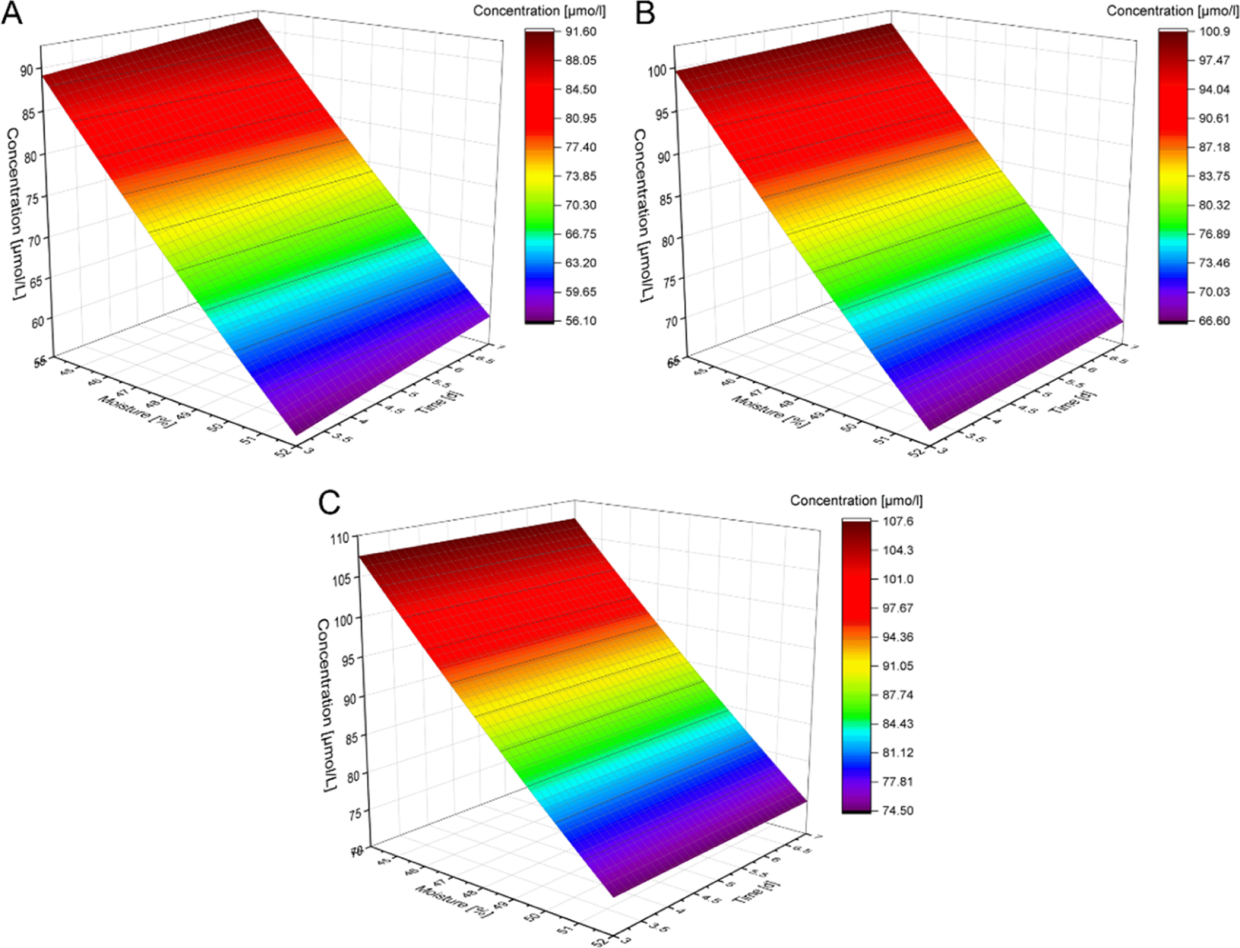
Surface plot of the calculated inhibitory peptide concentrations for malt germinated at different moistures and for varying times; the temperature was set to 8 °C (A), 11.5 °C (B), or 15 °C (C).

**Figure 8:**
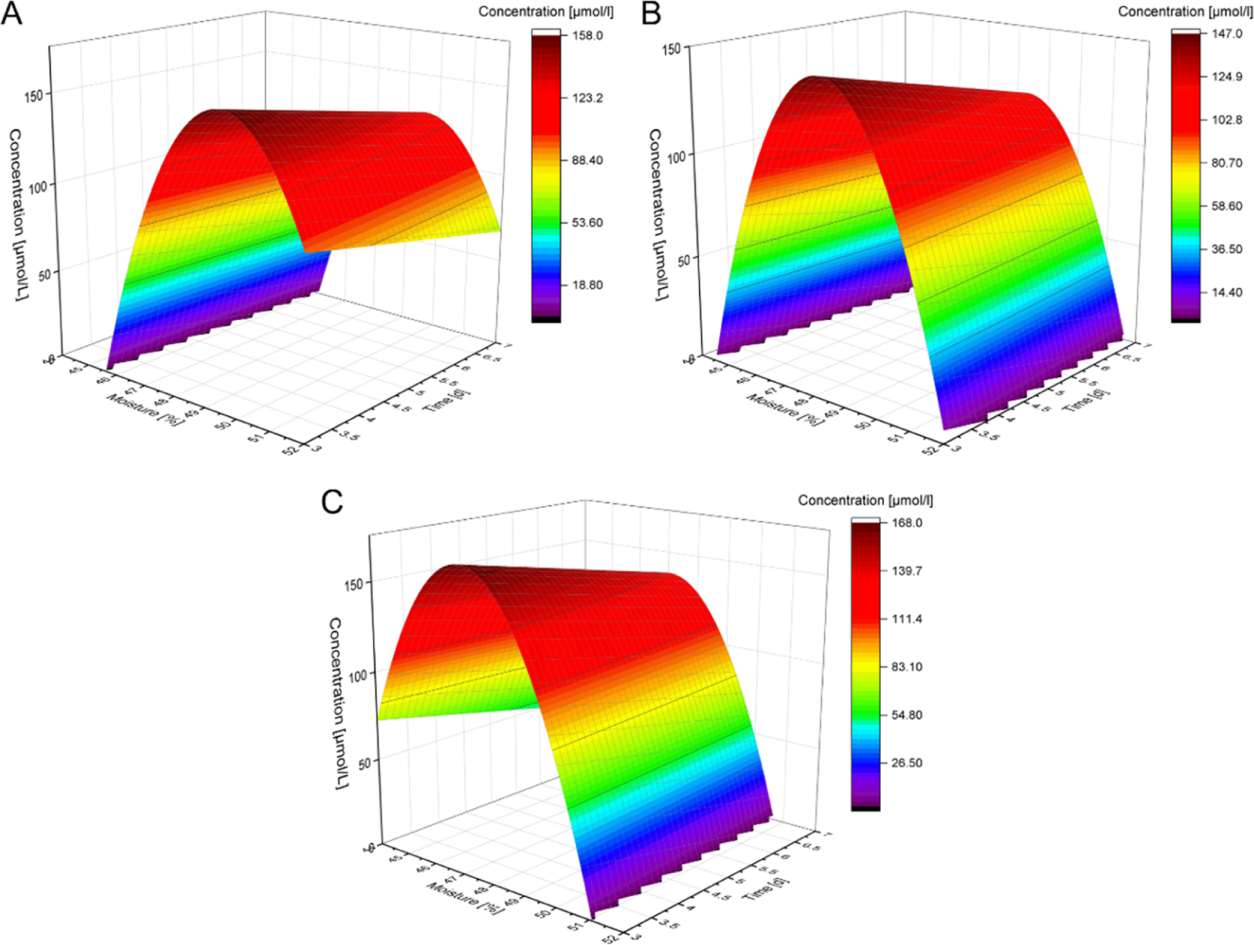
Surface plot of the calculated inhibitory peptide concentrations for *in vitro* gastrointestinally digested malt germinated at different moistures and for varying times; the temperature was set to 8 °C (A), 11.5 °C (B), or 15 °C (C).

Digested basket 21 (5 days, 48%, 15 °C) showed the highest peptide concentration (27.23 μM (APF), 11.28 μM (HI), 1.09 μM (HL), 17.00 μM (RI), 2.70 μM (RL), 38.53 μM (IR), and 54.92 μM (LR)) among all samples. Peptides could be quantified in the digested unmalted quinoa **C** at the following concentrations: 0.17 μM (HI), 0.40 μM (IR), 0.86 μM (HL), 1.16 μM. (LR), 4.34 μM (APF), 13.64 μM (RL), and 16.07 μM (RI). Comparing the average values of the digested malted samples with the digested but unmalted quinoa, it was revealed that the malted and digested samples had a higher peptide concentration (Tables 3 and 4). A statistical analysis of the peptide concentrations was performed to identify the most influential malting parameter (temperature, moisture, time). This showed varying dependencies on the malting conditions. The overall concentration of all seven peptides in the undigested malts was dependent on the germination time, the product of time and temperature, and the temperature squared (*p* < 0.05), having its optimum at a high moisture and long germination time (Figure 7). After *in vitro* simulated gastrointestinal digest, however, the main parameters influencing the peptide concentrations were time, the product of humidity and temperature, and the squared humidity (*p* < 0.1, Figure 8). These results differ from the ones obtained after the inhibition measurement described in Section 3.1. There are various reasons behind that, for example, there might be more DPP-IV inhibitory peptides in the samples that have not yet been described in the literature and need to be identified in further studies. Additionally, not all IC_50_ values of the measured peptides are known, making it impossible to draw direct conclusions from the peptide concentrations to the inhibitory effect the samples might have. Nevertheless, it can be concluded that malting positively affects the formation of bioactive peptides. In the buffer control **B**, in which the enzymes pepsin, trypsin, and chymotrypsin were incubated without the addition of quinoa malt, small amounts of DPP-IV inhibitory peptides could be detected (Table 4), which could be explained by the fact that the proteins partially digested themselves. According to the protein sequences listed on UniProt (https://www.uniprot.org, protein sequences given in the Supporting Information, Table S9), the following DPP-IV inhibitory peptides, APF, IR, LR (pepsin); RL (trypsin); and LR, RL (chymotrypsin), could be released by partial self-digestion. Since the concentrations were much lower compared to the digested quinoa malt samples, the results of the buffer control could be neglected.

Additionally, a correlation analysis was performed to investigate the connection between the DPP-IV inhibitory potential of the samples and the concentration of the different inhibitory peptides. In the undigested samples, a positive correlation between the measured inhibition and the concentrations of the peptides HI (*p* < 0.01) and HL (*p* < 0.01) could be found, and after simulated gastrointestinal digest, only HL (*p* < 0.05) could be correlated significantly. This suggests that further unknown DPP-IV inhibitory peptides in the samples might influence the malts’ inhibitory potential.

Overall, the *in vitro* simulated gastrointestinal digest had a higher impact on the release of DPP-IV inhibitory peptides than the malting if regarded singularly. However, the combination of the malting and the following digest led to a very high concentration of inhibitory peptides as well as a high inhibitory potential, suggesting that during the malting, the storage proteins of quinoa are broken down into oligopeptides, which are then further degraded into the bioactive peptides during the digestion. These findings highlight the high potential of malting as a processing method of the pseudocereal.

### 3.4 Analysis of the Protein Size Distribution in the Malts

This degradation of proteins into (oligo-)peptides could also be shown using lab-on-a-chip technology. The purpose of this investigation was to compare the unmalted quinoa with the malts and the respective proteins therein since the proteinogenic origin of DPP-IV inhibitory peptides in cereals and pseudocereals is barely known. Tok et al. could show that in barley, most peptides inhibiting DPP-IV originated from globulins.^21^ For a first glance at the origin of the peptides in quinoa malt, the molecular weight distributions of the proteins in the quinoa malt, as well as in the unmalted grains, were investigated using lab-on-a-chip technology. The resulting relative protein concentrations (in ng/μL) were then grouped depending on the protein sizes, especially emphasizing the globulin and albumin fractions. According to Dakhili et al., globulins in quinoa mainly consist of two types: the 7S globulin, which can reach sizes of up to 60 kDa, and the 11S globulins, which are divided into 11AS with 30 kDa and 11BS with 20 kDa. Albumins have been shown to have sizes lower than 20 kDa.^52^ It has also been described that the majority of quinoa seed proteins are globulins and albumins, accounting for 37 and 35%, respectively, but only little to no prolamins (0.5–0.7%).^53^ Thus, in the present study, the malting-dependent variation of three protein size ranges grouped by molecular weight based on the literature was further investigated: ≤ 20 kDa, 20–30 kDa, and 50–60 kDa. Furthermore, the protein groups were correlated with the DPP-IV inhibition and the inhibitory peptide concentrations of the malt samples.

**Figure 9:**
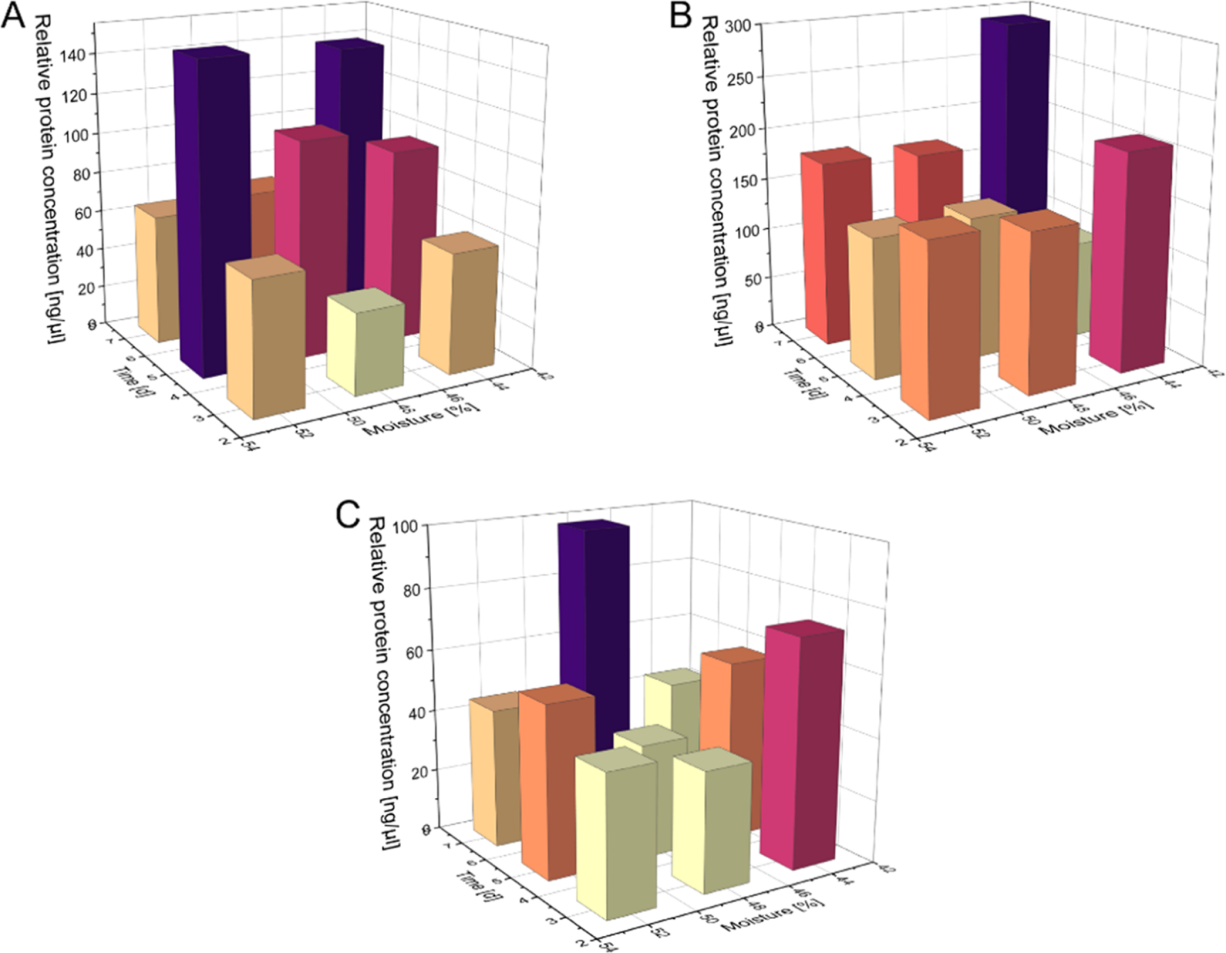
Bar chart of the measured relative protein concentrations by malts germinated at different moistures over varying time. The plot shows the data from the protein concentrations of the fraction below 20 kDa (A), 20–30 kDa (B), and 50–60 kDa (C).

Concerning the albumin-containing size fraction (<20 kDa), the lowest concentration (0 ng/μL) was found in quinoa malted for 7 days at 44% and 15 °C, while the highest concentration (150.8 ng/μL) was found in the samples malted for 5 days at 52% and 11.5 °C (Table S10, visualized in Figure 9A). The relative protein concentration at this size group in the unmalted grains was 56.3 ng/μL. This suggests that the proteins with higher molecular masses were degraded at high moisture, resulting in oligopeptides below 20 kDa, leading to a higher concentration in this fraction than in the unmalted pseudocereal. At low moistures, however, a strong degradation of small proteins occurred, while the degradation of higher molecular weight proteins was less effective. The statistical analysis of this group also shows that the squared time and the moisture are the malting parameters with the highest impact on the protein concentration in this range. Since time as a single parameter only has a low effect, the relative impact of the squared time is lowered. Looking at the subunits of the 11S globulin, between 20–30 kDa, the highest degradation appears to have occurred at 44% moisture and 11.5 °C for 5 days (Table S10 and Figure 9B), resulting in the minimal relative protein concentration of 92.9 ng/μL in this malt. The highest concentration (435.60 ng/μL) in this size fraction can be found in the quinoa malted at 44%, 7 days, and 8 °C, while the unmalted quinoa has a relative protein concentration of 266.3 ng/μL in this range. The statistical analysis highlights the squared temperature and the temperature as the significant factors (*p* < 0.1), followed by the time. Thus, the medium temperature and medium time chosen for the RSM model seem to be the optimal conditions for a maximum degradation of proteins between 20–30 kDa, while at longer durations and lower temperatures, the opposite is favored. At the range of 50–60 kDa, the lowest relative protein concentration (10.60 ng/μL) can again be found in the quinoa sample malted at 44%, 15 °C, and 7 days (Table S10 and Figure 9C), while the highest concentration (98.10 ng/μL) was found in sample malted at 48%, 7 days, and 11.5 °C. The relative concentration in the unmalted sample was 64.4 ng/μL. Thus, these proteins were degraded in all samples during malting. It can be noted that high temperature combined with low moisture seems to be advantageous for a high level of storage protein degradation. However, the statistical analysis did not reveal any significant parameters for achieving a high protein degradation in this range, suggesting a similar level of protein hydrolysis in all samples. The surface plots of all three protein size ranges and temperatures are displayed in Figure S1.

Subsequently, the correlation between these relative protein concentrations and the inhibition or the concentrations of inhibitory peptides in the undigested samples was investigated. However, no significant correlation (*p* < 0.1) could be found between the relative concentration of proteins in the different kDa ranges and the inhibitory potentials of the malts (Table S11). Despite the high p-values, the correlation coefficients indicate a possible origin of the peptides in all three protein size groups. Positive correlations could be seen when investigating the relationship between the relative protein concentrations and the concentrations of the different DPP-IV inhibitory peptides. Namely, the protein fraction below 20 kDa positively correlated with the concentrations of RL (*p* < 0.01) and LR (*p* < 0.1), whereas for the higher molecular weight proteins, more negative correlations can be observed (Table S11). This indicates that the proteins of higher molecular weight are degraded into oligopeptides (<20 kDa) and the bioactive peptides investigated here. Additionally, other inhibitory peptides not described in the literature might also be present in the samples and influence the inhibitory effect. Further studies will be conducted to clarify the origin of the DPP-IV inhibitory peptides in quinoa. For example, precursor peptides that occur in the malt and are subsequently degraded into bioactive peptides could be investigated, and their sequences could be aligned with those of known storage proteins.

In summary, malting has a significant influence on the DPP-IV inhibitory potential of quinoa, making it a valuable raw material for foods and beverages for patients suffering from type 2 diabetes mellitus. Especially after simulated gastrointestinal digest, the inhibitory potential of most malts could be further increased. This suggests that quinoa malt is a putative source of DPP-IV inhibitory peptides after ingestion. In addition, an accurate and robust UHPLC-MS/MS method was developed for the quantitation of literature-known DPP-IV inhibitory peptides, highlighting that the seven peptides APF, HI, HR, IR, LR, RI, and RL were present in quinoa malt. The application of this method revealed the highest concentration of these peptides in the digest of another malt (7 days, 44%, and 15 °C) than the one causing the highest DPP-IV inhibitory potential. This hints toward the presence of further unknown inhibitory peptides in the samples. The globulins and albumins could furthermore be identified as putative origins of these peptides. The focus of this study was on the enrichment and quantification of DPP-IV inhibitory peptides in quinoa using a systematic malting study. Future research will build on this study and identify further inhibitory peptides, determine their IC_50_, and investigate the bioavailability. This study shows the high influence of malting and subsequent gastrointestinal digest on the release of literature-known DPP-IV inhibitory peptides from quinoa.

## Supporting Information Available

Literature-known DPP-IV inhibitory peptides used for the targeted anaylsis and their origins; MS/MS parameters calculated in silico (using Skyline) for the screening of literature known DPP-IV inhibitory peptides; MS/MS parameters used for the quantification of candidate peptides; validation experiments for quantitation of identified peptides in quinoa malt; formulas calculated through stepwise regression analysis and used for the design of surface plots. *T* = temperature, *t* = time, *m* = moisture during germination. All other surface plots were made from the measured data; inhibitory potential of the quinoa malts when related to the original protein concentration of the extract; concentrations of quantified peptides (APF, HI, HL, RI, RL, IR, and LR), in μmol/L in undigested samples (1–25, A = unmalted quinoa); concentrations of quantified peptides (APF, HI, HL, RI, RL, IR, and LR), in μmol/L in digested samples (baskets 1-25, B = unmalted quinoa, C = enzyme control); enzymes used for the simulated gastrointestinal digest and their protein sequences from https://www.uniprot.org; relative protein concentrations of the quinoa malts and the unmalted grains at different protein size groups; correlation of the relative protein concentrations at different protein fractions and the concentration of DPP-IV inhibitory peptides. The correlation coefficient of the Pearson correlation is given as well the *p*-value as an indicator of significance;Surface plot of the measured relative protein concentrations of malts germinated at different moisture over time. The plot shows the data from the protein concentrations of the fraction below 20 kDa from malts germinated at 8 °C (A), 11.5 °C (B), or 15 °C (C), the protein size fraction 20–30 kDa from malts germinated at 8 °C (D), 11.5 °C (E), or 15 °C (F), and the protein size fraction 50–60 kDa from malts germinated at 8 °C (G), 11.5 °C (H), or 15 °C (I) (PDF)

## Abbreviations Used

DPP-IV: dipeptidyl peptidase IV
UHPLC-MS/MS: ultra-high-performance liquid chromatography-tandem mass spectrometry
LoD: limit of detection
LoQ: limit of quantification
ESI: electrospray ionization
MRM: multiple reaction monitoring mode
RSM: response surface methodology
