# Supplementary material for: Enrichment and Quantitation of Dipeptidyl Peptidase IV Inhibitory Peptides in Quinoa upon Systematic Malting
Source: J Agric Food Chem. 2024 May 11;72(20):11480–92. doi: 10.1021/acs.jafc.4c00570 (PMC11117401; doi:10.1021/acs.jafc.4c00570)
Supplement: Supplementary file 1 — jf4c00570_si_001.pdf [file jf4c00570_si_001.pdf]

Supporting Information

Enrichment and quantitation of dipeptidyl peptidase IV inhibitory peptides in quinoa upon systematic malting

Tabea D. U. Kröber [1,+], Magdalena Holzer [2,+], Roland Kerpes [1,*],

Verena K. Mittermeier-Kleßinger [2], Corinna Dawid [2,3] and Thomas Becker [1]

[1] Chair of Brewing and Beverage Technology, School of Life Sciences Weihenstephan, Technical University of Munich, Weihenstephaner Steig 20, 85354 Freising, Germany

[2] Chair of Food Chemistry and Molecular Sensory Science, School of Life Sciences Weihenstephan, Technical University of Munich, Lise-Meitner-Strasse 34, 85354 Freising, Germany

[3] Professorship for Functional Phytometabolomics, School of Life Sciences Weihenstephan, Technical University of Munich, Lise-Meitner-Strasse 34, 85354 Freising, Germany

[*] Correspondence: roland.kerpes@tum.de

Table of contents

Table S 1……S3

Table S 2……S9

Table S 3……S129

Table S 4……S130

Table S 5……S131

Table S 6……S132

Table S 7……S133

Table S 8……S134

Table S 9……S135

Table S 10……S136

Table S11……S137

Figure S 1……S138

References……S139

Table S 1: Literature-known DPP-IV inhibitory peptides used for the targeted anaylsis and their origins.

Compound	Origin	References
AAATP	Pork	1
AL	Cow milk	2
APA	*In silico*	3
APF	*In silico*	3
APGPAGP	Soy bean	4
APT	*In silico*	3
CAYQWQRPVDRIR	Tuna	5
DPF	Yam root	6
EK	Cow milk	2
FF	*In silico*	7
FL	Cow milk	2
FLQP	Cow milk	2
FP	*In silico*	7
FPF	*In silico*	3
FPI	*In silico*	3
FPW	*In silico*	3
FSD	Barbel	8
GGPAGPAV	Salmon gelatin	9
GL	Cow milk	2
GP	*In silico*	7
GPA	*In silico*	3
GPAE	*In silico*	3
GPAG	Porcine skin gelatin	10
GPAV	*In silico*	3
GPG	*In silico*	3

GPGA	Atlantic salmon skin gelatin	[7]
GPVA	Salmon gelatin	[9]
HI	*In silico*	[11]
HL	Cow milk	[2]
HP	Cow milk	[12]
IAVPTGVA	Soy bean	[4]
IHI	*In silico*	[3]
ILDKEGIDY	Camel milk	[3]
ILDKEGIQY	Camel milk	[3]
ILDKVGINY	Camel milk	[3]
ILELA	Camel milk	[3]
IP	Cow milk	[12]
IPA	Cow milk	[13]
IPAVF	Cow milk	[14]
IPAVFK	Cow milk	[14]
IPF	*In silico*	[3]
IPI	Cow milk	[15]
IPIQY	Cow milk	[15]
IPM	Cow milk	[15]
IPPL	Cow milk	[12]
IPPLTQT	Cow milk	[12]
IPPLTQTPV	Cow milk	[14]
IPQVS	Rape seed	[16]
IPSK	Cow milk	[15]
IPW	*In silico*	[17]
IR	*In silico*	[11]
IVQNNDSTEYGLF	Cow milk	[18]

KL	*In silico*	[7]
KPA	*In silico*	[7]
KVEPLP	Antarctic krill	[19]
LAHKALCSEKL	Cow milk	[18]
LAHKPL	Camel milk	[3]
LAPSTM	*Ruditapes philippinarum*	[20]
LKPTPEGDL	Cow milk	[18]
LKPTPEGDLEIL	Cow milk	[18]
LLQLEAIR	Camel milk	[3]
LLQNIPPLT	Cow milk	[15]
LP	Cow milk	[15]
LPA	*In silico*	[3]
LPIIDI	Soy bean	[4]
LPL	Cow milk	[15]
LPLPL	Cow milk	[15]
LPLPLL	Cow milk	[15]
LPP	*In silico*	[3]
LPQ	Gouda cheese, wheat	[3, 21, 22]
LPQNIPPL	Gouda cheese	[21]
LPQNIPPLT	Cow milk	[15]
LPVP	Camel milk	[3]
LPVPQ	Cow milk	[15]
LPYPY	Cow milk	[15]
LQAFEPLR	Oat	[23]
LQP	*In silico*	[3]
LR	*In silico*	[7]
LTFPGSAED	Lupin seeds	[24]

LW	Gouda cheese	[21]
MP	Defatted rice bran	[25]
MPVQA	Camel milk	[3]
MW	Cow milk	[2]
NLGIILR	*In silico*	[3]
PACGGFWISGRPG	Tuna	[5]
PAGDDAPR	*In silico*	[3]
PAGDDAPRA	*In silico*	[3]
PAL	*In silico*	[3]
PGPIHNS	Cow milk	[15]
PGVGGPLGPIGPCYE	Tuna	[5]
PP	*In silico*	[7]
PPP	*In silico*	[11]
QPF	*In silico*	[3]
QPHQPLPPT	Cow milk	[12]
QPLPPT	Cow milk	[15]
QPQ	Wheat	[22] [3]
RI	*In silico*	[11]
RL	Yam root	[6]
RP	Cow milk	[15]
RRRW	Yam root	[6]
SL	Cow milk	[2]
SPQ	Wheat	[3, 22]
SPVVPF	Camel milk	[3]
TPEVDDEALEK	*In silico*	[3]
TPVVVPP	Cow milk	[12]
TQMVDEEIMEKFR	Mare milk	[26]

VA	Cow milk	[2]
VAGTWY	Cow milk	[27]
VC	*In silico*	[7]
VLGP	Cow milk	[2]
VLVLDTDYK	Cow milk	[18]
VP	Defatted rice bran	[25]
VPGEIVE	Cow milk	[15]
VPITPTL	Gouda cheese	[21]
VPITPZ	Gouda cheese	[21]
VPL	Boarfish	[28]
VR	*In silico*	[7]
WA	*In silico*	[7]
WC	Cow milk	[2]
WD	*In silico*	[7]
WE	*In silico*	[7]
WF	*In silico*	[7]
WG	*In silico*	[7]
WI	Cow milk	[2]
WIQP	Cow milk	[2]
WK	Cow milk	[2]
WL	Cow milk	[2]
WLAHKAL	Cow milk	[18]
WLAHKALCSEKLDQ	Cow milk	[18]
WM	Cow milk	[2]
WN	Cow milk	[2]
WP	Cow milk	[2]
WPF	*In silico*	[3]

WPFPGP	*In silico*	[3]
WPG	*In silico*	[3]
WPL	*In silico*	[3]
WPT	*In silico*	[3]
WPVEPF	*In silico*	[3]
WPW	*In silico*	[3]
WQ	Cow milk	[2]
WR	Cow milk	[2]
WRS	*In silico*	[11]
WS	Cow milk	[2]
WSG	*In silico*	[3]
WT	Cow milk	[2]
WV	Cow milk	[2]
WW	Cow milk	[2]
WY	Cow milk	[2]
YP	Cow milk	[15]
YPEPGPIPN	Gouda cheese	[21]
YPY	Cow milk	[15]
YPYY	*In silico*	[15]

Table S 2: MS/MS parameters calculated in silico *(using Skyline) for the screening of literature known DPP-IV inhibitory peptides.*

Common settings for all peptides: DP 80V, EP 10V, CXP 15V, Dwell time 5 ms

Q1	Q3	compound	CE
430.2	385.2	AAATP	20.1
430.2	359.2	AAATP	20.1
430.2	342.2	AAATP	20.1
430.2	332.2	AAATP	20.1
430.2	315.2	AAATP	20.1
430.2	314.1	AAATP	20.1
430.2	288.2	AAATP	20.1
430.2	287.2	AAATP	20.1
430.2	271.1	AAATP	20.1
430.2	243.1	AAATP	20.1
430.2	231.1	AAATP	20.1
430.2	217.1	AAATP	20.1
430.2	214.1	AAATP	20.1
430.2	200.1	AAATP	20.1
430.2	186.1	AAATP	20.1
430.2	160.1	AAATP	20.1
430.2	143.1	AAATP	20.1
430.2	142.1	AAATP	20.1
430.2	116.1	AAATP	20.1
430.2	115.1	AAATP	20.1
430.2	99.0	AAATP	20.1
430.2	89.1	AAATP	20.1
430.2	72.0	AAATP	20.1
203.1	158.1	AL	10.0
203.1	132.1	AL	10.0
203.1	115.1	AL	10.0

203.1	89.1	AL	10.0
203.1	72.0	AL	10.0
258.1	213.1	APA	11.6
258.1	187.1	APA	11.6
258.1	186.1	APA	11.6
258.1	170.1	APA	11.6
258.1	169.1	APA	11.6
258.1	141.1	APA	11.6
258.1	116.0	APA	11.6
258.1	90.1	APA	11.6
258.1	89.1	APA	11.6
258.1	73.0	APA	11.6
258.1	72.0	APA	11.6
334.2	289.1	APF	15.4
334.2	263.1	APF	15.4
334.2	246.1	APF	15.4
334.2	192.1	APF	15.4
334.2	186.1	APF	15.4
334.2	169.1	APF	15.4
334.2	166.1	APF	15.4
334.2	149.1	APF	15.4
334.2	141.1	APF	15.4
334.2	89.1	APF	15.4
334.2	72.0	APF	15.4
566.3	521.2	APGPAGP	26.7
566.3	495.3	APGPAGP	26.7
566.3	478.2	APGPAGP	26.7
566.3	468.3	APGPAGP	26.7
566.3	451.2	APGPAGP	26.7
566.3	424.2	APGPAGP	26.7

566.3	423.2	APGPAGP	26.7
566.3	411.2	APGPAGP	26.7
566.3	398.2	APGPAGP	26.7
566.3	394.2	APGPAGP	26.7
566.3	381.2	APGPAGP	26.7
566.3	367.2	APGPAGP	26.7
566.3	366.2	APGPAGP	26.7
566.3	341.2	APGPAGP	26.7
566.3	340.2	APGPAGP	26.7
566.3	324.2	APGPAGP	26.7
566.3	323.2	APGPAGP	26.7
566.3	295.2	APGPAGP	26.7
566.3	270.1	APGPAGP	26.7
566.3	244.1	APGPAGP	26.7
566.3	243.1	APGPAGP	26.7
566.3	227.1	APGPAGP	26.7
566.3	226.1	APGPAGP	26.7
566.3	199.1	APGPAGP	26.7
566.3	198.1	APGPAGP	26.7
566.3	186.1	APGPAGP	26.7
566.3	173.1	APGPAGP	26.7
566.3	169.1	APGPAGP	26.7
566.3	156.1	APGPAGP	26.7
566.3	142.1	APGPAGP	26.7
566.3	141.1	APGPAGP	26.7
566.3	116.1	APGPAGP	26.7
566.3	99.0	APGPAGP	26.7
566.3	89.1	APGPAGP	26.7
566.3	72.0	APGPAGP	26.7
288.2	243.1	APT	13.1

288.2	217.1	APT	13.1
288.2	200.1	APT	13.1
288.2	186.1	APT	13.1
288.2	169.1	APT	13.1
288.2	146.0	APT	13.1
288.2	141.1	APT	13.1
288.2	120.1	APT	13.1
288.2	103.0	APT	13.1
288.2	89.1	APT	13.1
288.2	72.0	APT	13.1
874.4	1206.6	CAYQWQRPVDRIR	41.8
874.4	1189.6	CAYQWQRPVDRIR	41.8
874.4	1161562.0	CAYQWQRPVDRIR	41.8
874.4	1107.5	CAYQWQRPVDRIR	41.8
874.4	1090.5	CAYQWQRPVDRIR	41.8
874.4	1065.6	CAYQWQRPVDRIR	41.8
874.4	1062.5	CAYQWQRPVDRIR	41.8
874.4	1010.5	CAYQWQRPVDRIR	41.8
874.4	1225.7	CAYQWQRPVDRIR	41.8
874.4	1208.7	CAYQWQRPVDRIR	41.8
874.4	1039.6	CAYQWQRPVDRIR	41.8
874.4	1022.6	CAYQWQRPVDRIR	41.8
874.4	993.4	CAYQWQRPVDRIR	41.8
874.4	965.4	CAYQWQRPVDRIR	41.8
874.4	937.5	CAYQWQRPVDRIR	41.8
874.4	911.6	CAYQWQRPVDRIR	41.8
874.4	894.5	CAYQWQRPVDRIR	41.8
874.4	854.4	CAYQWQRPVDRIR	41.8
874.4	837.3	CAYQWQRPVDRIR	41.8
874.4	809.3	CAYQWQRPVDRIR	41.8

874.4	781.4	CAYQWQRPVDRIR	41.8
874.4	755.5	CAYQWQRPVDRIR	41.8
874.4	738.4	CAYQWQRPVDRIR	41.8
874.4	726.3	CAYQWQRPVDRIR	41.8
874.4	709.3	CAYQWQRPVDRIR	41.8
874.4	684.4	CAYQWQRPVDRIR	41.8
874.4	681.3	CAYQWQRPVDRIR	41.8
874.4	658.4	CAYQWQRPVDRIR	41.8
874.4	641.4	CAYQWQRPVDRIR	41.8
874.4	585.3	CAYQWQRPVDRIR	41.8
874.4	559.3	CAYQWQRPVDRIR	41.8
874.4	542.3	CAYQWQRPVDRIR	41.8
874.4	540.2	CAYQWQRPVDRIR	41.8
874.4	523.2	CAYQWQRPVDRIR	41.8
874.4	495.2	CAYQWQRPVDRIR	41.8
874.4	470.3	CAYQWQRPVDRIR	41.8
874.4	444.3	CAYQWQRPVDRIR	41.8
874.4	427.3	CAYQWQRPVDRIR	41.8
874.4	412.2	CAYQWQRPVDRIR	41.8
874.4	395.1	CAYQWQRPVDRIR	41.8
874.4	367.1	CAYQWQRPVDRIR	41.8
874.4	314.2	CAYQWQRPVDRIR	41.8
874.4	288.2	CAYQWQRPVDRIR	41.8
874.4	271.2	CAYQWQRPVDRIR	41.8
874.4	249.1	CAYQWQRPVDRIR	41.8
874.4	232.1	CAYQWQRPVDRIR	41.8
874.4	204.1	CAYQWQRPVDRIR	41.8
874.4	201.1	CAYQWQRPVDRIR	41.8
874.4	178.1	CAYQWQRPVDRIR	41.8
874.4	175.1	CAYQWQRPVDRIR	41.8

874.4	161.0	CAYQWQRPVDRIR	41.8
874.4	158.1	CAYQWQRPVDRIR	41.8
874.4	133.0	CAYQWQRPVDRIR	41.8
378.2	289.1	DPF	17.5
378.2	263.1	DPF	17.5
378.2	246.1	DPF	17.5
378.2	230.1	DPF	17.5
378.2	213.1	DPF	17.5
378.2	192.1	DPF	17.5
378.2	185.1	DPF	17.5
378.2	166.1	DPF	17.5
378.2	149.1	DPF	17.5
378.2	133.1	DPF	17.5
378.2	116.0	DPF	17.5
378.2	88.0	DPF	17.5
276.2	173.1	EK	12.5
276.2	147.1	EK	12.5
276.2	147.1	EK	12.5
276.2	130.1	EK	12.5
276.2	130.1	EK	12.5
276.2	102.1	EK	12.5
313.2	192.1	FF	14.3
313.2	166.1	FF	14.3
313.2	165.1	FF	14.3
313.2	149.1	FF	14.3
313.2	148.1	FF	14.3
313.2	120.1	FF	14.3
279.2	165.1	FL	12.7
279.2	158.1	FL	12.7
279.2	148.1	FL	12.7

279.2	132.1	FL	12.7
279.2	120.1	FL	12.7
279.2	115.1	FL	12.7
504.3	406.2	FLQP	23.7
504.3	389.2	FLQP	23.7
504.3	383.2	FLQP	23.7
504.3	361.2	FLQP	23.7
504.3	357.2	FLQP	23.7
504.3	340.2	FLQP	23.7
504.3	278.2	FLQP	23.7
504.3	270.1	FLQP	23.7
504.3	261.2	FLQP	23.7
504.3	244.1	FLQP	23.7
504.3	233.2	FLQP	23.7
504.3	227.1	FLQP	23.7
504.3	165.1	FLQP	23.7
504.3	148.1	FLQP	23.7
504.3	142.1	FLQP	23.7
504.3	120.1	FLQP	23.7
504.3	116.1	FLQP	23.7
504.3	99.0	FLQP	23.7
263.1	165.1	FP	11.9
263.1	148.1	FP	11.9
263.1	142.1	FP	11.9
263.1	120.1	FP	11.9
263.1	116.1	FP	11.9
263.1	99.0	FP	11.9
410.2	289.1	FPF	19.1
205.6	289.1	FPF	10.0
410.2	263.1	FPF	19.1

205.6	263.1	FPF	10.0
410.2	262.2	FPF	19.1
205.6	262.2	FPF	10.0
410.2	246.1	FPF	19.1
205.6	246.1	FPF	10.0
410.2	245.1	FPF	19.1
205.6	245.1	FPF	10.0
410.2	217.1	FPF	19.1
205.6	217.1	FPF	10.0
410.2	192.1	FPF	19.1
205.6	192.1	FPF	10.0
410.2	166.1	FPF	19.1
205.6	166.1	FPF	10.0
410.2	165.1	FPF	19.1
205.6	165.1	FPF	10.0
410.2	149.1	FPF	19.1
205.6	149.1	FPF	10.0
410.2	148.1	FPF	19.1
205.6	148.1	FPF	10.0
410.2	120.1	FPF	19.1
205.6	120.1	FPF	10.0
376.2	262.2	FPI	17.4
376.2	255.1	FPI	17.4
376.2	245.1	FPI	17.4
376.2	229.2	FPI	17.4
376.2	217.1	FPI	17.4
376.2	212.1	FPI	17.4
376.2	165.1	FPI	17.4
376.2	158.1	FPI	17.4
376.2	148.1	FPI	17.4

376.2	132.1	FPI	17.4
376.2	120.1	FPI	17.4
376.2	115.1	FPI	17.4
449.2	328.1	FPW	21.0
449.2	302.2	FPW	21.0
449.2	285.1	FPW	21.0
449.2	262.2	FPW	21.0
449.2	245.1	FPW	21.0
449.2	231.1	FPW	21.0
449.2	217.1	FPW	21.0
449.2	205.1	FPW	21.0
449.2	188.1	FPW	21.0
449.2	165.1	FPW	21.0
449.2	148.1	FPW	21.0
449.2	120.1	FPW	21.0
368.1	252.1	FSD	17.0
368.1	247.1	FSD	17.0
368.1	235.1	FSD	17.0
368.1	221.1	FSD	17.0
368.1	207.1	FSD	17.0
368.1	204.1	FSD	17.0
368.1	165.1	FSD	17.0
368.1	160.0	FSD	17.0
368.1	148.1	FSD	17.0
368.1	134.0	FSD	17.0
368.1	120.1	FSD	17.0
368.1	117.0	FSD	17.0
625.3	568.3	GGPAGPAV	29.6
625.3	511.3	GGPAGPAV	29.6
625.3	508.3	GGPAGPAV	29.6

625.3	437.2	GGPAGPAV	29.6
625.3	414.2	GGPAGPAV	29.6
625.3	343.2	GGPAGPAV	29.6
625.3	340.2	GGPAGPAV	29.6
625.3	283.1	GGPAGPAV	29.6
189.1	158.1	GL	10.0
189.1	132.1	GL	10.0
189.1	115.1	GL	10.0
189.1	75.1	GL	10.0
189.1	58.0	GL	10.0
173.1	142.1	GP	10.0
173.1	116.1	GP	10.0
173.1	99.0	GP	10.0
173.1	75.1	GP	10.0
173.1	58.0	GP	10.0
244.1	213.1	GPA	11.0
244.1	187.1	GPA	11.0
244.1	172.1	GPA	11.0
244.1	170.1	GPA	11.0
244.1	155.1	GPA	11.0
244.1	127.1	GPA	11.0
244.1	116.0	GPA	11.0
244.1	90.1	GPA	11.0
244.1	75.1	GPA	11.0
244.1	73.0	GPA	11.0
244.1	58.0	GPA	11.0
373.2	342.1	GPAE	17.3
373.2	316.2	GPAE	17.3
373.2	299.1	GPAE	17.3
373.2	245.1	GPAE	17.3

373.2	243.1	GPAE	17.3
373.2	226.1	GPAE	17.3
373.2	219.1	GPAE	17.3
373.2	202.1	GPAE	17.3
373.2	198.1	GPAE	17.3
373.2	174.0	GPAE	17.3
373.2	172.1	GPAE	17.3
373.2	155.1	GPAE	17.3
373.2	148.1	GPAE	17.3
373.2	131.0	GPAE	17.3
373.2	127.1	GPAE	17.3
373.2	75.1	GPAE	17.3
373.2	58.0	GPAE	17.3
301.2	270.1	GPAG	13.8
301.2	244.1	GPAG	13.8
301.2	243.1	GPAG	13.8
301.2	227.1	GPAG	13.8
301.2	226.1	GPAG	13.8
301.2	198.1	GPAG	13.8
301.2	173.1	GPAG	13.8
301.2	172.1	GPAG	13.8
301.2	155.1	GPAG	13.8
301.2	147.1	GPAG	13.8
301.2	130.1	GPAG	13.8
301.2	127.1	GPAG	13.8
301.2	102.0	GPAG	13.8
301.2	76.0	GPAG	13.8
301.2	75.1	GPAG	13.8
301.2	59.0	GPAG	13.8
301.2	58.0	GPAG	13.8

343.2	312.2	GPAV	15.8
343.2	286.2	GPAV	15.8
343.2	269.2	GPAV	15.8
343.2	243.1	GPAV	15.8
343.2	226.1	GPAV	15.8
343.2	215.1	GPAV	15.8
343.2	198.1	GPAV	15.8
343.2	189.1	GPAV	15.8
343.2	172.1	GPAV	15.8
343.2	172.1	GPAV	15.8
343.2	155.1	GPAV	15.8
343.2	144.1	GPAV	15.8
343.2	127.1	GPAV	15.8
343.2	118.1	GPAV	15.8
343.2	101.1	GPAV	15.8
343.2	75.1	GPAV	15.8
343.2	58.0	GPAV	15.8
230.1	199.1	GPG	10.3
230.1	173.1	GPG	10.3
230.1	172.1	GPG	10.3
230.1	156.1	GPG	10.3
230.1	155.1	GPG	10.3
230.1	127.1	GPG	10.3
230.1	102.0	GPG	10.3
230.1	76.0	GPG	10.3
230.1	75.1	GPG	10.3
230.1	59.0	GPG	10.3
230.1	58.0	GPG	10.3
301.2	270.1	GPGA	13.8
301.2	244.1	GPGA	13.8

301.2	229.1	GPGA	13.8
301.2	227.1	GPGA	13.8
301.2	212.1	GPGA	13.8
301.2	184.1	GPGA	13.8
301.2	173.1	GPGA	13.8
301.2	172.1	GPGA	13.8
301.2	155.1	GPGA	13.8
301.2	147.1	GPGA	13.8
301.2	130.1	GPGA	13.8
301.2	127.1	GPGA	13.8
301.2	116.0	GPGA	13.8
301.2	90.1	GPGA	13.8
301.2	75.1	GPGA	13.8
301.2	73.0	GPGA	13.8
301.2	58.0	GPGA	13.8
343.2	312.2	GPVA	15.8
343.2	286.2	GPVA	15.8
343.2	271.2	GPVA	15.8
343.2	269.2	GPVA	15.8
343.2	254.2	GPVA	15.8
343.2	226.2	GPVA	15.8
343.2	215.1	GPVA	15.8
343.2	189.1	GPVA	15.8
343.2	172.1	GPVA	15.8
343.2	172.1	GPVA	15.8
343.2	155.1	GPVA	15.8
343.2	127.1	GPVA	15.8
343.2	116.0	GPVA	15.8
343.2	90.1	GPVA	15.8
343.2	75.1	GPVA	15.8

343.2	73.0	GPVA	15.8
343.2	58.0	GPVA	15.8
269.2	158.1	HL	12.2
269.2	155.1	HL	12.2
269.2	138.1	HL	12.2
269.2	132.1	HL	12.2
269.2	115.1	HL	12.2
269.2	110.1	HL	12.2
253.1	155.1	HP	11.4
253.1	142.1	HP	11.4
253.1	138.1	HP	11.4
253.1	116.1	HP	11.4
253.1	110.1	HP	11.4
253.1	99.0	HP	11.4
628.4	556.3	IAVPTGA	29.8
628.4	541.3	IAVPTGA	29.8
628.4	539.3	IAVPTGA	29.8
628.4	515.3	IAVPTGA	29.8
628.4	511.3	IAVPTGA	29.8
628.4	499.3	IAVPTGA	29.8
628.4	498.3	IAVPTGA	29.8
628.4	482.3	IAVPTGA	29.8
628.4	470.2	IAVPTGA	29.8
628.4	454.3	IAVPTGA	29.8
628.4	444.2	IAVPTGA	29.8
628.4	427.2	IAVPTGA	29.8
628.4	398.3	IAVPTGA	29.8
628.4	381.3	IAVPTGA	29.8
628.4	371.2	IAVPTGA	29.8
628.4	353.3	IAVPTGA	29.8

628.4	345.2	IAVPTGA	29.8
628.4	328.2	IAVPTGA	29.8
628.4	301.2	IAVPTGA	29.8
628.4	284.2	IAVPTGA	29.8
628.4	274.1	IAVPTGA	29.8
628.4	256.2	IAVPTGA	29.8
628.4	248.1	IAVPTGA	29.8
628.4	231.1	IAVPTGA	29.8
628.4	202.2	IAVPTGA	29.8
628.4	185.1	IAVPTGA	29.8
628.4	173.1	IAVPTGA	29.8
628.4	157.1	IAVPTGA	29.8
628.4	147.1	IAVPTGA	29.8
628.4	131.1	IAVPTGA	29.8
628.4	130.1	IAVPTGA	29.8
628.4	116.0	IAVPTGA	29.8
628.4	114.1	IAVPTGA	29.8
628.4	90.1	IAVPTGA	29.8
628.4	86.1	IAVPTGA	29.8
628.4	73.0	IAVPTGA	29.8
382.2	295.1	IHI	17.7
382.2	269.2	IHI	17.7
382.2	268.2	IHI	17.7
382.2	252.1	IHI	17.7
382.2	251.2	IHI	17.7
382.2	223.2	IHI	17.7
382.2	158.1	IHI	17.7
382.2	132.1	IHI	17.7
382.2	131.1	IHI	17.7
382.2	115.1	IHI	17.7

382.2	114.1	IHI	17.7
382.2	86.1	IHI	17.7
1065.5	978.4	ILDKEGIDY	51.2
1065.5	952.5	ILDKEGIDY	51.2
1065.5	935.4	ILDKEGIDY	51.2
1065.5	901.5	ILDKEGIDY	51.2
1065.5	884.5	ILDKEGIDY	51.2
1065.5	865.4	ILDKEGIDY	51.2
1065.5	856.5	ILDKEGIDY	51.2
1065.5	839.4	ILDKEGIDY	51.2
1065.5	822.4	ILDKEGIDY	51.2
1065.5	786.5	ILDKEGIDY	51.2
1065.5	769.4	ILDKEGIDY	51.2
1065.5	750.3	ILDKEGIDY	51.2
1065.5	741.5	ILDKEGIDY	51.2
1065.5	724.4	ILDKEGIDY	51.2
1065.5	707.3	ILDKEGIDY	51.2
1065.5	673.4	ILDKEGIDY	51.2
1065.5	656.4	ILDKEGIDY	51.2
1065.5	628.4	ILDKEGIDY	51.2
1065.5	622.2	ILDKEGIDY	51.2
1065.5	616.4	ILDKEGIDY	51.2
1065.5	599.3	ILDKEGIDY	51.2
1065.5	596.3	ILDKEGIDY	51.2
1065.5	579.2	ILDKEGIDY	51.2
1065.5	571.3	ILDKEGIDY	51.2
1065.5	493.2	ILDKEGIDY	51.2
1065.5	487.3	ILDKEGIDY	51.2
1065.5	470.3	ILDKEGIDY	51.2
1065.5	467.2	ILDKEGIDY	51.2

1065.5	450.2	ILDKEGIDY	51.2
1065.5	442.3	ILDKEGIDY	51.2
1065.5	436.2	ILDKEGIDY	51.2
1065.5	410.2	ILDKEGIDY	51.2
1065.5	393.2	ILDKEGIDY	51.2
1065.5	359.2	ILDKEGIDY	51.2
1065.5	342.2	ILDKEGIDY	51.2
1065.5	323.1	ILDKEGIDY	51.2
1065.5	314.2	ILDKEGIDY	51.2
1065.5	297.1	ILDKEGIDY	51.2
1065.5	280.1	ILDKEGIDY	51.2
1065.5	244.2	ILDKEGIDY	51.2
1065.5	227.2	ILDKEGIDY	51.2
1065.5	208.1	ILDKEGIDY	51.2
1065.5	199.2	ILDKEGIDY	51.2
1065.5	182.1	ILDKEGIDY	51.2
1065.5	165.1	ILDKEGIDY	51.2
1065.5	131.1	ILDKEGIDY	51.2
1065.5	114.1	ILDKEGIDY	51.2
1065.5	86.1	ILDKEGIDY	51.2
1078.6	991.5	ILDKEGIQY	51.9
1078.6	965.5	ILDKEGIQY	51.9
1078.6	948.5	ILDKEGIQY	51.9
1078.6	914.5	ILDKEGIQY	51.9
1078.6	897.5	ILDKEGIQY	51.9
1078.6	878.4	ILDKEGIQY	51.9
1078.6	869.5	ILDKEGIQY	51.9
1078.6	852.4	ILDKEGIQY	51.9
1078.6	835.4	ILDKEGIQY	51.9
1078.6	786.5	ILDKEGIQY	51.9

1078.6	769.4	ILDKEGIQY	51.9
1078.6	763.4	ILDKEGIQY	51.9
1078.6	741.5	ILDKEGIQY	51.9
1078.6	737.4	ILDKEGIQY	51.9
1078.6	720.4	ILDKEGIQY	51.9
1078.6	673.4	ILDKEGIQY	51.9
1078.6	656.4	ILDKEGIQY	51.9
1078.6	635.3	ILDKEGIQY	51.9
1078.6	628.4	ILDKEGIQY	51.9
1078.6	616.4	ILDKEGIQY	51.9
1078.6	609.3	ILDKEGIQY	51.9
1078.6	599.3	ILDKEGIQY	51.9
1078.6	592.3	ILDKEGIQY	51.9
1078.6	571.3	ILDKEGIQY	51.9
1078.6	506.2	ILDKEGIQY	51.9
1078.6	487.3	ILDKEGIQY	51.9
1078.6	480.2	ILDKEGIQY	51.9
1078.6	470.3	ILDKEGIQY	51.9
1078.6	463.2	ILDKEGIQY	51.9
1078.6	449.2	ILDKEGIQY	51.9
1078.6	442.3	ILDKEGIQY	51.9
1078.6	423.2	ILDKEGIQY	51.9
1078.6	406.2	ILDKEGIQY	51.9
1078.6	359.2	ILDKEGIQY	51.9
1078.6	342.2	ILDKEGIQY	51.9
1078.6	336.1	ILDKEGIQY	51.9
1078.6	314.2	ILDKEGIQY	51.9
1078.6	310.1	ILDKEGIQY	51.9
1078.6	293.1	ILDKEGIQY	51.9
1078.6	244.2	ILDKEGIQY	51.9

1078.6	227.2	ILDKEGIQY	51.9
1078.6	208.1	ILDKEGIQY	51.9
1078.6	199.2	ILDKEGIQY	51.9
1078.6	182.1	ILDKEGIQY	51.9
1078.6	165.1	ILDKEGIQY	51.9
1078.6	131.1	ILDKEGIQY	51.9
1078.6	114.1	ILDKEGIQY	51.9
1078.6	86.1	ILDKEGIQY	51.9
1034.6	947.5	ILDKVGINY	49.7
1034.6	921.5	ILDKVGINY	49.7
1034.6	904.5	ILDKVGINY	49.7
1034.6	870.5	ILDKVGINY	49.7
1034.6	853.5	ILDKVGINY	49.7
1034.6	834.4	ILDKVGINY	49.7
1034.6	825.5	ILDKVGINY	49.7
1034.6	808.4	ILDKVGINY	49.7
1034.6	791.4	ILDKVGINY	49.7
1034.6	756.5	ILDKVGINY	49.7
1034.6	739.5	ILDKVGINY	49.7
1034.6	719.4	ILDKVGINY	49.7
1034.6	711.5	ILDKVGINY	49.7
1034.6	693.4	ILDKVGINY	49.7
1034.6	676.4	ILDKVGINY	49.7
1034.6	643.4	ILDKVGINY	49.7
1034.6	626.4	ILDKVGINY	49.7
1034.6	598.4	ILDKVGINY	49.7
1034.6	591.3	ILDKVGINY	49.7
1034.6	586.4	ILDKVGINY	49.7
1034.6	569.4	ILDKVGINY	49.7
1034.6	565.3	ILDKVGINY	49.7

1034.6	548.3	ILDKVGINY	49.7
1034.6	541.4	ILDKVGINY	49.7
1034.6	492.2	ILDKVGINY	49.7
1034.6	487.3	ILDKVGINY	49.7
1034.6	470.3	ILDKVGINY	49.7
1034.6	466.2	ILDKVGINY	49.7
1034.6	449.2	ILDKVGINY	49.7
1034.6	442.3	ILDKVGINY	49.7
1034.6	435.2	ILDKVGINY	49.7
1034.6	409.2	ILDKVGINY	49.7
1034.6	392.2	ILDKVGINY	49.7
1034.6	359.2	ILDKVGINY	49.7
1034.6	342.2	ILDKVGINY	49.7
1034.6	322.1	ILDKVGINY	49.7
1034.6	314.2	ILDKVGINY	49.7
1034.6	296.1	ILDKVGINY	49.7
1034.6	279.1	ILDKVGINY	49.7
1034.6	244.2	ILDKVGINY	49.7
1034.6	227.2	ILDKVGINY	49.7
1034.6	208.1	ILDKVGINY	49.7
1034.6	199.2	ILDKVGINY	49.7
1034.6	182.1	ILDKVGINY	49.7
1034.6	165.1	ILDKVGINY	49.7
1034.6	131.1	ILDKVGINY	49.7
1034.6	114.1	ILDKVGINY	49.7
1034.6	86.1	ILDKVGINY	49.7
558.4	486.3	ILELA	26.4
558.4	471.2	ILELA	26.4
558.4	469.3	ILELA	26.4
558.4	445.3	ILELA	26.4

558.4	441.3	ILELA	26.4
558.4	428.2	ILELA	26.4
558.4	373.2	ILELA	26.4
558.4	358.2	ILELA	26.4
558.4	356.2	ILELA	26.4
558.4	332.2	ILELA	26.4
558.4	328.2	ILELA	26.4
558.4	315.2	ILELA	26.4
558.4	244.2	ILELA	26.4
558.4	229.1	ILELA	26.4
558.4	227.2	ILELA	26.4
558.4	203.1	ILELA	26.4
558.4	199.2	ILELA	26.4
558.4	186.1	ILELA	26.4
558.4	131.1	ILELA	26.4
558.4	116.0	ILELA	26.4
558.4	114.1	ILELA	26.4
558.4	90.1	ILELA	26.4
558.4	86.1	ILELA	26.4
558.4	73.0	ILELA	26.4
229.2	142.1	IP	10.2
229.2	131.1	IP	10.2
229.2	116.1	IP	10.2
229.2	114.1	IP	10.2
229.2	99.0	IP	10.2
229.2	86.1	IP	10.2
300.2	228.2	IPA	13.7
300.2	213.1	IPA	13.7
300.2	211.1	IPA	13.7
300.2	187.1	IPA	13.7

300.2	183.1	IPA	13.7
300.2	170.1	IPA	13.7
300.2	131.1	IPA	13.7
300.2	116.0	IPA	13.7
300.2	114.1	IPA	13.7
300.2	90.1	IPA	13.7
300.2	86.1	IPA	13.7
300.2	73.0	IPA	13.7
447.3	360.2	IPAF	20.9
447.3	334.2	IPAF	20.9
447.3	317.2	IPAF	20.9
447.3	299.2	IPAF	20.9
447.3	282.2	IPAF	20.9
447.3	263.1	IPAF	20.9
447.3	254.2	IPAF	20.9
447.3	237.1	IPAF	20.9
447.3	228.2	IPAF	20.9
447.3	220.1	IPAF	20.9
447.3	211.1	IPAF	20.9
447.3	192.1	IPAF	20.9
447.3	183.1	IPAF	20.9
447.3	166.1	IPAF	20.9
447.3	149.1	IPAF	20.9
447.3	131.1	IPAF	20.9
447.3	114.1	IPAF	20.9
447.3	86.1	IPAF	20.9
674.4	587.3	IPAVFK	32.0
674.4	561.3	IPAVFK	32.0
674.4	545.3	IPAVFK	32.0
674.4	544.3	IPAVFK	32.0

674.4	528.3	IPAVFK	32.0
674.4	500.3	IPAVFK	32.0
674.4	490.3	IPAVFK	32.0
674.4	464.3	IPAVFK	32.0
674.4	447.3	IPAVFK	32.0
674.4	419.2	IPAVFK	32.0
674.4	398.3	IPAVFK	32.0
674.4	393.3	IPAVFK	32.0
674.4	381.3	IPAVFK	32.0
674.4	376.2	IPAVFK	32.0
674.4	353.3	IPAVFK	32.0
674.4	320.2	IPAVFK	32.0
674.4	299.2	IPAVFK	32.0
674.4	294.2	IPAVFK	32.0
674.4	282.2	IPAVFK	32.0
674.4	277.2	IPAVFK	32.0
674.4	254.2	IPAVFK	32.0
674.4	228.2	IPAVFK	32.0
674.4	211.1	IPAVFK	32.0
674.4	183.1	IPAVFK	32.0
674.4	173.1	IPAVFK	32.0
674.4	147.1	IPAVFK	32.0
674.4	131.1	IPAVFK	32.0
674.4	130.1	IPAVFK	32.0
674.4	114.1	IPAVFK	32.0
674.4	86.1	IPAVFK	32.0
376.2	289.1	IPF	17.4
376.2	263.1	IPF	17.4
376.2	246.1	IPF	17.4
376.2	228.2	IPF	17.4

376.2	211.1	IPF	17.4
376.2	192.1	IPF	17.4
376.2	183.1	IPF	17.4
376.2	166.1	IPF	17.4
376.2	149.1	IPF	17.4
376.2	131.1	IPF	17.4
376.2	114.1	IPF	17.4
376.2	86.1	IPF	17.4
342.2	255.1	IPI	15.8
342.2	229.2	IPI	15.8
342.2	228.2	IPI	15.8
342.2	212.1	IPI	15.8
342.2	211.1	IPI	15.8
342.2	183.1	IPI	15.8
342.2	158.1	IPI	15.8
342.2	132.1	IPI	15.8
342.2	131.1	IPI	15.8
342.2	115.1	IPI	15.8
342.2	114.1	IPI	15.8
342.2	86.1	IPI	15.8
633.4	546.3	IPIQY	30.0
633.4	520.3	IPIQY	30.0
633.4	503.3	IPIQY	30.0
633.4	469.3	IPIQY	30.0
633.4	452.3	IPIQY	30.0
633.4	449.2	IPIQY	30.0
633.4	424.3	IPIQY	30.0
633.4	423.2	IPIQY	30.0
633.4	406.2	IPIQY	30.0
633.4	341.3	IPIQY	30.0

633.4	336.1	IPIQY	30.0
633.4	324.2	IPIQY	30.0
633.4	310.1	IPIQY	30.0
633.4	296.2	IPIQY	30.0
633.4	293.1	IPIQY	30.0
633.4	228.2	IPIQY	30.0
633.4	211.1	IPIQY	30.0
633.4	208.1	IPIQY	30.0
633.4	183.1	IPIQY	30.0
633.4	182.1	IPIQY	30.0
633.4	165.1	IPIQY	30.0
633.4	131.1	IPIQY	30.0
633.4	114.1	IPIQY	30.0
633.4	86.1	IPIQY	30.0
360.2	273.1	IPM	16.6
360.2	247.1	IPM	16.6
360.2	230.1	IPM	16.6
360.2	228.2	IPM	16.6
360.2	211.1	IPM	16.6
360.2	183.1	IPM	16.6
360.2	176.0	IPM	16.6
360.2	150.1	IPM	16.6
360.2	133.0	IPM	16.6
360.2	131.1	IPM	16.6
360.2	114.1	IPM	16.6
360.2	86.1	IPM	16.6
439.3	352.2	IPPL	20.5
439.3	326.2	IPPL	20.5
439.3	325.2	IPPL	20.5
439.3	309.2	IPPL	20.5

439.3	308.2	IPPL	20.5
439.3	280.2	IPPL	20.5
439.3	255.1	IPPL	20.5
439.3	229.2	IPPL	20.5
439.3	228.2	IPPL	20.5
439.3	212.1	IPPL	20.5
439.3	211.1	IPPL	20.5
439.3	183.1	IPPL	20.5
439.3	158.1	IPPL	20.5
439.3	132.1	IPPL	20.5
439.3	131.1	IPPL	20.5
439.3	115.1	IPPL	20.5
439.3	114.1	IPPL	20.5
439.3	86.1	IPPL	20.5
769.4	682.3	IPPLTQT	36.7
769.4	667.4	IPPLTQT	36.7
769.4	656.4	IPPLTQT	36.7
769.4	650.4	IPPLTQT	36.7
769.4	639.3	IPPLTQT	36.7
769.4	622.4	IPPLTQT	36.7
769.4	585.3	IPPLTQT	36.7
769.4	559.3	IPPLTQT	36.7
769.4	542.3	IPPLTQT	36.7
769.4	539.4	IPPLTQT	36.7
769.4	522.3	IPPLTQT	36.7
769.4	494.3	IPPLTQT	36.7
769.4	488.2	IPPLTQT	36.7
769.4	462.3	IPPLTQT	36.7
769.4	445.2	IPPLTQT	36.7
769.4	438.3	IPPLTQT	36.7

769.4	421.3	IPPLTQT	36.7
769.4	393.3	IPPLTQT	36.7
769.4	375.2	IPPLTQT	36.7
769.4	349.2	IPPLTQT	36.7
769.4	332.1	IPPLTQT	36.7
769.4	325.2	IPPLTQT	36.7
769.4	308.2	IPPLTQT	36.7
769.4	280.2	IPPLTQT	36.7
769.4	274.1	IPPLTQT	36.7
769.4	248.1	IPPLTQT	36.7
769.4	231.1	IPPLTQT	36.7
769.4	228.2	IPPLTQT	36.7
769.4	211.1	IPPLTQT	36.7
769.4	183.1	IPPLTQT	36.7
769.4	146.0	IPPLTQT	36.7
769.4	131.1	IPPLTQT	36.7
769.4	120.1	IPPLTQT	36.7
769.4	114.1	IPPLTQT	36.7
769.4	103.0	IPPLTQT	36.7
769.4	86.1	IPPLTQT	36.7
965.6	878.5	IPPLTQTPV	46.3
965.6	865.5	IPPLTQTPV	46.3
965.6	852.5	IPPLTQTPV	46.3
965.6	848.5	IPPLTQTPV	46.3
965.6	835.5	IPPLTQTPV	46.3
965.6	820.5	IPPLTQTPV	46.3
965.6	781.4	IPPLTQTPV	46.3
965.6	768.5	IPPLTQTPV	46.3
965.6	755.4	IPPLTQTPV	46.3
965.6	751.4	IPPLTQTPV	46.3

965.6	738.4	IPPLTQTPV	46.3
965.6	723.4	IPPLTQTPV	46.3
965.6	684.4	IPPLTQTPV	46.3
965.6	667.4	IPPLTQTPV	46.3
965.6	658.4	IPPLTQTPV	46.3
965.6	650.4	IPPLTQTPV	46.3
965.6	641.4	IPPLTQTPV	46.3
965.6	622.4	IPPLTQTPV	46.3
965.6	571.3	IPPLTQTPV	46.3
965.6	545.3	IPPLTQTPV	46.3
965.6	539.4	IPPLTQTPV	46.3
965.6	528.3	IPPLTQTPV	46.3
965.6	522.3	IPPLTQTPV	46.3
965.6	494.3	IPPLTQTPV	46.3
965.6	470.2	IPPLTQTPV	46.3
965.6	444.2	IPPLTQTPV	46.3
965.6	438.3	IPPLTQTPV	46.3
965.6	427.2	IPPLTQTPV	46.3
965.6	421.3	IPPLTQTPV	46.3
965.6	393.3	IPPLTQTPV	46.3
965.6	342.2	IPPLTQTPV	46.3
965.6	325.2	IPPLTQTPV	46.3
965.6	316.2	IPPLTQTPV	46.3
965.6	308.2	IPPLTQTPV	46.3
965.6	299.2	IPPLTQTPV	46.3
965.6	280.2	IPPLTQTPV	46.3
965.6	241.1	IPPLTQTPV	46.3
965.6	228.2	IPPLTQTPV	46.3
965.6	215.1	IPPLTQTPV	46.3
965.6	211.1	IPPLTQTPV	46.3

965.6	198.1	IPPLTQTPV	46.3
965.6	183.1	IPPLTQTPV	46.3
965.6	144.1	IPPLTQTPV	46.3
965.6	131.1	IPPLTQTPV	46.3
965.6	118.1	IPPLTQTPV	46.3
965.6	114.1	IPPLTQTPV	46.3
965.6	101.1	IPPLTQTPV	46.3
965.6	86.1	IPPLTQTPV	46.3
543.3	456.2	IPQVS	25.6
543.3	455.3	IPQVS	25.6
543.3	438.3	IPQVS	25.6
543.3	430.2	IPQVS	25.6
543.3	413.2	IPQVS	25.6
543.3	410.3	IPQVS	25.6
543.3	359.2	IPQVS	25.6
543.3	356.2	IPQVS	25.6
543.3	339.2	IPQVS	25.6
543.3	333.2	IPQVS	25.6
543.3	316.2	IPQVS	25.6
543.3	311.2	IPQVS	25.6
543.3	231.1	IPQVS	25.6
543.3	228.2	IPQVS	25.6
543.3	211.1	IPQVS	25.6
543.3	205.1	IPQVS	25.6
543.3	188.1	IPQVS	25.6
543.3	183.1	IPQVS	25.6
543.3	132.0	IPQVS	25.6
543.3	131.1	IPQVS	25.6
543.3	114.1	IPQVS	25.6
543.3	106.1	IPQVS	25.6

543.3	89.0	IPQVS	25.6
543.3	86.1	IPQVS	25.6
444.3	357.2	IPSK	20.8
444.3	357.2	IPSK	20.8
444.3	331.2	IPSK	20.8
444.3	331.2	IPSK	20.8
444.3	315.2	IPSK	20.8
444.3	315.2	IPSK	20.8
444.3	314.2	IPSK	20.8
444.3	314.2	IPSK	20.8
444.3	298.2	IPSK	20.8
444.3	298.2	IPSK	20.8
444.3	270.2	IPSK	20.8
444.3	270.2	IPSK	20.8
444.3	260.1	IPSK	20.8
444.3	260.1	IPSK	20.8
444.3	234.1	IPSK	20.8
444.3	234.1	IPSK	20.8
444.3	228.2	IPSK	20.8
444.3	228.2	IPSK	20.8
444.3	217.1	IPSK	20.8
444.3	217.1	IPSK	20.8
444.3	211.1	IPSK	20.8
444.3	211.1	IPSK	20.8
444.3	183.1	IPSK	20.8
444.3	183.1	IPSK	20.8
444.3	173.1	IPSK	20.8
444.3	173.1	IPSK	20.8
444.3	147.1	IPSK	20.8
444.3	147.1	IPSK	20.8

444.3	131.1	IPSK	20.8
444.3	131.1	IPSK	20.8
444.3	130.1	IPSK	20.8
444.3	130.1	IPSK	20.8
444.3	114.1	IPSK	20.8
444.3	114.1	IPSK	20.8
444.3	86.1	IPSK	20.8
444.3	86.1	IPSK	20.8
415.2	328.1	IPW	19.3
415.2	302.2	IPW	19.3
415.2	285.1	IPW	19.3
415.2	231.1	IPW	19.3
415.2	228.2	IPW	19.3
415.2	211.1	IPW	19.3
415.2	205.1	IPW	19.3
415.2	188.1	IPW	19.3
415.2	183.1	IPW	19.3
415.2	131.1	IPW	19.3
415.2	114.1	IPW	19.3
415.2	86.1	IPW	19.3
750.4	1238.6	IVQNNDSTEYGLF	35.8
750.4	1221538.0	IVQNNDSTEYGLF	35.8
750.4	1193.5	IVQNNDSTEYGLF	35.8
750.4	1185.5	IVQNNDSTEYGLF	35.8
750.4	1181543.0	IVQNNDSTEYGLF	35.8
750.4	1164.5	IVQNNDSTEYGLF	35.8
750.4	1142.5	IVQNNDSTEYGLF	35.8
750.4	1136.5	IVQNNDSTEYGLF	35.8
750.4	1071427.0	IVQNNDSTEYGLF	35.8
750.4	1045.4	IVQNNDSTEYGLF	35.8

750.4	1028.4	IVQNNDSTEYGLF	35.8
750.4	1.001.453	IVQNNDSTEYGLF	35.8
750.4	1159.5	IVQNNDSTEYGLF	35.8
750.4	1018.5	IVQNNDSTEYGLF	35.8
750.4	973.5	IVQNNDSTEYGLF	35.8
750.4	957.4	IVQNNDSTEYGLF	35.8
750.4	931.4	IVQNNDSTEYGLF	35.8
750.4	914.4	IVQNNDSTEYGLF	35.8
750.4	889.4	IVQNNDSTEYGLF	35.8
750.4	872.4	IVQNNDSTEYGLF	35.8
750.4	844.4	IVQNNDSTEYGLF	35.8
750.4	842.4	IVQNNDSTEYGLF	35.8
750.4	816.4	IVQNNDSTEYGLF	35.8
750.4	799.4	IVQNNDSTEYGLF	35.8
750.4	788.4	IVQNNDSTEYGLF	35.8
750.4	771.4	IVQNNDSTEYGLF	35.8
750.4	755.3	IVQNNDSTEYGLF	35.8
750.4	743.4	IVQNNDSTEYGLF	35.8
750.4	729.3	IVQNNDSTEYGLF	35.8
750.4	712.3	IVQNNDSTEYGLF	35.8
750.4	701.4	IVQNNDSTEYGLF	35.8
750.4	684.3	IVQNNDSTEYGLF	35.8
750.4	656.3	IVQNNDSTEYGLF	35.8
750.4	654.3	IVQNNDSTEYGLF	35.8
750.4	628.3	IVQNNDSTEYGLF	35.8
750.4	611.3	IVQNNDSTEYGLF	35.8
750.4	586.3	IVQNNDSTEYGLF	35.8
750.4	569.3	IVQNNDSTEYGLF	35.8
750.4	541.3	IVQNNDSTEYGLF	35.8
750.4	525.2	IVQNNDSTEYGLF	35.8

750.4	499.3	IVQNNDSTEYGLF	35.8
750.4	482.2	IVQNNDSTEYGLF	35.8
750.4	472.3	IVQNNDSTEYGLF	35.8
750.4	455.3	IVQNNDSTEYGLF	35.8
750.4	427.3	IVQNNDSTEYGLF	35.8
750.4	362.2	IVQNNDSTEYGLF	35.8
750.4	358.2	IVQNNDSTEYGLF	35.8
750.4	341.2	IVQNNDSTEYGLF	35.8
750.4	336.2	IVQNNDSTEYGLF	35.8
750.4	319.2	IVQNNDSTEYGLF	35.8
750.4	313.2	IVQNNDSTEYGLF	35.8
750.4	305.2	IVQNNDSTEYGLF	35.8
750.4	279.2	IVQNNDSTEYGLF	35.8
750.4	262.1	IVQNNDSTEYGLF	35.8
750.4	230.2	IVQNNDSTEYGLF	35.8
750.4	213.2	IVQNNDSTEYGLF	35.8
750.4	192.1	IVQNNDSTEYGLF	35.8
750.4	185.2	IVQNNDSTEYGLF	35.8
750.4	166.1	IVQNNDSTEYGLF	35.8
750.4	149.1	IVQNNDSTEYGLF	35.8
750.4	131.1	IVQNNDSTEYGLF	35.8
750.4	114.1	IVQNNDSTEYGLF	35.8
750.4	86.1	IVQNNDSTEYGLF	35.8
260.2	158.1	KL	11.7
260.2	146.1	KL	11.7
260.2	129.1	KL	11.7
260.2	115.1	KL	11.7
260.2	101.1	KL	11.7
260.2	132.1	KL	11.7
315.2	243.2	KPA	14.4

315.2	226.2	KPA	14.4
315.2	213.1	KPA	14.4
315.2	198.2	KPA	14.4
315.2	187.1	KPA	14.4
315.2	170.1	KPA	14.4
315.2	146.1	KPA	14.4
315.2	129.1	KPA	14.4
315.2	116.0	KPA	14.4
315.2	101.1	KPA	14.4
315.2	90.1	KPA	14.4
315.2	73.0	KPA	14.4
700.4	598.3	KRIHF	33.3
700.4	572.3	KRIHF	33.3
700.4	555.3	KRIHF	33.3
700.4	552.4	KRIHF	33.3
700.4	535.3	KRIHF	33.3
700.4	507.4	KRIHF	33.3
700.4	442.2	KRIHF	33.3
700.4	416.2	KRIHF	33.3
700.4	415.3	KRIHF	33.3
700.4	399.2	KRIHF	33.3
700.4	398.3	KRIHF	33.3
700.4	370.3	KRIHF	33.3
700.4	329.1	KRIHF	33.3
700.4	303.1	KRIHF	33.3
700.4	302.2	KRIHF	33.3
700.4	286.1	KRIHF	33.3
700.4	285.2	KRIHF	33.3
700.4	257.2	KRIHF	33.3
700.4	192.1	KRIHF	33.3

700.4	166.1	KRIHF	33.3
700.4	149.1	KRIHF	33.3
700.4	146.1	KRIHF	33.3
700.4	129.1	KRIHF	33.3
700.4	101.1	KRIHF	33.3
682.4	584.4	KVEPLP	32.4
682.4	580.3	KVEPLP	32.4
682.4	567.4	KVEPLP	32.4
682.4	554.3	KVEPLP	32.4
682.4	539.4	KVEPLP	32.4
682.4	537.3	KVEPLP	32.4
682.4	481.2	KVEPLP	32.4
682.4	471.3	KVEPLP	32.4
682.4	455.3	KVEPLP	32.4
682.4	454.3	KVEPLP	32.4
682.4	438.2	KVEPLP	32.4
682.4	426.3	KVEPLP	32.4
682.4	374.2	KVEPLP	32.4
682.4	357.2	KVEPLP	32.4
682.4	352.2	KVEPLP	32.4
682.4	329.2	KVEPLP	32.4
682.4	326.2	KVEPLP	32.4
682.4	309.2	KVEPLP	32.4
682.4	255.1	KVEPLP	32.4
682.4	245.2	KVEPLP	32.4
682.4	229.2	KVEPLP	32.4
682.4	228.2	KVEPLP	32.4
682.4	212.1	KVEPLP	32.4
682.4	200.2	KVEPLP	32.4
682.4	146.1	KVEPLP	32.4

682.4	142.1	KVEPLP	32.4
682.4	129.1	KVEPLP	32.4
682.4	116.1	KVEPLP	32.4
682.4	101.1	KVEPLP	32.4
682.4	99.0	KVEPLP	32.4
635.4	1182.6	LAHKALC[CAM]SEKL	30.1
635.4	1156.6	LAHKALC[CAM]SEKL	30.1
635.4	1139.6	LAHKALC[CAM]SEKL	30.1
635.4	1138.6	LAHKALC[CAM]SEKL	30.1
635.4	1111556.0	LAHKALC[CAM]SEKL	30.1
635.4	1110.6	LAHKALC[CAM]SEKL	30.1
635.4	1085.6	LAHKALC[CAM]SEKL	30.1
635.4	1068.6	LAHKALC[CAM]SEKL	30.1
635.4	1027.5	LAHKALC[CAM]SEKL	30.1
635.4	1010.5	LAHKALC[CAM]SEKL	30.1
635.4	1155.6	LAHKALC[CAM]SEKL	30.1
635.4	982.5	LAHKALC[CAM]SEKL	30.1
635.4	974.5	LAHKALC[CAM]SEKL	30.1
635.4	948.5	LAHKALC[CAM]SEKL	30.1
635.4	931.5	LAHKALC[CAM]SEKL	30.1
635.4	898.5	LAHKALC[CAM]SEKL	30.1
635.4	881.5	LAHKALC[CAM]SEKL	30.1
635.4	853.5	LAHKALC[CAM]SEKL	30.1
635.4	846.4	LAHKALC[CAM]SEKL	30.1
635.4	820.4	LAHKALC[CAM]SEKL	30.1
635.4	811.5	LAHKALC[CAM]SEKL	30.1
635.4	803.4	LAHKALC[CAM]SEKL	30.1
635.4	794.4	LAHKALC[CAM]SEKL	30.1
635.4	775.4	LAHKALC[CAM]SEKL	30.1
635.4	766.4	LAHKALC[CAM]SEKL	30.1

635.4	749.4	LAHKALC[CAM]SEKL	30.1
635.4	732.4	LAHKALC[CAM]SEKL	30.1
635.4	662.3	LAHKALC[CAM]SEKL	30.1
635.4	651.4	LAHKALC[CAM]SEKL	30.1
635.4	636.3	LAHKALC[CAM]SEKL	30.1
635.4	634.4	LAHKALC[CAM]SEKL	30.1
635.4	619.3	LAHKALC[CAM]SEKL	30.1
635.4	606.4	LAHKALC[CAM]SEKL	30.1
635.4	538.3	LAHKALC[CAM]SEKL	30.1
635.4	521.3	LAHKALC[CAM]SEKL	30.1
635.4	502.3	LAHKALC[CAM]SEKL	30.1
635.4	493.3	LAHKALC[CAM]SEKL	30.1
635.4	476.3	LAHKALC[CAM]SEKL	30.1
635.4	467.3	LAHKALC[CAM]SEKL	30.1
635.4	459.2	LAHKALC[CAM]SEKL	30.1
635.4	450.3	LAHKALC[CAM]SEKL	30.1
635.4	422.3	LAHKALC[CAM]SEKL	30.1
635.4	415.2	LAHKALC[CAM]SEKL	30.1
635.4	389.2	LAHKALC[CAM]SEKL	30.1
635.4	372.2	LAHKALC[CAM]SEKL	30.1
635.4	339.2	LAHKALC[CAM]SEKL	30.1
635.4	322.2	LAHKALC[CAM]SEKL	30.1
635.4	294.2	LAHKALC[CAM]SEKL	30.1
635.4	286.2	LAHKALC[CAM]SEKL	30.1
635.4	260.2	LAHKALC[CAM]SEKL	30.1
635.4	243.2	LAHKALC[CAM]SEKL	30.1
635.4	202.2	LAHKALC[CAM]SEKL	30.1
635.4	185.1	LAHKALC[CAM]SEKL	30.1
635.4	158.1	LAHKALC[CAM]SEKL	30.1
635.4	157.1	LAHKALC[CAM]SEKL	30.1

635.4	132.1	LAHKALC[CAM]SEKL	30.1
635.4	131.1	LAHKALC[CAM]SEKL	30.1
635.4	115.1	LAHKALC[CAM]SEKL	30.1
635.4	114.1	LAHKALC[CAM]SEKL	30.1
635.4	86.1	LAHKALC[CAM]SEKL	30.1
678.4	591.3	LAHKPL	32.2
678.4	565.3	LAHKPL	32.2
678.4	564.4	LAHKPL	32.2
678.4	548.3	LAHKPL	32.2
678.4	547.3	LAHKPL	32.2
678.4	520.3	LAHKPL	32.2
678.4	519.3	LAHKPL	32.2
678.4	494.3	LAHKPL	32.2
678.4	477.3	LAHKPL	32.2
678.4	467.3	LAHKPL	32.2
678.4	450.3	LAHKPL	32.2
678.4	422.3	LAHKPL	32.2
678.4	383.2	LAHKPL	32.2
678.4	357.3	LAHKPL	32.2
678.4	340.2	LAHKPL	32.2
678.4	339.2	LAHKPL	32.2
678.4	322.2	LAHKPL	32.2
678.4	294.2	LAHKPL	32.2
678.4	255.1	LAHKPL	32.2
678.4	229.2	LAHKPL	32.2
678.4	212.1	LAHKPL	32.2
678.4	202.2	LAHKPL	32.2
678.4	185.1	LAHKPL	32.2
678.4	158.1	LAHKPL	32.2
678.4	157.1	LAHKPL	32.2

678.4	132.1	LAHKPL	32.2
678.4	131.1	LAHKPL	32.2
678.4	115.1	LAHKPL	32.2
678.4	114.1	LAHKPL	32.2
678.4	86.1	LAHKPL	32.2
619.3	532.2	LAPSTM	29.3
619.3	532.2	LAPSTM	29.3
619.3	506.2	LAPSTM	29.3
619.3	506.2	LAPSTM	29.3
619.3	489.2	LAPSTM	29.3
619.3	489.2	LAPSTM	29.3
619.3	487.3	LAPSTM	29.3
619.3	487.3	LAPSTM	29.3
619.3	470.3	LAPSTM	29.3
619.3	470.3	LAPSTM	29.3
619.3	461.2	LAPSTM	29.3
619.3	461.2	LAPSTM	29.3
619.3	442.3	LAPSTM	29.3
619.3	442.3	LAPSTM	29.3
619.3	435.2	LAPSTM	29.3
619.3	435.2	LAPSTM	29.3
619.3	418.2	LAPSTM	29.3
619.3	418.2	LAPSTM	29.3
619.3	386.2	LAPSTM	29.3
619.3	386.2	LAPSTM	29.3
619.3	369.2	LAPSTM	29.3
619.3	369.2	LAPSTM	29.3
619.3	364.1	LAPSTM	29.3
619.3	364.1	LAPSTM	29.3
619.3	341.2	LAPSTM	29.3

619.3	341.2	LAPSTM	29.3
619.3	338.1	LAPSTM	29.3
619.3	338.1	LAPSTM	29.3
619.3	321.1	LAPSTM	29.3
619.3	321.1	LAPSTM	29.3
619.3	299.2	LAPSTM	29.3
619.3	299.2	LAPSTM	29.3
619.3	282.2	LAPSTM	29.3
619.3	282.2	LAPSTM	29.3
619.3	277.1	LAPSTM	29.3
619.3	277.1	LAPSTM	29.3
619.3	254.2	LAPSTM	29.3
619.3	254.2	LAPSTM	29.3
619.3	251.1	LAPSTM	29.3
619.3	251.1	LAPSTM	29.3
619.3	234.1	LAPSTM	29.3
619.3	234.1	LAPSTM	29.3
619.3	202.2	LAPSTM	29.3
619.3	202.2	LAPSTM	29.3
619.3	185.1	LAPSTM	29.3
619.3	185.1	LAPSTM	29.3
619.3	176.0	LAPSTM	29.3
619.3	176.0	LAPSTM	29.3
619.3	157.1	LAPSTM	29.3
619.3	157.1	LAPSTM	29.3
619.3	150.1	LAPSTM	29.3
619.3	150.1	LAPSTM	29.3
619.3	133.0	LAPSTM	29.3
619.3	133.0	LAPSTM	29.3
619.3	131.1	LAPSTM	29.3

619.3	131.1	LAPSTM	29.3
619.3	114.1	LAPSTM	29.3
619.3	114.1	LAPSTM	29.3
619.3	86.1	LAPSTM	29.3
619.3	86.1	LAPSTM	29.3
969.5	882.4	LKPTPEGDL	46.5
969.5	856.4	LKPTPEGDL	46.5
969.5	855.5	LKPTPEGDL	46.5
969.5	839.4	LKPTPEGDL	46.5
969.5	838.4	LKPTPEGDL	46.5
969.5	810.4	LKPTPEGDL	46.5
969.5	754.3	LKPTPEGDL	46.5
969.5	740.4	LKPTPEGDL	46.5
969.5	728.3	LKPTPEGDL	46.5
969.5	723.4	LKPTPEGDL	46.5
969.5	711.3	LKPTPEGDL	46.5
969.5	695.4	LKPTPEGDL	46.5
969.5	683.4	LKPTPEGDL	46.5
969.5	666.4	LKPTPEGDL	46.5
969.5	657.3	LKPTPEGDL	46.5
969.5	638.4	LKPTPEGDL	46.5
969.5	631.3	LKPTPEGDL	46.5
969.5	614.3	LKPTPEGDL	46.5
969.5	556.2	LKPTPEGDL	46.5
969.5	554.4	LKPTPEGDL	46.5
969.5	537.3	LKPTPEGDL	46.5
969.5	530.2	LKPTPEGDL	46.5
969.5	513.2	LKPTPEGDL	46.5
969.5	509.3	LKPTPEGDL	46.5
969.5	459.2	LKPTPEGDL	46.5

969.5	457.3	LKPTPEGDL	46.5
969.5	440.3	LKPTPEGDL	46.5
969.5	433.2	LKPTPEGDL	46.5
969.5	416.2	LKPTPEGDL	46.5
969.5	412.3	LKPTPEGDL	46.5
969.5	356.3	LKPTPEGDL	46.5
969.5	339.2	LKPTPEGDL	46.5
969.5	330.1	LKPTPEGDL	46.5
969.5	311.2	LKPTPEGDL	46.5
969.5	304.2	LKPTPEGDL	46.5
969.5	287.1	LKPTPEGDL	46.5
969.5	273.1	LKPTPEGDL	46.5
969.5	259.2	LKPTPEGDL	46.5
969.5	247.1	LKPTPEGDL	46.5
969.5	242.2	LKPTPEGDL	46.5
969.5	230.1	LKPTPEGDL	46.5
969.5	214.2	LKPTPEGDL	46.5
969.5	158.1	LKPTPEGDL	46.5
969.5	132.1	LKPTPEGDL	46.5
969.5	131.1	LKPTPEGDL	46.5
969.5	115.1	LKPTPEGDL	46.5
969.5	114.1	LKPTPEGDL	46.5
969.5	86.1	LKPTPEGDL	46.5
662.9	1211652.0	LKPTPEGDLEIL	31.5
662.9	1193.6	LKPTPEGDLEIL	31.5
662.9	1083.6	LKPTPEGDLEIL	31.5
662.9	1080.6	LKPTPEGDLEIL	31.5
662.9	986.5	LKPTPEGDLEIL	31.5
662.9	951.5	LKPTPEGDLEIL	31.5
662.9	885.5	LKPTPEGDLEIL	31.5

662.9	838.4	LKPTPEGDLEIL	31.5
662.9	788.4	LKPTPEGDLEIL	31.5
662.9	723.4	LKPTPEGDLEIL	31.5
662.9	666.4	LKPTPEGDLEIL	31.5
662.9	659.4	LKPTPEGDLEIL	31.5
662.9	602.3	LKPTPEGDLEIL	31.5
662.9	537.3	LKPTPEGDLEIL	31.5
662.9	487.3	LKPTPEGDLEIL	31.5
662.9	440.3	LKPTPEGDLEIL	31.5
955.6	842.5	LLQLEAIR	45.8
955.6	781.5	LLQLEAIR	45.8
955.6	729.4	LLQLEAIR	45.8
955.6	668.4	LLQLEAIR	45.8
955.6	601.4	LLQLEAIR	45.8
955.6	597.4	LLQLEAIR	45.8
955.6	488.3	LLQLEAIR	45.8
955.6	468.3	LLQLEAIR	45.8
955.6	359.2	LLQLEAIR	45.8
955.6	355.2	LLQLEAIR	45.8
1008.6	895.5	LLQNIPPLT	48.4
1008.6	889.6	LLQNIPPLT	48.4
1008.6	782.4	LLQNIPPLT	48.4
1008.6	776.5	LLQNIPPLT	48.4
1008.6	679.4	LLQNIPPLT	48.4
1008.6	654.4	LLQNIPPLT	48.4
1008.6	582.4	LLQNIPPLT	48.4
1008.6	540.3	LLQNIPPLT	48.4
1008.6	469.3	LLQNIPPLT	48.4
1008.6	427.3	LLQNIPPLT	48.4
229.2	142.1	LP	10.2

229.2	131.1	LP	10.2
229.2	116.1	LP	10.2
229.2	114.1	LP	10.2
229.2	99.0	LP	10.2
229.2	86.1	LP	10.2
300.2	228.2	LPA	13.7
300.2	213.1	LPA	13.7
300.2	211.1	LPA	13.7
300.2	187.1	LPA	13.7
300.2	183.1	LPA	13.7
300.2	170.1	LPA	13.7
300.2	131.1	LPA	13.7
300.2	116.0	LPA	13.7
300.2	114.1	LPA	13.7
300.2	90.1	LPA	13.7
300.2	86.1	LPA	13.7
300.2	73.0	LPA	13.7
683.4	570.4	LPIIDI	32.5
683.4	552.3	LPIIDI	32.5
683.4	473.3	LPIIDI	32.5
683.4	437.3	LPIIDI	32.5
683.4	360.2	LPIIDI	32.5
683.4	324.2	LPIIDI	32.5
683.4	247.1	LPIIDI	32.5
683.4	211.1	LPIIDI	32.5
342.2	255.1	LPL	15.8
342.2	229.2	LPL	15.8
342.2	228.2	LPL	15.8
342.2	212.1	LPL	15.8
342.2	211.1	LPL	15.8

342.2	183.1	LPL	15.8
342.2	158.1	LPL	15.8
342.2	132.1	LPL	15.8
342.2	131.1	LPL	15.8
342.2	115.1	LPL	15.8
342.2	114.1	LPL	15.8
342.2	86.1	LPL	15.8
552.4	439.3	LPLPL	26.1
552.4	421.3	LPLPL	26.1
552.4	342.2	LPLPL	26.1
552.4	324.2	LPLPL	26.1
665.5	296.2	LPLPL	31.6
552.4	229.2	LPLPL	26.1
552.4	211.1	LPLPL	26.1
665.5	183.1	LPLPL	31.6
552.4	132.1	LPLPL	26.1
665.5	86.1	LPLPL	31.6
665.5	578.4	LPLPLL	31.6
665.5	552.4	LPLPLL	31.6
665.5	551.4	LPLPLL	31.6
665.5	535.3	LPLPLL	31.6
665.5	534.4	LPLPLL	31.6
665.5	506.4	LPLPLL	31.6
665.5	481.3	LPLPLL	31.6
665.5	455.3	LPLPLL	31.6
665.5	438.3	LPLPLL	31.6
665.5	438.3	LPLPLL	31.6
665.5	421.3	LPLPLL	31.6
665.5	393.3	LPLPLL	31.6
665.5	368.2	LPLPLL	31.6

665.5	342.2	LPLPLL	31.6
665.5	341.3	LPLPLL	31.6
665.5	325.2	LPLPLL	31.6
665.5	324.2	LPLPLL	31.6
665.5	271.2	LPLPLL	31.6
665.5	245.2	LPLPLL	31.6
665.5	228.2	LPLPLL	31.6
665.5	228.2	LPLPLL	31.6
665.5	211.1	LPLPLL	31.6
665.5	158.1	LPLPLL	31.6
665.5	132.1	LPLPLL	31.6
665.5	131.1	LPLPLL	31.6
665.5	115.1	LPLPLL	31.6
665.5	114.1	LPLPLL	31.6
326.2	239.1	LPP	15.0
163.6	239.1	LPP	10.0
326.2	228.2	LPP	15.0
163.6	228.2	LPP	10.0
326.2	213.1	LPP	15.0
163.6	213.1	LPP	10.0
326.2	211.1	LPP	15.0
163.6	211.1	LPP	10.0
326.2	196.1	LPP	15.0
163.6	196.1	LPP	10.0
326.2	183.1	LPP	15.0
163.6	183.1	LPP	10.0
326.2	142.1	LPP	15.0
163.6	142.1	LPP	10.0
326.2	131.1	LPP	15.0
163.6	131.1	LPP	10.0

326.2	116.1	LPP	15.0
163.6	116.1	LPP	10.0
326.2	114.1	LPP	15.0
163.6	114.1	LPP	10.0
326.2	99.0	LPP	15.0
163.6	99.0	LPP	10.0
326.2	86.1	LPP	15.0
163.6	86.1	LPP	10.0
357.2	270.1	LPQ	16.5
179.1	270.1	LPQ	10.0
357.2	244.1	LPQ	16.5
179.1	244.1	LPQ	10.0
357.2	228.2	LPQ	16.5
179.1	228.2	LPQ	10.0
357.2	227.1	LPQ	16.5
179.1	227.1	LPQ	10.0
357.2	211.1	LPQ	16.5
179.1	211.1	LPQ	10.0
357.2	183.1	LPQ	16.5
179.1	183.1	LPQ	10.0
357.2	173.1	LPQ	16.5
179.1	173.1	LPQ	10.0
357.2	147.1	LPQ	16.5
179.1	147.1	LPQ	10.0
357.2	131.1	LPQ	16.5
179.1	131.1	LPQ	10.0
357.2	130.1	LPQ	16.5
179.1	130.1	LPQ	10.0
357.2	114.1	LPQ	16.5
179.1	114.1	LPQ	10.0

357.2	86.1	LPQ	16.5
179.1	86.1	LPQ	10.0
891.5	804.4	LPQNIPPL	42.7
446.3	804.4	LPQNIPPL	20.9
891.5	778.4	LPQNIPPL	42.7
446.3	778.4	LPQNIPPL	20.9
891.5	777.5	LPQNIPPL	42.7
446.3	777.5	LPQNIPPL	20.9
891.5	761.4	LPQNIPPL	42.7
446.3	761.4	LPQNIPPL	20.9
891.5	760.4	LPQNIPPL	42.7
446.3	760.4	LPQNIPPL	20.9
891.5	732.4	LPQNIPPL	42.7
446.3	732.4	LPQNIPPL	20.9
891.5	707.4	LPQNIPPL	42.7
446.3	707.4	LPQNIPPL	20.9
891.5	681.4	LPQNIPPL	42.7
446.3	681.4	LPQNIPPL	20.9
891.5	680.4	LPQNIPPL	42.7
446.3	680.4	LPQNIPPL	20.9
891.5	664.4	LPQNIPPL	42.7
446.3	664.4	LPQNIPPL	20.9
891.5	663.4	LPQNIPPL	42.7
446.3	663.4	LPQNIPPL	20.9
891.5	635.4	LPQNIPPL	42.7
446.3	635.4	LPQNIPPL	20.9
891.5	583.4	LPQNIPPL	42.7
446.3	583.4	LPQNIPPL	20.9
891.5	579.3	LPQNIPPL	42.7
446.3	579.3	LPQNIPPL	20.9

891.5	566.3	LPQNIPPL	42.7
446.3	566.3	LPQNIPPL	20.9
891.5	553.3	LPQNIPPL	42.7
446.3	553.3	LPQNIPPL	20.9
891.5	538.3	LPQNIPPL	42.7
446.3	538.3	LPQNIPPL	20.9
891.5	536.3	LPQNIPPL	42.7
446.3	536.3	LPQNIPPL	20.9
891.5	470.3	LPQNIPPL	42.7
446.3	470.3	LPQNIPPL	20.9
891.5	465.3	LPQNIPPL	42.7
446.3	465.3	LPQNIPPL	20.9
891.5	453.2	LPQNIPPL	42.7
446.3	453.2	LPQNIPPL	20.9
891.5	439.3	LPQNIPPL	42.7
446.3	439.3	LPQNIPPL	20.9
891.5	425.3	LPQNIPPL	42.7
446.3	425.3	LPQNIPPL	20.9
891.5	422.3	LPQNIPPL	42.7
446.3	422.3	LPQNIPPL	20.9
891.5	356.2	LPQNIPPL	42.7
446.3	356.2	LPQNIPPL	20.9
891.5	352.2	LPQNIPPL	42.7
446.3	352.2	LPQNIPPL	20.9
891.5	339.2	LPQNIPPL	42.7
446.3	339.2	LPQNIPPL	20.9
891.5	326.2	LPQNIPPL	42.7
446.3	326.2	LPQNIPPL	20.9
891.5	311.2	LPQNIPPL	42.7
446.3	311.2	LPQNIPPL	20.9

891.5	309.2	LPQNIPPL	42.7
446.3	309.2	LPQNIPPL	20.9
891.5	255.1	LPQNIPPL	42.7
446.3	255.1	LPQNIPPL	20.9
891.5	229.2	LPQNIPPL	42.7
446.3	229.2	LPQNIPPL	20.9
891.5	228.2	LPQNIPPL	42.7
446.3	228.2	LPQNIPPL	20.9
891.5	212.1	LPQNIPPL	42.7
446.3	212.1	LPQNIPPL	20.9
891.5	211.1	LPQNIPPL	42.7
446.3	211.1	LPQNIPPL	20.9
891.5	183.1	LPQNIPPL	42.7
446.3	183.1	LPQNIPPL	20.9
891.5	158.1	LPQNIPPL	42.7
446.3	158.1	LPQNIPPL	20.9
891.5	132.1	LPQNIPPL	42.7
446.3	132.1	LPQNIPPL	20.9
891.5	131.1	LPQNIPPL	42.7
446.3	131.1	LPQNIPPL	20.9
891.5	115.1	LPQNIPPL	42.7
446.3	115.1	LPQNIPPL	20.9
891.5	114.1	LPQNIPPL	42.7
446.3	114.1	LPQNIPPL	20.9
891.5	86.1	LPQNIPPL	42.7
446.3	86.1	LPQNIPPL	20.9
992.6	905.5	LPQNIPPLT	47.6
496.8	905.5	LPQNIPPLT	23.3
992.6	890.5	LPQNIPPLT	47.6
496.8	890.5	LPQNIPPLT	23.3

992.6	879.5	LPQNIPPLT	47.6
496.8	879.5	LPQNIPPLT	23.3
992.6	873.5	LPQNIPPLT	47.6
496.8	873.5	LPQNIPPLT	23.3
992.6	862.5	LPQNIPPLT	47.6
496.8	862.5	LPQNIPPLT	23.3
992.6	845.5	LPQNIPPLT	47.6
496.8	845.5	LPQNIPPLT	23.3
992.6	808.4	LPQNIPPLT	47.6
496.8	808.4	LPQNIPPLT	23.3
992.6	782.4	LPQNIPPLT	47.6
496.8	782.4	LPQNIPPLT	23.3
992.6	777.5	LPQNIPPLT	47.6
496.8	777.5	LPQNIPPLT	23.3
992.6	765.4	LPQNIPPLT	47.6
496.8	765.4	LPQNIPPLT	23.3
992.6	760.4	LPQNIPPLT	47.6
496.8	760.4	LPQNIPPLT	23.3
992.6	732.4	LPQNIPPLT	47.6
496.8	732.4	LPQNIPPLT	23.3
992.6	680.4	LPQNIPPLT	47.6
496.8	680.4	LPQNIPPLT	23.3
992.6	680.4	LPQNIPPLT	47.6
496.8	680.4	LPQNIPPLT	23.3
992.6	663.4	LPQNIPPLT	47.6
496.8	663.4	LPQNIPPLT	23.3
992.6	654.4	LPQNIPPLT	47.6
496.8	654.4	LPQNIPPLT	23.3
992.6	637.4	LPQNIPPLT	47.6
496.8	637.4	LPQNIPPLT	23.3

992.6	635.4	LPQNIPPLT	47.6
496.8	635.4	LPQNIPPLT	23.3
992.6	583.4	LPQNIPPLT	47.6
496.8	583.4	LPQNIPPLT	23.3
992.6	566.3	LPQNIPPLT	47.6
496.8	566.3	LPQNIPPLT	23.3
992.6	566.3	LPQNIPPLT	47.6
496.8	566.3	LPQNIPPLT	23.3
992.6	540.3	LPQNIPPLT	47.6
496.8	540.3	LPQNIPPLT	23.3
992.6	538.3	LPQNIPPLT	47.6
496.8	538.3	LPQNIPPLT	23.3
992.6	523.3	LPQNIPPLT	47.6
496.8	523.3	LPQNIPPLT	23.3
992.6	470.3	LPQNIPPLT	47.6
496.8	470.3	LPQNIPPLT	23.3
992.6	453.2	LPQNIPPLT	47.6
496.8	453.2	LPQNIPPLT	23.3
992.6	453.2	LPQNIPPLT	47.6
496.8	453.2	LPQNIPPLT	23.3
992.6	427.3	LPQNIPPLT	47.6
496.8	427.3	LPQNIPPLT	23.3
992.6	425.3	LPQNIPPLT	47.6
496.8	425.3	LPQNIPPLT	23.3
992.6	410.2	LPQNIPPLT	47.6
496.8	410.2	LPQNIPPLT	23.3
992.6	356.2	LPQNIPPLT	47.6
496.8	356.2	LPQNIPPLT	23.3
992.6	356.2	LPQNIPPLT	47.6
496.8	356.2	LPQNIPPLT	23.3

992.6	339.2	LPQNIPPLT	47.6
496.8	339.2	LPQNIPPLT	23.3
992.6	330.2	LPQNIPPLT	47.6
496.8	330.2	LPQNIPPLT	23.3
992.6	313.2	LPQNIPPLT	47.6
496.8	313.2	LPQNIPPLT	23.3
992.6	311.2	LPQNIPPLT	47.6
496.8	311.2	LPQNIPPLT	23.3
992.6	259.1	LPQNIPPLT	47.6
496.8	259.1	LPQNIPPLT	23.3
992.6	233.2	LPQNIPPLT	47.6
496.8	233.2	LPQNIPPLT	23.3
992.6	228.2	LPQNIPPLT	47.6
496.8	228.2	LPQNIPPLT	23.3
992.6	216.1	LPQNIPPLT	47.6
496.8	216.1	LPQNIPPLT	23.3
992.6	211.1	LPQNIPPLT	47.6
496.8	211.1	LPQNIPPLT	23.3
992.6	183.1	LPQNIPPLT	47.6
496.8	183.1	LPQNIPPLT	23.3
992.6	146.0	LPQNIPPLT	47.6
496.8	146.0	LPQNIPPLT	23.3
992.6	131.1	LPQNIPPLT	47.6
496.8	131.1	LPQNIPPLT	23.3
992.6	120.1	LPQNIPPLT	47.6
496.8	120.1	LPQNIPPLT	23.3
992.6	114.1	LPQNIPPLT	47.6
496.8	114.1	LPQNIPPLT	23.3
992.6	103.0	LPQNIPPLT	47.6
496.8	103.0	LPQNIPPLT	23.3

992.6	86.1	LPQNIPPLT	47.6
496.8	86.1	LPQNIPPLT	23.3
425.3	338.2	LPVP	19.8
425.3	327.2	LPVP	19.8
425.3	312.2	LPVP	19.8
425.3	310.2	LPVP	19.8
425.3	295.2	LPVP	19.8
425.3	282.2	LPVP	19.8
425.3	241.1	LPVP	19.8
425.3	228.2	LPVP	19.8
425.3	215.1	LPVP	19.8
425.3	211.1	LPVP	19.8
425.3	198.1	LPVP	19.8
425.3	183.1	LPVP	19.8
425.3	142.1	LPVP	19.8
425.3	131.1	LPVP	19.8
425.3	116.1	LPVP	19.8
425.3	114.1	LPVP	19.8
425.3	99.0	LPVP	19.8
425.3	86.1	LPVP	19.8
553.3	466.2	LPVPQ	26.1
553.3	440.3	LPVPQ	26.1
553.3	424.3	LPVPQ	26.1
553.3	423.2	LPVPQ	26.1
553.3	407.3	LPVPQ	26.1
553.3	379.3	LPVPQ	26.1
553.3	369.2	LPVPQ	26.1
553.3	343.2	LPVPQ	26.1
553.3	327.2	LPVPQ	26.1
553.3	326.2	LPVPQ	26.1

553.3	310.2	LPVPQ	26.1
553.3	282.2	LPVPQ	26.1
553.3	270.1	LPVPQ	26.1
553.3	244.1	LPVPQ	26.1
553.3	228.2	LPVPQ	26.1
553.3	227.1	LPVPQ	26.1
553.3	211.1	LPVPQ	26.1
553.3	183.1	LPVPQ	26.1
553.3	173.1	LPVPQ	26.1
553.3	147.1	LPVPQ	26.1
553.3	131.1	LPVPQ	26.1
553.3	130.1	LPVPQ	26.1
553.3	114.1	LPVPQ	26.1
553.3	86.1	LPVPQ	26.1
652.3	565.2	LPYPY	31.0
652.3	539.3	LPYPY	31.0
652.3	522.2	LPYPY	31.0
652.3	488.3	LPYPY	31.0
652.3	471.3	LPYPY	31.0
652.3	468.2	LPYPY	31.0
652.3	443.3	LPYPY	31.0
652.3	442.2	LPYPY	31.0
652.3	425.2	LPYPY	31.0
652.3	391.2	LPYPY	31.0
652.3	374.2	LPYPY	31.0
652.3	346.2	LPYPY	31.0
652.3	305.1	LPYPY	31.0
652.3	279.1	LPYPY	31.0
652.3	262.1	LPYPY	31.0
652.3	228.2	LPYPY	31.0

652.3	211.1	LPYPY	31.0
652.3	208.1	LPYPY	31.0
652.3	183.1	LPYPY	31.0
652.3	182.1	LPYPY	31.0
652.3	165.1	LPYPY	31.0
652.3	131.1	LPYPY	31.0
652.3	114.1	LPYPY	31.0
652.3	86.1	LPYPY	31.0
973.5	886.4	LQAFEPLR	46.7
487.3	886.4	LQAFEPLR	22.9
973.5	860.5	LQAFEPLR	46.7
487.3	860.5	LQAFEPLR	22.9
973.5	843.4	LQAFEPLR	46.7
487.3	843.4	LQAFEPLR	22.9
973.5	816.5	LQAFEPLR	46.7
487.3	816.5	LQAFEPLR	22.9
973.5	799.4	LQAFEPLR	46.7
487.3	799.4	LQAFEPLR	22.9
973.5	771.4	LQAFEPLR	46.7
487.3	771.4	LQAFEPLR	22.9
973.5	758.4	LQAFEPLR	46.7
487.3	758.4	LQAFEPLR	22.9
973.5	732.4	LQAFEPLR	46.7
487.3	732.4	LQAFEPLR	22.9
973.5	715.4	LQAFEPLR	46.7
487.3	715.4	LQAFEPLR	22.9
973.5	703.4	LQAFEPLR	46.7
487.3	703.4	LQAFEPLR	22.9
973.5	687.3	LQAFEPLR	46.7
487.3	687.3	LQAFEPLR	22.9

973.5	686.4	LQAFEPLR	46.7
487.3	686.4	LQAFEPLR	22.9
973.5	661.4	LQAFEPLR	46.7
487.3	661.4	LQAFEPLR	22.9
973.5	658.4	LQAFEPLR	46.7
487.3	658.4	LQAFEPLR	22.9
973.5	644.3	LQAFEPLR	46.7
487.3	644.3	LQAFEPLR	22.9
973.5	606.3	LQAFEPLR	46.7
487.3	606.3	LQAFEPLR	22.9
973.5	589.3	LQAFEPLR	46.7
487.3	589.3	LQAFEPLR	22.9
973.5	561.3	LQAFEPLR	46.7
487.3	561.3	LQAFEPLR	22.9
973.5	540.3	LQAFEPLR	46.7
487.3	540.3	LQAFEPLR	22.9
973.5	514.3	LQAFEPLR	46.7
487.3	514.3	LQAFEPLR	22.9
973.5	497.3	LQAFEPLR	46.7
487.3	497.3	LQAFEPLR	22.9
973.5	477.3	LQAFEPLR	46.7
487.3	477.3	LQAFEPLR	22.9
973.5	460.3	LQAFEPLR	46.7
487.3	460.3	LQAFEPLR	22.9
973.5	432.3	LQAFEPLR	46.7
487.3	432.3	LQAFEPLR	22.9
973.5	411.2	LQAFEPLR	46.7
487.3	411.2	LQAFEPLR	22.9
973.5	385.3	LQAFEPLR	46.7
487.3	385.3	LQAFEPLR	22.9

973.5	368.2	LQAFEPLR	46.7
487.3	368.2	LQAFEPLR	22.9
973.5	330.2	LQAFEPLR	46.7
487.3	330.2	LQAFEPLR	22.9
973.5	314.2	LQAFEPLR	46.7
487.3	314.2	LQAFEPLR	22.9
973.5	313.2	LQAFEPLR	46.7
487.3	313.2	LQAFEPLR	22.9
973.5	288.2	LQAFEPLR	46.7
487.3	288.2	LQAFEPLR	22.9
973.5	285.2	LQAFEPLR	46.7
487.3	285.2	LQAFEPLR	22.9
973.5	271.2	LQAFEPLR	46.7
487.3	271.2	LQAFEPLR	22.9
973.5	259.2	LQAFEPLR	46.7
487.3	259.2	LQAFEPLR	22.9
973.5	242.2	LQAFEPLR	46.7
487.3	242.2	LQAFEPLR	22.9
973.5	214.2	LQAFEPLR	46.7
487.3	214.2	LQAFEPLR	22.9
973.5	201.1	LQAFEPLR	46.7
487.3	201.1	LQAFEPLR	22.9
973.5	175.1	LQAFEPLR	46.7
487.3	175.1	LQAFEPLR	22.9
973.5	158.1	LQAFEPLR	46.7
487.3	158.1	LQAFEPLR	22.9
973.5	131.1	LQAFEPLR	46.7
487.3	131.1	LQAFEPLR	22.9
973.5	114.1	LQAFEPLR	46.7
487.3	114.1	LQAFEPLR	22.9

973.5	86.1	LQAFEPLR	46.7
487.3	86.1	LQAFEPLR	22.9
357.2	270.1	LQP	16.5
179.1	270.1	LQP	10.0
357.2	259.2	LQP	16.5
179.1	259.2	LQP	10.0
357.2	244.1	LQP	16.5
179.1	244.1	LQP	10.0
357.2	242.2	LQP	16.5
179.1	242.2	LQP	10.0
357.2	227.1	LQP	16.5
179.1	227.1	LQP	10.0
357.2	214.2	LQP	16.5
179.1	214.2	LQP	10.0
357.2	142.1	LQP	16.5
179.1	142.1	LQP	10.0
357.2	131.1	LQP	16.5
179.1	131.1	LQP	10.0
357.2	116.1	LQP	16.5
179.1	116.1	LQP	10.0
357.2	114.1	LQP	16.5
179.1	114.1	LQP	10.0
357.2	99.0	LQP	16.5
179.1	99.0	LQP	10.0
357.2	86.1	LQP	16.5
179.1	86.1	LQP	10.0
288.2	201.1	LR	13.1
144.6	201.1	LR	10.0
288.2	175.1	LR	13.1
144.6	175.1	LR	10.0

288.2	158.1	LR	13.1
144.6	158.1	LR	10.0
288.2	131.1	LR	13.1
144.6	131.1	LR	10.0
288.2	114.1	LR	13.1
144.6	114.1	LR	10.0
288.2	86.1	LR	13.1
144.6	86.1	LR	10.0
936.4	849.3	LTFPGSAED	44.9
468.7	849.3	LTFPGSAED	22.0
936.4	823.3	LTFPGSAED	44.9
468.7	823.3	LTFPGSAED	22.0
936.4	820.4	LTFPGSAED	44.9
468.7	820.4	LTFPGSAED	22.0
936.4	806.3	LTFPGSAED	44.9
468.7	806.3	LTFPGSAED	22.0
936.4	803.4	LTFPGSAED	44.9
468.7	803.4	LTFPGSAED	22.0
936.4	775.4	LTFPGSAED	44.9
468.7	775.4	LTFPGSAED	22.0
936.4	748.3	LTFPGSAED	44.9
468.7	748.3	LTFPGSAED	22.0
936.4	722.3	LTFPGSAED	44.9
468.7	722.3	LTFPGSAED	22.0
936.4	705.3	LTFPGSAED	44.9
468.7	705.3	LTFPGSAED	22.0
936.4	691.4	LTFPGSAED	44.9
468.7	691.4	LTFPGSAED	22.0
936.4	674.4	LTFPGSAED	44.9
468.7	674.4	LTFPGSAED	22.0

936.4	646.4	LTFPGSAED	44.9
468.7	646.4	LTFPGSAED	22.0
936.4	620.3	LTFPGSAED	44.9
468.7	620.3	LTFPGSAED	22.0
936.4	603.3	LTFPGSAED	44.9
468.7	603.3	LTFPGSAED	22.0
936.4	601.2	LTFPGSAED	44.9
468.7	601.2	LTFPGSAED	22.0
936.4	575.3	LTFPGSAED	44.9
468.7	575.3	LTFPGSAED	22.0
936.4	575.2	LTFPGSAED	44.9
468.7	575.2	LTFPGSAED	22.0
936.4	558.2	LTFPGSAED	44.9
468.7	558.2	LTFPGSAED	22.0
936.4	533.3	LTFPGSAED	44.9
468.7	533.3	LTFPGSAED	22.0
936.4	516.3	LTFPGSAED	44.9
468.7	516.3	LTFPGSAED	22.0
936.4	504.2	LTFPGSAED	44.9
468.7	504.2	LTFPGSAED	22.0
936.4	488.3	LTFPGSAED	44.9
468.7	488.3	LTFPGSAED	22.0
936.4	478.2	LTFPGSAED	44.9
468.7	478.2	LTFPGSAED	22.0
936.4	476.3	LTFPGSAED	44.9
468.7	476.3	LTFPGSAED	22.0
936.4	461.2	LTFPGSAED	44.9
468.7	461.2	LTFPGSAED	22.0
936.4	459.3	LTFPGSAED	44.9
468.7	459.3	LTFPGSAED	22.0

936.4	447.1	LTFPGSAED	44.9
468.7	447.1	LTFPGSAED	22.0
936.4	431.3	LTFPGSAED	44.9
468.7	431.3	LTFPGSAED	22.0
936.4	421.2	LTFPGSAED	44.9
468.7	421.2	LTFPGSAED	22.0
936.4	404.1	LTFPGSAED	44.9
468.7	404.1	LTFPGSAED	22.0
936.4	379.2	LTFPGSAED	44.9
468.7	379.2	LTFPGSAED	22.0
936.4	362.2	LTFPGSAED	44.9
468.7	362.2	LTFPGSAED	22.0
936.4	360.1	LTFPGSAED	44.9
468.7	360.1	LTFPGSAED	22.0
936.4	334.2	LTFPGSAED	44.9
468.7	334.2	LTFPGSAED	22.0
936.4	334.1	LTFPGSAED	44.9
468.7	334.1	LTFPGSAED	22.0
936.4	317.1	LTFPGSAED	44.9
468.7	317.1	LTFPGSAED	22.0
936.4	289.1	LTFPGSAED	44.9
468.7	289.1	LTFPGSAED	22.0
936.4	263.1	LTFPGSAED	44.9
468.7	263.1	LTFPGSAED	22.0
936.4	246.1	LTFPGSAED	44.9
468.7	246.1	LTFPGSAED	22.0
936.4	232.2	LTFPGSAED	44.9
468.7	232.2	LTFPGSAED	22.0
936.4	215.1	LTFPGSAED	44.9
468.7	215.1	LTFPGSAED	22.0

936.4	187.1	LTFPGSAED	44.9
468.7	187.1	LTFPGSAED	22.0
936.4	160.0	LTFPGSAED	44.9
468.7	160.0	LTFPGSAED	22.0
936.4	134.0	LTFPGSAED	44.9
468.7	134.0	LTFPGSAED	22.0
936.4	131.1	LTFPGSAED	44.9
468.7	131.1	LTFPGSAED	22.0
936.4	117.0	LTFPGSAED	44.9
468.7	117.0	LTFPGSAED	22.0
936.4	114.1	LTFPGSAED	44.9
468.7	114.1	LTFPGSAED	22.0
936.4	86.1	LTFPGSAED	44.9
468.7	86.1	LTFPGSAED	22.0
318.2	231.1	LW	14.6
159.6	231.1	LW	10.0
318.2	205.1	LW	14.6
159.6	205.1	LW	10.0
318.2	188.1	LW	14.6
159.6	188.1	LW	10.0
318.2	131.1	LW	14.6
159.6	131.1	LW	10.0
318.2	114.1	LW	14.6
159.6	114.1	LW	10.0
318.2	86.1	LW	14.6
159.6	86.1	LW	10.0
441.2	336.1	MGSF	20.6
221.1	336.1	MGSF	10.0
441.2	310.1	MGSF	20.6
221.1	310.1	MGSF	10.0

441.2	293.1	MGSF	20.6
221.1	293.1	MGSF	10.0
441.2	293.1	MGSF	20.6
221.1	293.1	MGSF	10.0
441.2	279.1	MGSF	20.6
221.1	279.1	MGSF	10.0
441.2	276.1	MGSF	20.6
221.1	276.1	MGSF	10.0
441.2	253.1	MGSF	20.6
221.1	253.1	MGSF	10.0
441.2	248.1	MGSF	20.6
221.1	248.1	MGSF	10.0
441.2	236.1	MGSF	20.6
221.1	236.1	MGSF	10.0
441.2	206.1	MGSF	20.6
221.1	206.1	MGSF	10.0
441.2	192.1	MGSF	20.6
221.1	192.1	MGSF	10.0
441.2	189.1	MGSF	20.6
221.1	189.1	MGSF	10.0
441.2	166.1	MGSF	20.6
221.1	166.1	MGSF	10.0
441.2	161.1	MGSF	20.6
221.1	161.1	MGSF	10.0
441.2	149.1	MGSF	20.6
221.1	149.1	MGSF	10.0
441.2	149.1	MGSF	20.6
221.1	149.1	MGSF	10.0
441.2	132.0	MGSF	20.6
221.1	132.0	MGSF	10.0

441.2	104.1	MGSF	20.6
221.1	104.1	MGSF	10.0
247.1	149.1	MP	11.1
124.1	149.1	MP	10.0
247.1	142.1	MP	11.1
124.1	142.1	MP	10.0
247.1	132.0	MP	11.1
124.1	132.0	MP	10.0
247.1	116.1	MP	11.1
124.1	116.1	MP	10.0
247.1	104.1	MP	11.1
124.1	104.1	MP	10.0
247.1	99.0	MP	11.1
124.1	99.0	MP	10.0
545.3	473.3	MPVQA	25.7
273.1	473.3	MPVQA	12.4
545.3	456.2	MPVQA	25.7
273.1	456.2	MPVQA	12.4
545.3	440.2	MPVQA	25.7
273.1	440.2	MPVQA	12.4
545.3	428.2	MPVQA	25.7
273.1	428.2	MPVQA	12.4
545.3	414.2	MPVQA	25.7
273.1	414.2	MPVQA	12.4
545.3	397.2	MPVQA	25.7
273.1	397.2	MPVQA	12.4
545.3	345.2	MPVQA	25.7
273.1	345.2	MPVQA	12.4
545.3	343.2	MPVQA	25.7
273.1	343.2	MPVQA	12.4

545.3	328.2	MPVQA	25.7
273.1	328.2	MPVQA	12.4
545.3	317.2	MPVQA	25.7
273.1	317.2	MPVQA	12.4
545.3	300.2	MPVQA	25.7
273.1	300.2	MPVQA	12.4
545.3	300.2	MPVQA	25.7
273.1	300.2	MPVQA	12.4
545.3	246.1	MPVQA	25.7
273.1	246.1	MPVQA	12.4
545.3	244.1	MPVQA	25.7
273.1	244.1	MPVQA	12.4
545.3	229.1	MPVQA	25.7
273.1	229.1	MPVQA	12.4
545.3	218.1	MPVQA	25.7
273.1	218.1	MPVQA	12.4
545.3	201.1	MPVQA	25.7
273.1	201.1	MPVQA	12.4
545.3	201.1	MPVQA	25.7
273.1	201.1	MPVQA	12.4
545.3	149.1	MPVQA	25.7
273.1	149.1	MPVQA	12.4
545.3	132.0	MPVQA	25.7
273.1	132.0	MPVQA	12.4
545.3	116.0	MPVQA	25.7
273.1	116.0	MPVQA	12.4
545.3	104.1	MPVQA	25.7
273.1	104.1	MPVQA	12.4
545.3	90.1	MPVQA	25.7
273.1	90.1	MPVQA	12.4

545.3	73.0	MPVQA	25.7
273.1	73.0	MPVQA	12.4
336.1	231.1	MW	15.5
168.6	231.1	MW	10.0
336.1	205.1	MW	15.5
168.6	205.1	MW	10.0
336.1	188.1	MW	15.5
168.6	188.1	MW	10.0
336.1	149.1	MW	15.5
168.6	149.1	MW	10.0
336.1	132.0	MW	15.5
168.6	132.0	MW	10.0
336.1	104.1	MW	15.5
168.6	104.1	MW	10.0
798.5	710.5	NLGIILR	38.1
399.8	710.5	NLGIILR	18.6
798.5	684.5	NLGIILR	38.1
399.8	684.5	NLGIILR	18.6
798.5	667.5	NLGIILR	38.1
399.8	667.5	NLGIILR	18.6
798.5	641.4	NLGIILR	38.1
399.8	641.4	NLGIILR	18.6
798.5	624.4	NLGIILR	38.1
399.8	624.4	NLGIILR	18.6
798.5	597.4	NLGIILR	38.1
399.8	597.4	NLGIILR	18.6
798.5	596.4	NLGIILR	38.1
399.8	596.4	NLGIILR	18.6
798.5	571.4	NLGIILR	38.1
399.8	571.4	NLGIILR	18.6

798.5	554.4	NLGIILR	38.1
399.8	554.4	NLGIILR	18.6
798.5	540.4	NLGIILR	38.1
399.8	540.4	NLGIILR	18.6
798.5	528.4	NLGIILR	38.1
399.8	528.4	NLGIILR	18.6
798.5	514.4	NLGIILR	38.1
399.8	514.4	NLGIILR	18.6
798.5	511.3	NLGIILR	38.1
399.8	511.3	NLGIILR	18.6
798.5	497.3	NLGIILR	38.1
399.8	497.3	NLGIILR	18.6
798.5	483.3	NLGIILR	38.1
399.8	483.3	NLGIILR	18.6
798.5	427.3	NLGIILR	38.1
399.8	427.3	NLGIILR	18.6
798.5	415.3	NLGIILR	38.1
399.8	415.3	NLGIILR	18.6
798.5	401.3	NLGIILR	38.1
399.8	401.3	NLGIILR	18.6
798.5	398.2	NLGIILR	38.1
399.8	398.2	NLGIILR	18.6
798.5	384.3	NLGIILR	38.1
399.8	384.3	NLGIILR	18.6
798.5	370.2	NLGIILR	38.1
399.8	370.2	NLGIILR	18.6
798.5	314.2	NLGIILR	38.1
399.8	314.2	NLGIILR	18.6
798.5	302.2	NLGIILR	38.1
399.8	302.2	NLGIILR	18.6

798.5	288.2	NLGIILR	38.1
399.8	288.2	NLGIILR	18.6
798.5	285.2	NLGIILR	38.1
399.8	285.2	NLGIILR	18.6
798.5	271.2	NLGIILR	38.1
399.8	271.2	NLGIILR	18.6
798.5	257.2	NLGIILR	38.1
399.8	257.2	NLGIILR	18.6
798.5	245.2	NLGIILR	38.1
399.8	245.2	NLGIILR	18.6
798.5	228.1	NLGIILR	38.1
399.8	228.1	NLGIILR	18.6
798.5	201.1	NLGIILR	38.1
399.8	201.1	NLGIILR	18.6
798.5	200.1	NLGIILR	38.1
399.8	200.1	NLGIILR	18.6
798.5	175.1	NLGIILR	38.1
399.8	175.1	NLGIILR	18.6
798.5	158.1	NLGIILR	38.1
399.8	158.1	NLGIILR	18.6
798.5	132.1	NLGIILR	38.1
399.8	132.1	NLGIILR	18.6
798.5	115.1	NLGIILR	38.1
399.8	115.1	NLGIILR	18.6
798.5	87.1	NLGIILR	38.1
399.8	87.1	NLGIILR	18.6
681.3	1247.6	PAC[CAM]GGFWISGRPG	32.4
681.3	1219.5	PAC[CAM]GGFWISGRPG	32.4
681.3	1206.6	PAC[CAM]GGFWISGRPG	32.4
681.3	1193.6	PAC[CAM]GGFWISGRPG	32.4

681.3	1189.6	PAC[CAM]GGFWISGRPG	32.4
681.3	1176.5	PAC[CAM]GGFWISGRPG	32.4
681.3	1161562.0	PAC[CAM]GGFWISGRPG	32.4
681.3	1059.5	PAC[CAM]GGFWISGRPG	32.4
681.3	1050.5	PAC[CAM]GGFWISGRPG	32.4
681.3	1033.5	PAC[CAM]GGFWISGRPG	32.4
681.3	1033.5	PAC[CAM]GGFWISGRPG	32.4
681.3	1016.5	PAC[CAM]GGFWISGRPG	32.4
681.3	1.005.461	PAC[CAM]GGFWISGRPG	32.4
681.3	1.002.479	PAC[CAM]GGFWISGRPG	32.4
681.3	993.5	PAC[CAM]GGFWISGRPG	32.4
681.3	976.5	PAC[CAM]GGFWISGRPG	32.4
681.3	976.4	PAC[CAM]GGFWISGRPG	32.4
681.3	959.5	PAC[CAM]GGFWISGRPG	32.4
681.3	948.4	PAC[CAM]GGFWISGRPG	32.4
681.3	945.5	PAC[CAM]GGFWISGRPG	32.4
681.3	919.5	PAC[CAM]GGFWISGRPG	32.4
681.3	906.4	PAC[CAM]GGFWISGRPG	32.4
681.3	902.5	PAC[CAM]GGFWISGRPG	32.4
681.3	889.4	PAC[CAM]GGFWISGRPG	32.4
681.3	861.4	PAC[CAM]GGFWISGRPG	32.4
681.3	798.4	PAC[CAM]GGFWISGRPG	32.4
681.3	793.3	PAC[CAM]GGFWISGRPG	32.4
681.3	776.3	PAC[CAM]GGFWISGRPG	32.4
681.3	772.4	PAC[CAM]GGFWISGRPG	32.4
681.3	755.4	PAC[CAM]GGFWISGRPG	32.4
681.3	748.3	PAC[CAM]GGFWISGRPG	32.4
681.3	612.3	PAC[CAM]GGFWISGRPG	32.4
681.3	607.3	PAC[CAM]GGFWISGRPG	32.4
681.3	590.2	PAC[CAM]GGFWISGRPG	32.4

681.3	586.3	PAC[CAM]GGFWISGRPG	32.4
681.3	569.3	PAC[CAM]GGFWISGRPG	32.4
681.3	562.2	PAC[CAM]GGFWISGRPG	32.4
681.3	499.2	PAC[CAM]GGFWISGRPG	32.4
681.3	473.2	PAC[CAM]GGFWISGRPG	32.4
681.3	460.2	PAC[CAM]GGFWISGRPG	32.4
681.3	456.2	PAC[CAM]GGFWISGRPG	32.4
681.3	443.2	PAC[CAM]GGFWISGRPG	32.4
681.3	415.2	PAC[CAM]GGFWISGRPG	32.4
681.3	412.2	PAC[CAM]GGFWISGRPG	32.4
681.3	403.2	PAC[CAM]GGFWISGRPG	32.4
681.3	386.2	PAC[CAM]GGFWISGRPG	32.4
681.3	386.1	PAC[CAM]GGFWISGRPG	32.4
681.3	369.2	PAC[CAM]GGFWISGRPG	32.4
681.3	358.2	PAC[CAM]GGFWISGRPG	32.4
681.3	355.2	PAC[CAM]GGFWISGRPG	32.4
681.3	346.2	PAC[CAM]GGFWISGRPG	32.4
681.3	329.2	PAC[CAM]GGFWISGRPG	32.4
681.3	329.1	PAC[CAM]GGFWISGRPG	32.4
681.3	312.2	PAC[CAM]GGFWISGRPG	32.4
681.3	301.1	PAC[CAM]GGFWISGRPG	32.4
681.3	199.1	PAC[CAM]GGFWISGRPG	32.4
681.3	186.1	PAC[CAM]GGFWISGRPG	32.4
681.3	173.1	PAC[CAM]GGFWISGRPG	32.4
681.3	169.1	PAC[CAM]GGFWISGRPG	32.4
681.3	156.1	PAC[CAM]GGFWISGRPG	32.4
681.3	141.1	PAC[CAM]GGFWISGRPG	32.4
681.3	115.1	PAC[CAM]GGFWISGRPG	32.4
681.3	102.0	PAC[CAM]GGFWISGRPG	32.4
681.3	98.1	PAC[CAM]GGFWISGRPG	32.4

681.3	76.0	PAC[CAM]GGFWISGRPG	32.4
681.3	70.1	PAC[CAM]GGFWISGRPG	32.4
681.3	59.0	PAC[CAM]GGFWISGRPG	32.4
798.4	727.3	PAGDDAPR	38.1
399.7	727.3	PAGDDAPR	18.6
798.4	701.3	PAGDDAPR	38.1
399.7	701.3	PAGDDAPR	18.6
798.4	684.3	PAGDDAPR	38.1
399.7	684.3	PAGDDAPR	18.6
798.4	656.3	PAGDDAPR	38.1
399.7	656.3	PAGDDAPR	18.6
798.4	641.3	PAGDDAPR	38.1
399.7	641.3	PAGDDAPR	18.6
798.4	630.3	PAGDDAPR	38.1
399.7	630.3	PAGDDAPR	18.6
798.4	624.3	PAGDDAPR	38.1
399.7	624.3	PAGDDAPR	18.6
798.4	613.3	PAGDDAPR	38.1
399.7	613.3	PAGDDAPR	18.6
798.4	599.2	PAGDDAPR	38.1
399.7	599.2	PAGDDAPR	18.6
798.4	596.3	PAGDDAPR	38.1
399.7	596.3	PAGDDAPR	18.6
798.4	573.3	PAGDDAPR	38.1
399.7	573.3	PAGDDAPR	18.6
798.4	556.2	PAGDDAPR	38.1
399.7	556.2	PAGDDAPR	18.6
798.4	544.2	PAGDDAPR	38.1
399.7	544.2	PAGDDAPR	18.6
798.4	527.2	PAGDDAPR	38.1

399.7	527.2	PAGDDAPR	18.6
798.4	499.2	PAGDDAPR	38.1
399.7	499.2	PAGDDAPR	18.6
798.4	484.2	PAGDDAPR	38.1
399.7	484.2	PAGDDAPR	18.6
798.4	473.2	PAGDDAPR	38.1
399.7	473.2	PAGDDAPR	18.6
798.4	458.2	PAGDDAPR	38.1
399.7	458.2	PAGDDAPR	18.6
798.4	456.2	PAGDDAPR	38.1
399.7	456.2	PAGDDAPR	18.6
798.4	441.2	PAGDDAPR	38.1
399.7	441.2	PAGDDAPR	18.6
798.4	428.2	PAGDDAPR	38.1
399.7	428.2	PAGDDAPR	18.6
798.4	369.2	PAGDDAPR	38.1
399.7	369.2	PAGDDAPR	18.6
798.4	358.2	PAGDDAPR	38.1
399.7	358.2	PAGDDAPR	18.6
798.4	343.2	PAGDDAPR	38.1
399.7	343.2	PAGDDAPR	18.6
798.4	341.1	PAGDDAPR	38.1
399.7	341.1	PAGDDAPR	18.6
798.4	326.2	PAGDDAPR	38.1
399.7	326.2	PAGDDAPR	18.6
798.4	313.2	PAGDDAPR	38.1
399.7	313.2	PAGDDAPR	18.6
798.4	298.2	PAGDDAPR	38.1
399.7	298.2	PAGDDAPR	18.6
798.4	272.2	PAGDDAPR	38.1

399.7	272.2	PAGDDAPR	18.6
798.4	255.1	PAGDDAPR	38.1
399.7	255.1	PAGDDAPR	18.6
798.4	243.1	PAGDDAPR	38.1
399.7	243.1	PAGDDAPR	18.6
798.4	226.1	PAGDDAPR	38.1
399.7	226.1	PAGDDAPR	18.6
798.4	201.1	PAGDDAPR	38.1
399.7	201.1	PAGDDAPR	18.6
798.4	198.1	PAGDDAPR	38.1
399.7	198.1	PAGDDAPR	18.6
798.4	186.1	PAGDDAPR	38.1
399.7	186.1	PAGDDAPR	18.6
798.4	175.1	PAGDDAPR	38.1
399.7	175.1	PAGDDAPR	18.6
798.4	169.1	PAGDDAPR	38.1
399.7	169.1	PAGDDAPR	18.6
798.4	158.1	PAGDDAPR	38.1
399.7	158.1	PAGDDAPR	18.6
798.4	141.1	PAGDDAPR	38.1
399.7	141.1	PAGDDAPR	18.6
798.4	115.1	PAGDDAPR	38.1
399.7	115.1	PAGDDAPR	18.6
798.4	98.1	PAGDDAPR	38.1
399.7	98.1	PAGDDAPR	18.6
798.4	70.1	PAGDDAPR	38.1
399.7	70.1	PAGDDAPR	18.6
869.4	798.3	PAGDDAPRA	41.6
435.2	798.3	PAGDDAPRA	20.3
869.4	797.4	PAGDDAPRA	41.6

435.2	797.4	PAGDDAPRA	20.3
869.4	780.4	PAGDDAPRA	41.6
435.2	780.4	PAGDDAPRA	20.3
869.4	772.4	PAGDDAPRA	41.6
435.2	772.4	PAGDDAPRA	20.3
869.4	755.3	PAGDDAPRA	41.6
435.2	755.3	PAGDDAPRA	20.3
869.4	752.4	PAGDDAPRA	41.6
435.2	752.4	PAGDDAPRA	20.3
869.4	727.3	PAGDDAPRA	41.6
435.2	727.3	PAGDDAPRA	20.3
869.4	701.3	PAGDDAPRA	41.6
435.2	701.3	PAGDDAPRA	20.3
869.4	684.3	PAGDDAPRA	41.6
435.2	684.3	PAGDDAPRA	20.3
869.4	670.3	PAGDDAPRA	41.6
435.2	670.3	PAGDDAPRA	20.3
869.4	644.3	PAGDDAPRA	41.6
435.2	644.3	PAGDDAPRA	20.3
869.4	641.3	PAGDDAPRA	41.6
435.2	641.3	PAGDDAPRA	20.3
869.4	627.3	PAGDDAPRA	41.6
435.2	627.3	PAGDDAPRA	20.3
869.4	624.3	PAGDDAPRA	41.6
435.2	624.3	PAGDDAPRA	20.3
869.4	596.3	PAGDDAPRA	41.6
435.2	596.3	PAGDDAPRA	20.3
869.4	555.3	PAGDDAPRA	41.6
435.2	555.3	PAGDDAPRA	20.3
869.4	544.2	PAGDDAPRA	41.6

435.2	544.2	PAGDDAPRA	20.3
869.4	529.3	PAGDDAPRA	41.6
435.2	529.3	PAGDDAPRA	20.3
869.4	527.2	PAGDDAPRA	41.6
435.2	527.2	PAGDDAPRA	20.3
869.4	512.2	PAGDDAPRA	41.6
435.2	512.2	PAGDDAPRA	20.3
869.4	499.2	PAGDDAPRA	41.6
435.2	499.2	PAGDDAPRA	20.3
869.4	473.2	PAGDDAPRA	41.6
435.2	473.2	PAGDDAPRA	20.3
869.4	456.2	PAGDDAPRA	41.6
435.2	456.2	PAGDDAPRA	20.3
869.4	440.2	PAGDDAPRA	41.6
435.2	440.2	PAGDDAPRA	20.3
869.4	428.2	PAGDDAPRA	41.6
435.2	428.2	PAGDDAPRA	20.3
869.4	414.2	PAGDDAPRA	41.6
435.2	414.2	PAGDDAPRA	20.3
869.4	397.2	PAGDDAPRA	41.6
435.2	397.2	PAGDDAPRA	20.3
869.4	369.2	PAGDDAPRA	41.6
435.2	369.2	PAGDDAPRA	20.3
869.4	358.2	PAGDDAPRA	41.6
435.2	358.2	PAGDDAPRA	20.3
869.4	343.2	PAGDDAPRA	41.6
435.2	343.2	PAGDDAPRA	20.3
869.4	341.1	PAGDDAPRA	41.6
435.2	341.1	PAGDDAPRA	20.3
869.4	326.2	PAGDDAPRA	41.6

435.2	326.2	PAGDDAPRA	20.3
869.4	313.2	PAGDDAPRA	41.6
435.2	313.2	PAGDDAPRA	20.3
869.4	272.1	PAGDDAPRA	41.6
435.2	272.1	PAGDDAPRA	20.3
869.4	246.2	PAGDDAPRA	41.6
435.2	246.2	PAGDDAPRA	20.3
869.4	243.1	PAGDDAPRA	41.6
435.2	243.1	PAGDDAPRA	20.3
869.4	229.1	PAGDDAPRA	41.6
435.2	229.1	PAGDDAPRA	20.3
869.4	226.1	PAGDDAPRA	41.6
435.2	226.1	PAGDDAPRA	20.3
869.4	198.1	PAGDDAPRA	41.6
435.2	198.1	PAGDDAPRA	20.3
869.4	186.1	PAGDDAPRA	41.6
435.2	186.1	PAGDDAPRA	20.3
869.4	169.1	PAGDDAPRA	41.6
435.2	169.1	PAGDDAPRA	20.3
869.4	141.1	PAGDDAPRA	41.6
435.2	141.1	PAGDDAPRA	20.3
869.4	116.0	PAGDDAPRA	41.6
435.2	116.0	PAGDDAPRA	20.3
869.4	115.1	PAGDDAPRA	41.6
435.2	115.1	PAGDDAPRA	20.3
869.4	98.1	PAGDDAPRA	41.6
435.2	98.1	PAGDDAPRA	20.3
869.4	90.1	PAGDDAPRA	41.6
435.2	90.1	PAGDDAPRA	20.3
869.4	73.0	PAGDDAPRA	41.6

435.2	73.0	PAGDDAPRA	20.3
869.4	70.1	PAGDDAPRA	41.6
435.2	70.1	PAGDDAPRA	20.3
300.2	229.1	PAL	13.7
150.6	229.1	PAL	10.0
300.2	203.1	PAL	13.7
150.6	203.1	PAL	10.0
300.2	186.1	PAL	13.7
150.6	186.1	PAL	10.0
300.2	186.1	PAL	13.7
150.6	186.1	PAL	10.0
300.2	169.1	PAL	13.7
150.6	169.1	PAL	10.0
300.2	158.1	PAL	13.7
150.6	158.1	PAL	10.0
300.2	141.1	PAL	13.7
150.6	141.1	PAL	10.0
300.2	132.1	PAL	13.7
150.6	132.1	PAL	10.0
300.2	115.1	PAL	13.7
150.6	115.1	PAL	10.0
300.2	115.1	PAL	13.7
150.6	115.1	PAL	10.0
300.2	98.1	PAL	13.7
150.6	98.1	PAL	10.0
300.2	70.1	PAL	13.7
150.6	70.1	PAL	10.0
1014.5	912.5	QPHQPLPPT	48.7
507.8	912.5	QPHQPLPPT	23.9
1014.5	912.5	QPHQPLPPT	48.7

507.8	912.5	QPHQPLPPT	23.9
1014.5	895.5	QPHQPLPPT	48.7
507.8	895.5	QPHQPLPPT	23.9
1014.5	886.5	QPHQPLPPT	48.7
507.8	886.5	QPHQPLPPT	23.9
1014.5	869.5	QPHQPLPPT	48.7
507.8	869.5	QPHQPLPPT	23.9
1014.5	867.5	QPHQPLPPT	48.7
507.8	867.5	QPHQPLPPT	23.9
1014.5	815.5	QPHQPLPPT	48.7
507.8	815.5	QPHQPLPPT	23.9
1014.5	815.4	QPHQPLPPT	48.7
507.8	815.4	QPHQPLPPT	23.9
1014.5	798.4	QPHQPLPPT	48.7
507.8	798.4	QPHQPLPPT	23.9
1014.5	789.4	QPHQPLPPT	48.7
507.8	789.4	QPHQPLPPT	23.9
1014.5	772.4	QPHQPLPPT	48.7
507.8	772.4	QPHQPLPPT	23.9
1014.5	770.4	QPHQPLPPT	48.7
507.8	770.4	QPHQPLPPT	23.9
1014.5	718.4	QPHQPLPPT	48.7
507.8	718.4	QPHQPLPPT	23.9
1014.5	701.4	QPHQPLPPT	48.7
507.8	701.4	QPHQPLPPT	23.9
1014.5	678.3	QPHQPLPPT	48.7
507.8	678.3	QPHQPLPPT	23.9
1014.5	673.4	QPHQPLPPT	48.7
507.8	673.4	QPHQPLPPT	23.9
1014.5	652.4	QPHQPLPPT	48.7

507.8	652.4	QPHQPLPPT	23.9
1014.5	635.3	QPHQPLPPT	48.7
507.8	635.3	QPHQPLPPT	23.9
1014.5	605.3	QPHQPLPPT	48.7
507.8	605.3	QPHQPLPPT	23.9
1014.5	588.3	QPHQPLPPT	48.7
507.8	588.3	QPHQPLPPT	23.9
1014.5	560.3	QPHQPLPPT	48.7
507.8	560.3	QPHQPLPPT	23.9
1014.5	550.3	QPHQPLPPT	48.7
507.8	550.3	QPHQPLPPT	23.9
1014.5	524.3	QPHQPLPPT	48.7
507.8	524.3	QPHQPLPPT	23.9
1014.5	508.3	QPHQPLPPT	48.7
507.8	508.3	QPHQPLPPT	23.9
1014.5	507.3	QPHQPLPPT	48.7
507.8	507.3	QPHQPLPPT	23.9
1014.5	491.2	QPHQPLPPT	48.7
507.8	491.2	QPHQPLPPT	23.9
1014.5	463.2	QPHQPLPPT	48.7
507.8	463.2	QPHQPLPPT	23.9
1014.5	453.2	QPHQPLPPT	48.7
507.8	453.2	QPHQPLPPT	23.9
1014.5	427.3	QPHQPLPPT	48.7
507.8	427.3	QPHQPLPPT	23.9
1014.5	410.2	QPHQPLPPT	48.7
507.8	410.2	QPHQPLPPT	23.9
1014.5	380.2	QPHQPLPPT	48.7
507.8	380.2	QPHQPLPPT	23.9
1014.5	363.2	QPHQPLPPT	48.7

507.8	363.2	QPHQPLPPT	23.9
1014.5	340.2	QPHQPLPPT	48.7
507.8	340.2	QPHQPLPPT	23.9
1014.5	335.2	QPHQPLPPT	48.7
507.8	335.2	QPHQPLPPT	23.9
1014.5	314.2	QPHQPLPPT	48.7
507.8	314.2	QPHQPLPPT	23.9
1014.5	297.1	QPHQPLPPT	48.7
507.8	297.1	QPHQPLPPT	23.9
1014.5	243.1	QPHQPLPPT	48.7
507.8	243.1	QPHQPLPPT	23.9
1014.5	243.1	QPHQPLPPT	48.7
507.8	243.1	QPHQPLPPT	23.9
1014.5	226.1	QPHQPLPPT	48.7
507.8	226.1	QPHQPLPPT	23.9
1014.5	217.1	QPHQPLPPT	48.7
507.8	217.1	QPHQPLPPT	23.9
1014.5	200.1	QPHQPLPPT	48.7
507.8	200.1	QPHQPLPPT	23.9
1014.5	198.1	QPHQPLPPT	48.7
507.8	198.1	QPHQPLPPT	23.9
1014.5	146.1	QPHQPLPPT	48.7
507.8	146.1	QPHQPLPPT	23.9
1014.5	146.0	QPHQPLPPT	48.7
507.8	146.0	QPHQPLPPT	23.9
1014.5	129.1	QPHQPLPPT	48.7
507.8	129.1	QPHQPLPPT	23.9
1014.5	120.1	QPHQPLPPT	48.7
507.8	120.1	QPHQPLPPT	23.9
1014.5	103.0	QPHQPLPPT	48.7

507.8	103.0	QPHQPLPPT	23.9
1014.5	101.1	QPHQPLPPT	48.7
507.8	101.1	QPHQPLPPT	23.9
652.4	550.3	QPLPPT	31.0
326.7	550.3	QPLPPT	15.0
652.4	550.3	QPLPPT	31.0
326.7	550.3	QPLPPT	15.0
652.4	533.3	QPLPPT	31.0
326.7	533.3	QPLPPT	15.0
652.4	524.3	QPLPPT	31.0
326.7	524.3	QPLPPT	15.0
652.4	507.3	QPLPPT	31.0
326.7	507.3	QPLPPT	15.0
652.4	505.3	QPLPPT	31.0
326.7	505.3	QPLPPT	15.0
652.4	453.3	QPLPPT	31.0
326.7	453.3	QPLPPT	15.0
652.4	453.2	QPLPPT	31.0
326.7	453.2	QPLPPT	15.0
652.4	436.3	QPLPPT	31.0
326.7	436.3	QPLPPT	15.0
652.4	427.3	QPLPPT	31.0
326.7	427.3	QPLPPT	15.0
652.4	410.2	QPLPPT	31.0
326.7	410.2	QPLPPT	15.0
652.4	408.3	QPLPPT	31.0
326.7	408.3	QPLPPT	15.0
652.4	356.2	QPLPPT	31.0
326.7	356.2	QPLPPT	15.0
652.4	340.2	QPLPPT	31.0

326.7	340.2	QPLPPT	15.0
652.4	339.2	QPLPPT	31.0
326.7	339.2	QPLPPT	15.0
652.4	314.2	QPLPPT	31.0
326.7	314.2	QPLPPT	15.0
652.4	311.2	QPLPPT	31.0
326.7	311.2	QPLPPT	15.0
652.4	297.1	QPLPPT	31.0
326.7	297.1	QPLPPT	15.0
652.4	243.1	QPLPPT	31.0
326.7	243.1	QPLPPT	15.0
652.4	243.1	QPLPPT	31.0
326.7	243.1	QPLPPT	15.0
652.4	226.1	QPLPPT	31.0
326.7	226.1	QPLPPT	15.0
652.4	217.1	QPLPPT	31.0
326.7	217.1	QPLPPT	15.0
652.4	200.1	QPLPPT	31.0
326.7	200.1	QPLPPT	15.0
652.4	198.1	QPLPPT	31.0
326.7	198.1	QPLPPT	15.0
652.4	146.1	QPLPPT	31.0
326.7	146.1	QPLPPT	15.0
652.4	146.0	QPLPPT	31.0
326.7	146.0	QPLPPT	15.0
652.4	129.1	QPLPPT	31.0
326.7	129.1	QPLPPT	15.0
652.4	120.1	QPLPPT	31.0
326.7	120.1	QPLPPT	15.0
652.4	103.0	QPLPPT	31.0

326.7	103.0	QPLPPT	15.0
652.4	101.1	QPLPPT	31.0
326.7	101.1	QPLPPT	15.0
372.2	270.1	QPQ	17.2
186.6	270.1	QPQ	10.0
372.2	244.1	QPQ	17.2
186.6	244.1	QPQ	10.0
372.2	243.1	QPQ	17.2
186.6	243.1	QPQ	10.0
372.2	227.1	QPQ	17.2
186.6	227.1	QPQ	10.0
372.2	226.1	QPQ	17.2
186.6	226.1	QPQ	10.0
372.2	198.1	QPQ	17.2
186.6	198.1	QPQ	10.0
372.2	173.1	QPQ	17.2
186.6	173.1	QPQ	10.0
372.2	147.1	QPQ	17.2
186.6	147.1	QPQ	10.0
372.2	146.1	QPQ	17.2
186.6	146.1	QPQ	10.0
372.2	130.1	QPQ	17.2
186.6	130.1	QPQ	10.0
372.2	129.1	QPQ	17.2
186.6	129.1	QPQ	10.0
372.2	101.1	QPQ	17.2
186.6	101.1	QPQ	10.0
288.2	174.1	RL	13.1
144.6	174.1	RL	10.0
288.2	158.1	RL	13.1

144.6	158.1	RL	10.0
288.2	157.1	RL	13.1
144.6	157.1	RL	10.0
288.2	132.1	RL	13.1
144.6	132.1	RL	10.0
288.2	129.1	RL	13.1
144.6	129.1	RL	10.0
288.2	115.1	RL	13.1
144.6	115.1	RL	10.0
272.2	174.1	RP	12.3
136.6	174.1	RP	10.0
272.2	157.1	RP	12.3
136.6	157.1	RP	10.0
272.2	142.1	RP	12.3
136.6	142.1	RP	10.0
272.2	129.1	RP	12.3
136.6	129.1	RP	10.0
272.2	116.1	RP	12.3
136.6	116.1	RP	10.0
272.2	99.0	RP	12.3
136.6	99.0	RP	10.0
673.4	543.3	RRRW	32.0
337.2	543.3	RRRW	15.5
673.4	517.3	RRRW	32.0
337.2	517.3	RRRW	15.5
673.4	500.3	RRRW	32.0
337.2	500.3	RRRW	15.5
673.4	486.3	RRRW	32.0
337.2	486.3	RRRW	15.5
673.4	469.3	RRRW	32.0

337.2	469.3	RRRW	15.5
673.4	441.3	RRRW	32.0
337.2	441.3	RRRW	15.5
673.4	387.2	RRRW	32.0
337.2	387.2	RRRW	15.5
673.4	361.2	RRRW	32.0
337.2	361.2	RRRW	15.5
673.4	344.2	RRRW	32.0
337.2	344.2	RRRW	15.5
673.4	330.2	RRRW	32.0
337.2	330.2	RRRW	15.5
673.4	313.2	RRRW	32.0
337.2	313.2	RRRW	15.5
673.4	285.2	RRRW	32.0
337.2	285.2	RRRW	15.5
673.4	231.1	RRRW	32.0
337.2	231.1	RRRW	15.5
673.4	205.1	RRRW	32.0
337.2	205.1	RRRW	15.5
673.4	188.1	RRRW	32.0
337.2	188.1	RRRW	15.5
673.4	174.1	RRRW	32.0
337.2	174.1	RRRW	15.5
673.4	157.1	RRRW	32.0
337.2	157.1	RRRW	15.5
673.4	129.1	RRRW	32.0
337.2	129.1	RRRW	15.5
219.1	158.1	SL	10.0
110.1	158.1	SL	10.0
219.1	132.1	SL	10.0

110.1	132.1	SL	10.0
219.1	115.1	SL	10.0
110.1	115.1	SL	10.0
219.1	105.1	SL	10.0
110.1	105.1	SL	10.0
219.1	88.0	SL	10.0
110.1	88.0	SL	10.0
219.1	60.0	SL	10.0
110.1	60.0	SL	10.0
331.2	270.1	SPQ	15.2
166.1	270.1	SPQ	10.0
331.2	244.1	SPQ	15.2
166.1	244.1	SPQ	10.0
331.2	227.1	SPQ	15.2
166.1	227.1	SPQ	10.0
331.2	202.1	SPQ	15.2
166.1	202.1	SPQ	10.0
331.2	185.1	SPQ	15.2
166.1	185.1	SPQ	10.0
331.2	173.1	SPQ	15.2
166.1	173.1	SPQ	10.0
331.2	157.1	SPQ	15.2
166.1	157.1	SPQ	10.0
331.2	147.1	SPQ	15.2
166.1	147.1	SPQ	10.0
331.2	130.1	SPQ	15.2
166.1	130.1	SPQ	10.0
331.2	105.1	SPQ	15.2
166.1	105.1	SPQ	10.0
331.2	88.0	SPQ	15.2

166.1	88.0	SPQ	10.0
331.2	60.0	SPQ	15.2
166.1	60.0	SPQ	10.0
645.4	584.3	SPVVPF	30.6
323.2	584.3	SPVVPF	14.8
645.4	558.3	SPVVPF	30.6
323.2	558.3	SPVVPF	14.8
645.4	541.3	SPVVPF	30.6
323.2	541.3	SPVVPF	14.8
645.4	497.3	SPVVPF	30.6
323.2	497.3	SPVVPF	14.8
645.4	487.3	SPVVPF	30.6
323.2	487.3	SPVVPF	14.8
645.4	480.3	SPVVPF	30.6
323.2	480.3	SPVVPF	14.8
645.4	461.3	SPVVPF	30.6
323.2	461.3	SPVVPF	14.8
645.4	452.3	SPVVPF	30.6
323.2	452.3	SPVVPF	14.8
645.4	444.2	SPVVPF	30.6
323.2	444.2	SPVVPF	14.8
645.4	400.3	SPVVPF	30.6
323.2	400.3	SPVVPF	14.8
645.4	388.2	SPVVPF	30.6
323.2	388.2	SPVVPF	14.8
645.4	383.2	SPVVPF	30.6
323.2	383.2	SPVVPF	14.8
645.4	362.2	SPVVPF	30.6
323.2	362.2	SPVVPF	14.8
645.4	355.2	SPVVPF	30.6

323.2	355.2	SPVVPF	14.8
645.4	345.2	SPVVPF	30.6
323.2	345.2	SPVVPF	14.8
645.4	301.2	SPVVPF	30.6
323.2	301.2	SPVVPF	14.8
645.4	289.1	SPVVPF	30.6
323.2	289.1	SPVVPF	14.8
645.4	284.2	SPVVPF	30.6
323.2	284.2	SPVVPF	14.8
645.4	263.1	SPVVPF	30.6
323.2	263.1	SPVVPF	14.8
645.4	256.2	SPVVPF	30.6
323.2	256.2	SPVVPF	14.8
645.4	246.1	SPVVPF	30.6
323.2	246.1	SPVVPF	14.8
645.4	202.1	SPVVPF	30.6
323.2	202.1	SPVVPF	14.8
645.4	192.1	SPVVPF	30.6
323.2	192.1	SPVVPF	14.8
645.4	185.1	SPVVPF	30.6
323.2	185.1	SPVVPF	14.8
645.4	166.1	SPVVPF	30.6
323.2	166.1	SPVVPF	14.8
645.4	157.1	SPVVPF	30.6
323.2	157.1	SPVVPF	14.8
645.4	149.1	SPVVPF	30.6
323.2	149.1	SPVVPF	14.8
645.4	105.1	SPVVPF	30.6
323.2	105.1	SPVVPF	14.8
645.4	88.0	SPVVPF	30.6

323.2	88.0	SPVVPF	14.8
645.4	60.0	SPVVPF	30.6
323.2	60.0	SPVVPF	14.8
1245.6	1170.5	TPEVDDEALEK	60.0
623.3	1170.5	TPEVDDEALEK	29.5
1245.6	1144.5	TPEVDDEALEK	60.0
623.3	1144.5	TPEVDDEALEK	29.5
1245.6	1116.5	TPEVDDEALEK	60.0
623.3	1116.5	TPEVDDEALEK	29.5
1245.6	1099.5	TPEVDDEALEK	60.0
623.3	1099.5	TPEVDDEALEK	29.5
1245.6	1073.5	TPEVDDEALEK	60.0
623.3	1073.5	TPEVDDEALEK	29.5
1245.6	1071484.0	TPEVDDEALEK	60.0
623.3	1071484.0	TPEVDDEALEK	29.5
1245.6	1047.5	TPEVDDEALEK	60.0
623.3	1047.5	TPEVDDEALEK	29.5
1245.6	1030.5	TPEVDDEALEK	60.0
623.3	1030.5	TPEVDDEALEK	29.5
1245.6	1127.5	TPEVDDEALEK	60.0
623.3	1127.5	TPEVDDEALEK	29.5
1245.6	987.5	TPEVDDEALEK	60.0
623.3	987.5	TPEVDDEALEK	29.5
1245.6	970.4	TPEVDDEALEK	60.0
623.3	970.4	TPEVDDEALEK	29.5
1245.6	944.4	TPEVDDEALEK	60.0
623.3	944.4	TPEVDDEALEK	29.5
1245.6	942.4	TPEVDDEALEK	60.0
623.3	942.4	TPEVDDEALEK	29.5
1245.6	918.4	TPEVDDEALEK	60.0

623.3	918.4	TPEVDDEALEK	29.5
1245.6	901.4	TPEVDDEALEK	60.0
623.3	901.4	TPEVDDEALEK	29.5
1245.6	874.4	TPEVDDEALEK	60.0
623.3	874.4	TPEVDDEALEK	29.5
1245.6	857.4	TPEVDDEALEK	60.0
623.3	857.4	TPEVDDEALEK	29.5
1245.6	845.4	TPEVDDEALEK	60.0
623.3	845.4	TPEVDDEALEK	29.5
1245.6	829.4	TPEVDDEALEK	60.0
623.3	829.4	TPEVDDEALEK	29.5
1245.6	819.4	TPEVDDEALEK	60.0
623.3	819.4	TPEVDDEALEK	29.5
1245.6	803.3	TPEVDDEALEK	60.0
623.3	803.3	TPEVDDEALEK	29.5
1245.6	802.3	TPEVDDEALEK	60.0
623.3	802.3	TPEVDDEALEK	29.5
1245.6	786.3	TPEVDDEALEK	60.0
623.3	786.3	TPEVDDEALEK	29.5
1245.6	758.3	TPEVDDEALEK	60.0
623.3	758.3	TPEVDDEALEK	29.5
1245.6	730.3	TPEVDDEALEK	60.0
623.3	730.3	TPEVDDEALEK	29.5
1245.6	704.3	TPEVDDEALEK	60.0
623.3	704.3	TPEVDDEALEK	29.5
1245.6	687.3	TPEVDDEALEK	60.0
623.3	687.3	TPEVDDEALEK	29.5
1245.6	674.3	TPEVDDEALEK	60.0
623.3	674.3	TPEVDDEALEK	29.5
1245.6	657.3	TPEVDDEALEK	60.0

623.3	657.3	TPEVDDEALEK	29.5
1245.6	629.3	TPEVDDEALEK	60.0
623.3	629.3	TPEVDDEALEK	29.5
1245.6	615.3	TPEVDDEALEK	60.0
623.3	615.3	TPEVDDEALEK	29.5
1245.6	589.3	TPEVDDEALEK	60.0
623.3	589.3	TPEVDDEALEK	29.5
1245.6	572.3	TPEVDDEALEK	60.0
623.3	572.3	TPEVDDEALEK	29.5
1245.6	559.3	TPEVDDEALEK	60.0
623.3	559.3	TPEVDDEALEK	29.5
1245.6	542.2	TPEVDDEALEK	60.0
623.3	542.2	TPEVDDEALEK	29.5
1245.6	514.3	TPEVDDEALEK	60.0
623.3	514.3	TPEVDDEALEK	29.5
1245.6	486.3	TPEVDDEALEK	60.0
623.3	486.3	TPEVDDEALEK	29.5
1245.6	460.3	TPEVDDEALEK	60.0
623.3	460.3	TPEVDDEALEK	29.5
1245.6	444.2	TPEVDDEALEK	60.0
623.3	444.2	TPEVDDEALEK	29.5
1245.6	443.3	TPEVDDEALEK	60.0
623.3	443.3	TPEVDDEALEK	29.5
1245.6	427.2	TPEVDDEALEK	60.0
623.3	427.2	TPEVDDEALEK	29.5
1245.6	415.2	TPEVDDEALEK	60.0
623.3	415.2	TPEVDDEALEK	29.5
1245.6	399.2	TPEVDDEALEK	60.0
623.3	399.2	TPEVDDEALEK	29.5
1245.6	389.2	TPEVDDEALEK	60.0

623.3	389.2	TPEVDDEALEK	29.5
1245.6	372.2	TPEVDDEALEK	60.0
623.3	372.2	TPEVDDEALEK	29.5
1245.6	345.2	TPEVDDEALEK	60.0
623.3	345.2	TPEVDDEALEK	29.5
1245.6	328.2	TPEVDDEALEK	60.0
623.3	328.2	TPEVDDEALEK	29.5
1245.6	302.1	TPEVDDEALEK	60.0
623.3	302.1	TPEVDDEALEK	29.5
1245.6	300.2	TPEVDDEALEK	60.0
623.3	300.2	TPEVDDEALEK	29.5
1245.6	276.2	TPEVDDEALEK	60.0
623.3	276.2	TPEVDDEALEK	29.5
1245.6	259.1	TPEVDDEALEK	60.0
623.3	259.1	TPEVDDEALEK	29.5
1245.6	216.1	TPEVDDEALEK	60.0
623.3	216.1	TPEVDDEALEK	29.5
1245.6	199.1	TPEVDDEALEK	60.0
623.3	199.1	TPEVDDEALEK	29.5
1245.6	173.1	TPEVDDEALEK	60.0
623.3	173.1	TPEVDDEALEK	29.5
1245.6	171.1	TPEVDDEALEK	60.0
623.3	171.1	TPEVDDEALEK	29.5
1245.6	147.1	TPEVDDEALEK	60.0
623.3	147.1	TPEVDDEALEK	29.5
1245.6	130.1	TPEVDDEALEK	60.0
623.3	130.1	TPEVDDEALEK	29.5
1245.6	119.1	TPEVDDEALEK	60.0
623.3	119.1	TPEVDDEALEK	29.5
1245.6	102.1	TPEVDDEALEK	60.0

623.3	102.1	TPEVDDEALEK	29.5
1245.6	74.1	TPEVDDEALEK	60.0
623.3	74.1	TPEVDDEALEK	29.5
708.4	633.4	TPVVVPP	33.7
354.7	633.4	TPVVVPP	16.4
708.4	610.4	TPVVVPP	33.7
354.7	610.4	TPVVVPP	16.4
708.4	607.4	TPVVVPP	33.7
354.7	607.4	TPVVVPP	16.4
708.4	593.4	TPVVVPP	33.7
354.7	593.4	TPVVVPP	16.4
708.4	590.4	TPVVVPP	33.7
354.7	590.4	TPVVVPP	16.4
708.4	565.4	TPVVVPP	33.7
354.7	565.4	TPVVVPP	16.4
708.4	536.3	TPVVVPP	33.7
354.7	536.3	TPVVVPP	16.4
708.4	513.3	TPVVVPP	33.7
354.7	513.3	TPVVVPP	16.4
708.4	510.3	TPVVVPP	33.7
354.7	510.3	TPVVVPP	16.4
708.4	496.3	TPVVVPP	33.7
354.7	496.3	TPVVVPP	16.4
708.4	493.3	TPVVVPP	33.7
354.7	493.3	TPVVVPP	16.4
708.4	468.3	TPVVVPP	33.7
354.7	468.3	TPVVVPP	16.4
708.4	437.2	TPVVVPP	33.7
354.7	437.2	TPVVVPP	16.4
708.4	414.3	TPVVVPP	33.7

354.7	414.3	TPVVVPP	16.4
708.4	411.3	TPVVVPP	33.7
354.7	411.3	TPVVVPP	16.4
708.4	397.2	TPVVVPP	33.7
354.7	397.2	TPVVVPP	16.4
708.4	394.2	TPVVVPP	33.7
354.7	394.2	TPVVVPP	16.4
708.4	369.3	TPVVVPP	33.7
354.7	369.3	TPVVVPP	16.4
708.4	338.2	TPVVVPP	33.7
354.7	338.2	TPVVVPP	16.4
708.4	315.2	TPVVVPP	33.7
354.7	315.2	TPVVVPP	16.4
708.4	312.2	TPVVVPP	33.7
354.7	312.2	TPVVVPP	16.4
708.4	298.2	TPVVVPP	33.7
354.7	298.2	TPVVVPP	16.4
708.4	295.2	TPVVVPP	33.7
354.7	295.2	TPVVVPP	16.4
708.4	270.2	TPVVVPP	33.7
354.7	270.2	TPVVVPP	16.4
708.4	239.1	TPVVVPP	33.7
354.7	239.1	TPVVVPP	16.4
708.4	216.1	TPVVVPP	33.7
354.7	216.1	TPVVVPP	16.4
708.4	213.1	TPVVVPP	33.7
354.7	213.1	TPVVVPP	16.4
708.4	199.1	TPVVVPP	33.7
354.7	199.1	TPVVVPP	16.4
708.4	196.1	TPVVVPP	33.7

354.7	196.1	TPVVVPP	16.4
708.4	171.1	TPVVVPP	33.7
354.7	171.1	TPVVVPP	16.4
708.4	142.1	TPVVVPP	33.7
354.7	142.1	TPVVVPP	16.4
708.4	119.1	TPVVVPP	33.7
354.7	119.1	TPVVVPP	16.4
708.4	116.1	TPVVVPP	33.7
354.7	116.1	TPVVVPP	16.4
708.4	102.1	TPVVVPP	33.7
354.7	102.1	TPVVVPP	16.4
708.4	99.0	TPVVVPP	33.7
354.7	99.0	TPVVVPP	16.4
708.4	74.1	TPVVVPP	33.7
354.7	74.1	TPVVVPP	16.4
828.4	1223.5	TQMVDEEIMEKFR	39.6
828.4	1222.5	TQMVDEEIMEKFR	39.6
828.4	1206.5	TQMVDEEIMEKFR	39.6
828.4	1196.6	TQMVDEEIMEKFR	39.6
828.4	1179.5	TQMVDEEIMEKFR	39.6
828.4	1178.5	TQMVDEEIMEKFR	39.6
828.4	1107.5	TQMVDEEIMEKFR	39.6
828.4	1094.5	TQMVDEEIMEKFR	39.6
828.4	1081535.0	TQMVDEEIMEKFR	39.6
828.4	1077.5	TQMVDEEIMEKFR	39.6
828.4	1064.5	TQMVDEEIMEKFR	39.6
828.4	1049.5	TQMVDEEIMEKFR	39.6
828.4	978.5	TQMVDEEIMEKFR	39.6
828.4	963.4	TQMVDEEIMEKFR	39.6
828.4	952.5	TQMVDEEIMEKFR	39.6

828.4	946.4	TQMVDEEIMEKFR	39.6
828.4	935.5	TQMVDEEIMEKFR	39.6
828.4	918.4	TQMVDEEIMEKFR	39.6
828.4	850.4	TQMVDEEIMEKFR	39.6
828.4	849.4	TQMVDEEIMEKFR	39.6
828.4	833.3	TQMVDEEIMEKFR	39.6
828.4	823.4	TQMVDEEIMEKFR	39.6
828.4	806.4	TQMVDEEIMEKFR	39.6
828.4	805.3	TQMVDEEIMEKFR	39.6
828.4	736.3	TQMVDEEIMEKFR	39.6
828.4	721.3	TQMVDEEIMEKFR	39.6
828.4	710.4	TQMVDEEIMEKFR	39.6
828.4	704.3	TQMVDEEIMEKFR	39.6
828.4	693.3	TQMVDEEIMEKFR	39.6
828.4	676.3	TQMVDEEIMEKFR	39.6
828.4	605.3	TQMVDEEIMEKFR	39.6
828.4	592.3	TQMVDEEIMEKFR	39.6
828.4	579.3	TQMVDEEIMEKFR	39.6
828.4	575.2	TQMVDEEIMEKFR	39.6
828.4	562.3	TQMVDEEIMEKFR	39.6
828.4	547.3	TQMVDEEIMEKFR	39.6
828.4	477.2	TQMVDEEIMEKFR	39.6
828.4	476.3	TQMVDEEIMEKFR	39.6
828.4	460.2	TQMVDEEIMEKFR	39.6
828.4	450.3	TQMVDEEIMEKFR	39.6
828.4	433.3	TQMVDEEIMEKFR	39.6
828.4	432.2	TQMVDEEIMEKFR	39.6
828.4	378.2	TQMVDEEIMEKFR	39.6
828.4	361.2	TQMVDEEIMEKFR	39.6
828.4	348.2	TQMVDEEIMEKFR	39.6

828.4	333.2	TQMVDEEIMEKFR	39.6
828.4	322.2	TQMVDEEIMEKFR	39.6
828.4	305.2	TQMVDEEIMEKFR	39.6
828.4	247.1	TQMVDEEIMEKFR	39.6
828.4	230.1	TQMVDEEIMEKFR	39.6
828.4	202.1	TQMVDEEIMEKFR	39.6
828.4	201.1	TQMVDEEIMEKFR	39.6
828.4	175.1	TQMVDEEIMEKFR	39.6
828.4	158.1	TQMVDEEIMEKFR	39.6
828.4	119.1	TQMVDEEIMEKFR	39.6
828.4	102.1	TQMVDEEIMEKFR	39.6
828.4	74.1	TQMVDEEIMEKFR	39.6
189.1	117.1	VA	10.0
189.1	116.0	VA	10.0
189.1	100.1	VA	10.0
189.1	90.1	VA	10.0
189.1	73.0	VA	10.0
189.1	72.1	VA	10.0
696.3	623.2	VAGTWY	33.1
696.3	597.3	VAGTWY	33.1
696.3	580.2	VAGTWY	33.1
696.3	552.2	VAGTWY	33.1
696.3	532.3	VAGTWY	33.1
696.3	526.2	VAGTWY	33.1
696.3	515.3	VAGTWY	33.1
696.3	509.2	VAGTWY	33.1
696.3	495.2	VAGTWY	33.1
696.3	487.3	VAGTWY	33.1
696.3	469.2	VAGTWY	33.1
696.3	452.2	VAGTWY	33.1

696.3	394.1	VAGTWY	33.1
696.3	368.2	VAGTWY	33.1
696.3	351.1	VAGTWY	33.1
696.3	346.2	VAGTWY	33.1
696.3	329.2	VAGTWY	33.1
696.3	301.2	VAGTWY	33.1
696.3	245.2	VAGTWY	33.1
696.3	228.1	VAGTWY	33.1
696.3	208.1	VAGTWY	33.1
696.3	200.1	VAGTWY	33.1
696.3	188.1	VAGTWY	33.1
696.3	182.1	VAGTWY	33.1
696.3	171.1	VAGTWY	33.1
696.3	165.1	VAGTWY	33.1
696.3	143.1	VAGTWY	33.1
696.3	117.1	VAGTWY	33.1
696.3	100.1	VAGTWY	33.1
696.3	72.1	VAGTWY	33.1
278.1	205.0	VC[CAM]	12.6
278.1	179.0	VC[CAM]	12.6
278.1	162.0	VC[CAM]	12.6
278.1	117.1	VC[CAM]	12.6
278.1	100.1	VC[CAM]	12.6
278.1	72.1	VC[CAM]	12.6
385.2	312.2	VLGP	17.9
385.2	287.2	VLGP	17.9
385.2	286.2	VLGP	17.9
385.2	270.2	VLGP	17.9
385.2	269.2	VLGP	17.9
385.2	242.2	VLGP	17.9

385.2	230.2	VLGP	17.9
385.2	213.2	VLGP	17.9
385.2	199.1	VLGP	17.9
385.2	185.2	VLGP	17.9
385.2	173.1	VLGP	17.9
385.2	156.1	VLGP	17.9
385.2	142.1	VLGP	17.9
385.2	117.1	VLGP	17.9
385.2	116.1	VLGP	17.9
385.2	100.1	VLGP	17.9
385.2	99.0	VLGP	17.9
385.2	72.1	VLGP	17.9
1065.6	992.5	VLVLDTDYK	51.2
1065.6	966.5	VLVLDTDYK	51.2
1065.6	949.5	VLVLDTDYK	51.2
1065.6	936.5	VLVLDTDYK	51.2
1065.6	919.5	VLVLDTDYK	51.2
1065.6	891.5	VLVLDTDYK	51.2
1065.6	879.4	VLVLDTDYK	51.2
1065.6	853.4	VLVLDTDYK	51.2
1065.6	836.4	VLVLDTDYK	51.2
1065.6	780.3	VLVLDTDYK	51.2
1065.6	773.4	VLVLDTDYK	51.2
1065.6	756.4	VLVLDTDYK	51.2
1065.6	754.4	VLVLDTDYK	51.2
1065.6	737.3	VLVLDTDYK	51.2
1065.6	728.4	VLVLDTDYK	51.2
1065.6	667.3	VLVLDTDYK	51.2
1065.6	658.4	VLVLDTDYK	51.2
1065.6	641.4	VLVLDTDYK	51.2

1065.6	641.3	VLVLDTDYK	51.2
1065.6	624.3	VLVLDTDYK	51.2
1065.6	613.4	VLVLDTDYK	51.2
1065.6	557.4	VLVLDTDYK	51.2
1065.6	552.2	VLVLDTDYK	51.2
1065.6	540.3	VLVLDTDYK	51.2
1065.6	526.3	VLVLDTDYK	51.2
1065.6	512.3	VLVLDTDYK	51.2
1065.6	509.2	VLVLDTDYK	51.2
1065.6	451.2	VLVLDTDYK	51.2
1065.6	442.3	VLVLDTDYK	51.2
1065.6	425.3	VLVLDTDYK	51.2
1065.6	425.2	VLVLDTDYK	51.2
1065.6	408.2	VLVLDTDYK	51.2
1065.6	397.3	VLVLDTDYK	51.2
1065.6	336.2	VLVLDTDYK	51.2
1065.6	329.3	VLVLDTDYK	51.2
1065.6	312.2	VLVLDTDYK	51.2
1065.6	310.2	VLVLDTDYK	51.2
1065.6	293.2	VLVLDTDYK	51.2
1065.6	284.2	VLVLDTDYK	51.2
1065.6	230.2	VLVLDTDYK	51.2
1065.6	213.2	VLVLDTDYK	51.2
1065.6	185.2	VLVLDTDYK	51.2
1065.6	173.1	VLVLDTDYK	51.2
1065.6	147.1	VLVLDTDYK	51.2
1065.6	130.1	VLVLDTDYK	51.2
1065.6	117.1	VLVLDTDYK	51.2
1065.6	100.1	VLVLDTDYK	51.2
1065.6	72.1	VLVLDTDYK	51.2

215.1	142.1	VP	10.0
215.1	117.1	VP	10.0
215.1	116.1	VP	10.0
215.1	100.1	VP	10.0
215.1	99.0	VP	10.0
215.1	72.1	VP	10.0
742.4	669.3	VPGEIVE	35.4
742.4	643.3	VPGEIVE	35.4
742.4	626.3	VPGEIVE	35.4
742.4	612.4	VPGEIVE	35.4
742.4	595.3	VPGEIVE	35.4
742.4	572.3	VPGEIVE	35.4
742.4	567.4	VPGEIVE	35.4
742.4	546.3	VPGEIVE	35.4
742.4	529.3	VPGEIVE	35.4
742.4	515.2	VPGEIVE	35.4
742.4	513.3	VPGEIVE	35.4
742.4	496.3	VPGEIVE	35.4
742.4	489.3	VPGEIVE	35.4
742.4	472.2	VPGEIVE	35.4
742.4	468.3	VPGEIVE	35.4
742.4	400.2	VPGEIVE	35.4
742.4	386.2	VPGEIVE	35.4
742.4	383.2	VPGEIVE	35.4
742.4	360.2	VPGEIVE	35.4
742.4	355.2	VPGEIVE	35.4
742.4	343.2	VPGEIVE	35.4
742.4	273.1	VPGEIVE	35.4
742.4	271.2	VPGEIVE	35.4
742.4	254.2	VPGEIVE	35.4

742.4	247.1	VPGEIVE	35.4
742.4	230.1	VPGEIVE	35.4
742.4	226.2	VPGEIVE	35.4
742.4	214.2	VPGEIVE	35.4
742.4	197.1	VPGEIVE	35.4
742.4	174.0	VPGEIVE	35.4
742.4	169.1	VPGEIVE	35.4
742.4	148.1	VPGEIVE	35.4
742.4	131.0	VPGEIVE	35.4
742.4	117.1	VPGEIVE	35.4
742.4	100.1	VPGEIVE	35.4
742.4	72.1	VPGEIVE	35.4
645.4	572.3	VPITP	30.6
645.4	546.3	VPITP	30.6
645.4	529.3	VPITP	30.6
645.4	525.3	VPITP	30.6
645.4	508.3	VPITP	30.6
645.4	480.3	VPITP	30.6
645.4	475.2	VPITP	30.6
645.4	449.3	VPITP	30.6
645.4	432.2	VPITP	30.6
645.4	428.3	VPITP	30.6
645.4	411.3	VPITP	30.6
645.4	383.3	VPITP	30.6
645.4	362.2	VPITP	30.6
645.4	336.2	VPITP	30.6
645.4	327.2	VPITP	30.6
645.4	319.1	VPITP	30.6
645.4	310.2	VPITP	30.6
645.4	282.2	VPITP	30.6

645.4	261.1	VPITP	30.6
645.4	235.1	VPITP	30.6
645.4	218.1	VPITP	30.6
645.4	214.2	VPITP	30.6
645.4	197.1	VPITP	30.6
645.4	169.1	VPITP	30.6
645.4	164.1	VPITP	30.6
645.4	138.1	VPITP	30.6
645.4	121.0	VPITP	30.6
645.4	117.1	VPITP	30.6
645.4	100.1	VPITP	30.6
645.4	72.1	VPITP	30.6
740.5	667.4	VPITPTL	35.3
740.5	641.4	VPITPTL	35.3
740.5	626.4	VPITPTL	35.3
740.5	624.4	VPITPTL	35.3
740.5	609.4	VPITPTL	35.3
740.5	581.4	VPITPTL	35.3
740.5	570.3	VPITPTL	35.3
740.5	544.3	VPITPTL	35.3
740.5	527.3	VPITPTL	35.3
740.5	525.3	VPITPTL	35.3
740.5	508.3	VPITPTL	35.3
740.5	480.3	VPITPTL	35.3
740.5	457.2	VPITPTL	35.3
740.5	431.3	VPITPTL	35.3
740.5	428.3	VPITPTL	35.3
740.5	414.2	VPITPTL	35.3
740.5	411.3	VPITPTL	35.3
740.5	383.3	VPITPTL	35.3

740.5	356.2	VPITPTL	35.3
740.5	330.2	VPITPTL	35.3
740.5	327.2	VPITPTL	35.3
740.5	313.2	VPITPTL	35.3
740.5	310.2	VPITPTL	35.3
740.5	282.2	VPITPTL	35.3
740.5	259.1	VPITPTL	35.3
740.5	233.2	VPITPTL	35.3
740.5	216.1	VPITPTL	35.3
740.5	214.2	VPITPTL	35.3
740.5	197.1	VPITPTL	35.3
740.5	169.1	VPITPTL	35.3
740.5	158.1	VPITPTL	35.3
740.5	132.1	VPITPTL	35.3
740.5	117.1	VPITPTL	35.3
740.5	115.1	VPITPTL	35.3
740.5	100.1	VPITPTL	35.3
740.5	72.1	VPITPTL	35.3
328.2	255.1	VPL	15.1
328.2	229.2	VPL	15.1
328.2	214.2	VPL	15.1
328.2	212.1	VPL	15.1
328.2	197.1	VPL	15.1
328.2	169.1	VPL	15.1
328.2	158.1	VPL	15.1
328.2	132.1	VPL	15.1
328.2	117.1	VPL	15.1
328.2	115.1	VPL	15.1
328.2	100.1	VPL	15.1
328.2	72.1	VPL	15.1

274.2	201.1	VR	12.4
274.2	175.1	VR	12.4
274.2	158.1	VR	12.4
274.2	117.1	VR	12.4
274.2	100.1	VR	12.4
274.2	72.1	VR	12.4
276.1	204.1	WA	12.5
276.1	187.1	WA	12.5
276.1	159.1	WA	12.5
276.1	116.0	WA	12.5
276.1	90.1	WA	12.5
276.1	73.0	WA	12.5
365.1	205.0	WC[CAM]	16.9
365.1	204.1	WC[CAM]	16.9
365.1	187.1	WC[CAM]	16.9
365.1	179.0	WC[CAM]	16.9
365.1	162.0	WC[CAM]	16.9
365.1	159.1	WC[CAM]	16.9
320.1	204.1	WD	14.7
320.1	187.1	WD	14.7
320.1	160.0	WD	14.7
320.1	159.1	WD	14.7
320.1	134.0	WD	14.7
320.1	117.0	WD	14.7
334.1	204.1	WE	15.4
334.1	187.1	WE	15.4
334.1	174.0	WE	15.4
334.1	159.1	WE	15.4
334.1	148.1	WE	15.4
334.1	131.0	WE	15.4

352.2	204.1	WF	16.3
352.2	192.1	WF	16.3
352.2	187.1	WF	16.3
352.2	166.1	WF	16.3
352.2	159.1	WF	16.3
352.2	149.1	WF	16.3
262.1	204.1	WG	11.8
262.1	187.1	WG	11.8
262.1	159.1	WG	11.8
262.1	102.0	WG	11.8
262.1	76.0	WG	11.8
262.1	59.0	WG	11.8
318.2	204.1	WI	14.6
318.2	187.1	WI	14.6
318.2	159.1	WI	14.6
318.2	158.1	WI	14.6
318.2	132.1	WI	14.6
318.2	115.1	WI	14.6
543.3	445.3	WIQP	25.6
543.3	428.2	WIQP	25.6
543.3	400.2	WIQP	25.6
543.3	383.2	WIQP	25.6
543.3	357.2	WIQP	25.6
543.3	340.2	WIQP	25.6
543.3	317.2	WIQP	25.6
543.3	300.2	WIQP	25.6
543.3	272.2	WIQP	25.6
543.3	270.1	WIQP	25.6
543.3	244.1	WIQP	25.6
543.3	227.1	WIQP	25.6

543.3	204.1	WIQP	25.6
543.3	187.1	WIQP	25.6
543.3	159.1	WIQP	25.6
543.3	142.1	WIQP	25.6
543.3	116.1	WIQP	25.6
543.3	99.0	WIQP	25.6
333.2	204.1	WK	15.3
333.2	187.1	WK	15.3
333.2	173.1	WK	15.3
333.2	159.1	WK	15.3
333.2	147.1	WK	15.3
333.2	130.1	WK	15.3
318.2	204.1	WL	14.6
318.2	187.1	WL	14.6
318.2	159.1	WL	14.6
318.2	158.1	WL	14.6
318.2	132.1	WL	14.6
318.2	115.1	WL	14.6
838.5	724.4	WLAHKAL	40.1
838.5	707.4	WLAHKAL	40.1
838.5	679.4	WLAHKAL	40.1
838.5	678.4	WLAHKAL	40.1
838.5	653.4	WLAHKAL	40.1
838.5	652.4	WLAHKAL	40.1
838.5	636.4	WLAHKAL	40.1
838.5	635.4	WLAHKAL	40.1
838.5	608.4	WLAHKAL	40.1
838.5	565.3	WLAHKAL	40.1
838.5	539.3	WLAHKAL	40.1
838.5	525.3	WLAHKAL	40.1

838.5	522.3	WLAHKAL	40.1
838.5	508.3	WLAHKAL	40.1
838.5	494.3	WLAHKAL	40.1
838.5	480.3	WLAHKAL	40.1
838.5	468.3	WLAHKAL	40.1
838.5	451.3	WLAHKAL	40.1
838.5	388.2	WLAHKAL	40.1
838.5	371.2	WLAHKAL	40.1
838.5	357.2	WLAHKAL	40.1
838.5	343.2	WLAHKAL	40.1
838.5	331.2	WLAHKAL	40.1
838.5	317.2	WLAHKAL	40.1
838.5	314.2	WLAHKAL	40.1
838.5	300.2	WLAHKAL	40.1
838.5	272.2	WLAHKAL	40.1
838.5	229.1	WLAHKAL	40.1
838.5	204.1	WLAHKAL	40.1
838.5	203.1	WLAHKAL	40.1
838.5	187.1	WLAHKAL	40.1
838.5	186.1	WLAHKAL	40.1
838.5	159.1	WLAHKAL	40.1
838.5	158.1	WLAHKAL	40.1
838.5	132.1	WLAHKAL	40.1
838.5	115.1	WLAHKAL	40.1
849.9	1217.6	WLAHKALC[CAM]SEKLDQ	40.6
849.9	1213.6	WLAHKALC[CAM]SEKLDQ	40.6
849.9	1196.6	WLAHKALC[CAM]SEKLDQ	40.6
849.9	1174.6	WLAHKALC[CAM]SEKLDQ	40.6
849.9	1168.6	WLAHKALC[CAM]SEKLDQ	40.6
849.9	1089.5	WLAHKALC[CAM]SEKLDQ	40.6

849.9	1084.6	WLAHKALC[CAM]SEKLDQ	40.6
849.9	1067.5	WLAHKALC[CAM]SEKLDQ	40.6
849.9	1046.5	WLAHKALC[CAM]SEKLDQ	40.6
849.9	1039.6	WLAHKALC[CAM]SEKLDQ	40.6
849.9	1018.5	WLAHKALC[CAM]SEKLDQ	40.6
849.9	1063.5	WLAHKALC[CAM]SEKLDQ	40.6
849.9	997.5	WLAHKALC[CAM]SEKLDQ	40.6
849.9	992.5	WLAHKALC[CAM]SEKLDQ	40.6
849.9	980.5	WLAHKALC[CAM]SEKLDQ	40.6
849.9	975.4	WLAHKALC[CAM]SEKLDQ	40.6
849.9	952.5	WLAHKALC[CAM]SEKLDQ	40.6
849.9	905.4	WLAHKALC[CAM]SEKLDQ	40.6
849.9	879.4	WLAHKALC[CAM]SEKLDQ	40.6
849.9	862.4	WLAHKALC[CAM]SEKLDQ	40.6
849.9	837.5	WLAHKALC[CAM]SEKLDQ	40.6
849.9	820.5	WLAHKALC[CAM]SEKLDQ	40.6
849.9	792.5	WLAHKALC[CAM]SEKLDQ	40.6
849.9	745.3	WLAHKALC[CAM]SEKLDQ	40.6
849.9	724.4	WLAHKALC[CAM]SEKLDQ	40.6
849.9	719.4	WLAHKALC[CAM]SEKLDQ	40.6
849.9	707.4	WLAHKALC[CAM]SEKLDQ	40.6
849.9	702.3	WLAHKALC[CAM]SEKLDQ	40.6
849.9	679.4	WLAHKALC[CAM]SEKLDQ	40.6
849.9	658.3	WLAHKALC[CAM]SEKLDQ	40.6
849.9	653.4	WLAHKALC[CAM]SEKLDQ	40.6
849.9	636.4	WLAHKALC[CAM]SEKLDQ	40.6
849.9	632.3	WLAHKALC[CAM]SEKLDQ	40.6
849.9	615.3	WLAHKALC[CAM]SEKLDQ	40.6
849.9	608.4	WLAHKALC[CAM]SEKLDQ	40.6
849.9	529.3	WLAHKALC[CAM]SEKLDQ	40.6

849.9	525.3	WLAHKALC[CAM]SEKLDQ	40.6
849.9	508.3	WLAHKALC[CAM]SEKLDQ	40.6
849.9	503.3	WLAHKALC[CAM]SEKLDQ	40.6
849.9	486.3	WLAHKALC[CAM]SEKLDQ	40.6
849.9	480.3	WLAHKALC[CAM]SEKLDQ	40.6
849.9	401.2	WLAHKALC[CAM]SEKLDQ	40.6
849.9	388.2	WLAHKALC[CAM]SEKLDQ	40.6
849.9	375.2	WLAHKALC[CAM]SEKLDQ	40.6
849.9	371.2	WLAHKALC[CAM]SEKLDQ	40.6
849.9	358.2	WLAHKALC[CAM]SEKLDQ	40.6
849.9	343.2	WLAHKALC[CAM]SEKLDQ	40.6
849.9	317.2	WLAHKALC[CAM]SEKLDQ	40.6
849.9	300.2	WLAHKALC[CAM]SEKLDQ	40.6
849.9	288.1	WLAHKALC[CAM]SEKLDQ	40.6
849.9	272.2	WLAHKALC[CAM]SEKLDQ	40.6
849.9	262.1	WLAHKALC[CAM]SEKLDQ	40.6
849.9	245.1	WLAHKALC[CAM]SEKLDQ	40.6
849.9	204.1	WLAHKALC[CAM]SEKLDQ	40.6
849.9	187.1	WLAHKALC[CAM]SEKLDQ	40.6
849.9	173.1	WLAHKALC[CAM]SEKLDQ	40.6
849.9	159.1	WLAHKALC[CAM]SEKLDQ	40.6
849.9	147.1	WLAHKALC[CAM]SEKLDQ	40.6
849.9	130.1	WLAHKALC[CAM]SEKLDQ	40.6
849.9	1191.6	WLAHKALC[CAM]SEKLDQy	40.6
336.1	204.1	WM	15.5
336.1	187.1	WM	15.5
336.1	176.0	WM	15.5
336.1	159.1	WM	15.5
336.1	150.1	WM	15.5
336.1	133.0	WM	15.5

319.1	204.1	WN	14.6
319.1	187.1	WN	14.6
319.1	159.1	WN	14.6
319.1	159.0	WN	14.6
319.1	133.1	WN	14.6
319.1	116.0	WN	14.6
302.2	204.1	WP	13.8
302.2	187.1	WP	13.8
302.2	159.1	WP	13.8
302.2	142.1	WP	13.8
302.2	116.1	WP	13.8
302.2	99.0	WP	13.8
449.2	301.2	WPF	21.0
449.2	289.1	WPF	21.0
449.2	284.1	WPF	21.0
449.2	263.1	WPF	21.0
449.2	256.1	WPF	21.0
449.2	246.1	WPF	21.0
449.2	204.1	WPF	21.0
449.2	192.1	WPF	21.0
449.2	187.1	WPF	21.0
449.2	166.1	WPF	21.0
449.2	159.1	WPF	21.0
449.2	149.1	WPF	21.0
700.3	602.3	WPFPGP	33.3
700.3	585.3	WPFPGP	33.3
700.3	557.3	WPFPGP	33.3
700.3	545.3	WPFPGP	33.3
700.3	540.2	WPFPGP	33.3
700.3	528.3	WPFPGP	33.3

700.3	514.3	WPFPGP	33.3
700.3	500.3	WPFPGP	33.3
700.3	497.2	WPFPGP	33.3
700.3	448.2	WPFPGP	33.3
700.3	443.2	WPFPGP	33.3
700.3	431.2	WPFPGP	33.3
700.3	417.2	WPFPGP	33.3
700.3	403.2	WPFPGP	33.3
700.3	400.2	WPFPGP	33.3
700.3	301.2	WPFPGP	33.3
700.3	296.1	WPFPGP	33.3
700.3	284.1	WPFPGP	33.3
700.3	270.1	WPFPGP	33.3
700.3	256.1	WPFPGP	33.3
700.3	253.1	WPFPGP	33.3
700.3	204.1	WPFPGP	33.3
700.3	199.1	WPFPGP	33.3
700.3	187.1	WPFPGP	33.3
700.3	173.1	WPFPGP	33.3
700.3	159.1	WPFPGP	33.3
700.3	156.1	WPFPGP	33.3
700.3	142.1	WPFPGP	33.3
700.3	116.1	WPFPGP	33.3
700.3	99.0	WPFPGP	33.3
359.2	301.2	WPG	16.6
359.2	284.1	WPG	16.6
359.2	256.1	WPG	16.6
359.2	204.1	WPG	16.6
359.2	199.1	WPG	16.6
359.2	187.1	WPG	16.6

359.2	173.1	WPG	16.6
359.2	159.1	WPG	16.6
359.2	156.1	WPG	16.6
359.2	102.0	WPG	16.6
359.2	76.0	WPG	16.6
359.2	59.0	WPG	16.6
415.2	301.2	WPL	19.3
415.2	284.1	WPL	19.3
415.2	256.1	WPL	19.3
415.2	255.1	WPL	19.3
415.2	229.2	WPL	19.3
415.2	212.1	WPL	19.3
415.2	204.1	WPL	19.3
415.2	187.1	WPL	19.3
415.2	159.1	WPL	19.3
415.2	158.1	WPL	19.3
415.2	132.1	WPL	19.3
415.2	115.1	WPL	19.3
403.2	301.2	WPT	18.8
403.2	284.1	WPT	18.8
403.2	256.1	WPT	18.8
403.2	243.1	WPT	18.8
403.2	217.1	WPT	18.8
403.2	204.1	WPT	18.8
403.2	200.1	WPT	18.8
403.2	187.1	WPT	18.8
403.2	159.1	WPT	18.8
403.2	146.0	WPT	18.8
403.2	120.1	WPT	18.8
403.2	103.0	WPT	18.8

774.4	626.3	WPVEPF	36.9
774.4	614.3	WPVEPF	36.9
774.4	609.3	WPVEPF	36.9
774.4	588.3	WPVEPF	36.9
774.4	581.3	WPVEPF	36.9
774.4	571.3	WPVEPF	36.9
774.4	529.3	WPVEPF	36.9
774.4	517.2	WPVEPF	36.9
774.4	512.3	WPVEPF	36.9
774.4	491.3	WPVEPF	36.9
774.4	484.3	WPVEPF	36.9
774.4	474.2	WPVEPF	36.9
774.4	418.2	WPVEPF	36.9
774.4	400.2	WPVEPF	36.9
774.4	392.2	WPVEPF	36.9
774.4	383.2	WPVEPF	36.9
774.4	375.2	WPVEPF	36.9
774.4	355.2	WPVEPF	36.9
774.4	301.2	WPVEPF	36.9
774.4	289.1	WPVEPF	36.9
774.4	284.1	WPVEPF	36.9
774.4	263.1	WPVEPF	36.9
774.4	256.1	WPVEPF	36.9
774.4	246.1	WPVEPF	36.9
774.4	204.1	WPVEPF	36.9
774.4	192.1	WPVEPF	36.9
774.4	187.1	WPVEPF	36.9
774.4	166.1	WPVEPF	36.9
774.4	159.1	WPVEPF	36.9
774.4	149.1	WPVEPF	36.9

488.2	328.1	WPW	22.9
488.2	302.2	WPW	22.9
488.2	301.2	WPW	22.9
488.2	285.1	WPW	22.9
488.2	284.1	WPW	22.9
488.2	256.1	WPW	22.9
488.2	231.1	WPW	22.9
488.2	205.1	WPW	22.9
488.2	204.1	WPW	22.9
488.2	188.1	WPW	22.9
488.2	187.1	WPW	22.9
488.2	159.1	WPW	22.9
333.2	204.1	WQ	15.3
333.2	187.1	WQ	15.3
333.2	173.1	WQ	15.3
333.2	159.1	WQ	15.3
333.2	147.1	WQ	15.3
333.2	130.1	WQ	15.3
361.2	204.1	WR	16.7
361.2	201.1	WR	16.7
361.2	187.1	WR	16.7
361.2	175.1	WR	16.7
361.2	159.1	WR	16.7
361.2	158.1	WR	16.7
292.1	204.1	WS	13.3
292.1	187.1	WS	13.3
292.1	159.1	WS	13.3
292.1	132.0	WS	13.3
292.1	106.1	WS	13.3
292.1	89.0	WS	13.3

349.2	291.1	WSG	16.1
349.2	274.1	WSG	16.1
349.2	246.1	WSG	16.1
349.2	204.1	WSG	16.1
349.2	189.1	WSG	16.1
349.2	187.1	WSG	16.1
349.2	163.1	WSG	16.1
349.2	159.1	WSG	16.1
349.2	146.0	WSG	16.1
349.2	102.0	WSG	16.1
349.2	76.0	WSG	16.1
349.2	59.0	WSG	16.1
306.1	204.1	WT	14.0
306.1	187.1	WT	14.0
306.1	159.1	WT	14.0
306.1	146.0	WT	14.0
306.1	120.1	WT	14.0
306.1	103.0	WT	14.0
304.2	204.1	WV	13.9
304.2	187.1	WV	13.9
304.2	159.1	WV	13.9
304.2	144.1	WV	13.9
304.2	118.1	WV	13.9
304.2	101.1	WV	13.9
391.2	231.1	WW	18.2
391.2	205.1	WW	18.2
391.2	204.1	WW	18.2
391.2	188.1	WW	18.2
391.2	187.1	WW	18.2
391.2	159.1	WW	18.2

368.2	208.1	WY	17.0
368.2	204.1	WY	17.0
368.2	187.1	WY	17.0
368.2	182.1	WY	17.0
368.2	165.1	WY	17.0
368.2	159.1	WY	17.0
279.1	181.1	YP	12.7
279.1	164.1	YP	12.7
279.1	142.1	YP	12.7
279.1	136.1	YP	12.7
279.1	116.1	YP	12.7
279.1	99.0	YP	12.7
983.5	868.5	YPEPGPIPN	47.2
983.5	851.4	YPEPGPIPN	47.2
983.5	846.4	YPEPGPIPN	47.2
983.5	823.4	YPEPGPIPN	47.2
983.5	820.4	YPEPGPIPN	47.2
983.5	803.4	YPEPGPIPN	47.2
983.5	771.4	YPEPGPIPN	47.2
983.5	754.4	YPEPGPIPN	47.2
983.5	749.3	YPEPGPIPN	47.2
983.5	726.4	YPEPGPIPN	47.2
983.5	723.4	YPEPGPIPN	47.2
983.5	706.3	YPEPGPIPN	47.2
983.5	658.3	YPEPGPIPN	47.2
983.5	641.3	YPEPGPIPN	47.2
983.5	620.3	YPEPGPIPN	47.2
983.5	613.3	YPEPGPIPN	47.2
983.5	594.3	YPEPGPIPN	47.2
983.5	577.3	YPEPGPIPN	47.2

983.5	561.3	YPEPGPIPN	47.2
983.5	544.2	YPEPGPIPN	47.2
983.5	523.3	YPEPGPIPN	47.2
983.5	516.2	YPEPGPIPN	47.2
983.5	504.2	YPEPGPIPN	47.2
983.5	497.3	YPEPGPIPN	47.2
983.5	487.2	YPEPGPIPN	47.2
983.5	480.2	YPEPGPIPN	47.2
983.5	466.2	YPEPGPIPN	47.2
983.5	459.2	YPEPGPIPN	47.2
983.5	440.3	YPEPGPIPN	47.2
983.5	423.2	YPEPGPIPN	47.2
983.5	407.2	YPEPGPIPN	47.2
983.5	390.2	YPEPGPIPN	47.2
983.5	369.2	YPEPGPIPN	47.2
983.5	362.2	YPEPGPIPN	47.2
983.5	343.2	YPEPGPIPN	47.2
983.5	326.2	YPEPGPIPN	47.2
983.5	278.2	YPEPGPIPN	47.2
983.5	261.1	YPEPGPIPN	47.2
983.5	256.1	YPEPGPIPN	47.2
983.5	233.1	YPEPGPIPN	47.2
983.5	230.1	YPEPGPIPN	47.2
983.5	213.1	YPEPGPIPN	47.2
983.5	181.1	YPEPGPIPN	47.2
983.5	164.1	YPEPGPIPN	47.2
983.5	159.0	YPEPGPIPN	47.2
983.5	136.1	YPEPGPIPN	47.2
983.5	133.1	YPEPGPIPN	47.2
983.5	116.0	YPEPGPIPN	47.2

442.2	305.1	YPY	20.7
442.2	279.1	YPY	20.7
442.2	278.2	YPY	20.7
442.2	262.1	YPY	20.7
442.2	261.1	YPY	20.7
442.2	233.1	YPY	20.7
442.2	208.1	YPY	20.7
442.2	182.1	YPY	20.7
442.2	181.1	YPY	20.7
442.2	165.1	YPY	20.7
442.2	164.1	YPY	20.7
442.2	136.1	YPY	20.7
605.3	468.2	YPYY	28.7
605.3	442.2	YPYY	28.7
605.3	441.2	YPYY	28.7
605.3	425.2	YPYY	28.7
605.3	424.2	YPYY	28.7
605.3	396.2	YPYY	28.7
605.3	371.1	YPYY	28.7
605.3	345.1	YPYY	28.7
605.3	328.1	YPYY	28.7
605.3	278.2	YPYY	28.7
605.3	261.1	YPYY	28.7
605.3	233.1	YPYY	28.7
605.3	208.1	YPYY	28.7
605.3	182.1	YPYY	28.7
605.3	181.1	YPYY	28.7
605.3	165.1	YPYY	28.7
605.3	164.1	YPYY	28.7
605.3	136.1	YPYY	28.7

37

Table S 3: MS/MS parameters used for the quantification of candidate peptides.

Common settings for all peptides: EP 10V, Dwell time 15 ms

Q1	Peptide	Q3	CE [V]	DP [V]	CXP [V]
334.0	APF	263.1	21	36	30
334.0	APF	169.0	19	36	20
334.0	APF	141.1	23	36	16
334.0	APF	70.0	63	36	8
334.0	APF	44.0	85	36	20
269.1	HI	110.0	25	51	12
269.1	HI	83.0	55	51	10
269.1	HI	92.9	51	51	12
269.1	HI	56.0	75	51	8
269.1	HI	66.0	77	51	8
269.1	HL	109.9	27	51	12
269.1	HL	186.9	11	51	22
269.1	HL	104.9	19	51	12
269.1	HL	82.9	57	51	10
269.1	HL	93.0	53	51	10
288.2	RI	175.0	23	66	20
288.2	RI	69.9	59	66	8
288.2	RI	271.1	21	66	32
288.2	RI	112.0	31	66	12
288.2	RI	42.9	87	66	20
288.2	RL	175.1	25	76	20
288.2	RL	70.1	61	76	8
288.2	RL	271.1	21	76	30

288.2	RL	112.0	31	76	12
288.2	RL	43.1	83	76	20
288.2	IR	175.0	25	56	20
288.2	IR	86.0	31	56	10
288.2	IR	70.0	55	56	8
288.2	IR	116.0	33	56	14
288.2	IR	44.0	77	56	20
288.3	LR	175.0	25	296	20
288.3	LR	86.0	29	296	10
288.3	LR	70.0	51	296	8
288.3	LR	116.0	33	296	14
288.3	LR	43.0	85	296	18

Table S 4: Validation experiments for quantitation of identified peptides in quinoa malt.

compound	prec. interday[a] [%]	prec. intraday[a] [%]	LoD[b] [nM]	LoQ[c] [nM]	calibration curve (R^2)
APF	6.72	3.91	0.7	5.6	$\boldsymbol{y = 4.67024e^{6}x - 1.45095e^{6}}$ $(\mathbf{0.99385})$
HI	7.08	3.82	1.0	3.1	$\boldsymbol{y = 1.49531e^{7}x + 4.63508e^{5}}$ $(\mathbf{0.99689})$
HL	6.89	2.18	1.8	3.9	$\boldsymbol{y = 1.21804e^{7}x + 1.48415e^{6}}$ $(\mathbf{0.99665})$
IR	1.86	2.14	1.2	111.9	$\boldsymbol{y = 7.68013e^{6}x - 4.98317e^{5}}$ $(\mathbf{0.99239})$

LR	6.19	2.53	43.3	118.1	$\boldsymbol{y = 1.53805e^5x + 21651.87}$ $(\mathbf{0.99722})$
RI	11.06	40.51	6.2	118.0	$\boldsymbol{y = 8.10880e^6x + 2.29235e^6}$ $(\mathbf{0.99689})$
RL	7.77	3.57	5.2	115.0	$\boldsymbol{y = 8.21331e^6x + 8.28024e^6}$ $(\mathbf{0.99733})$

[a]RSD = relative standard deviation. [b]LoD = limit of detection. [c]LoQ = limit of quantitation. R^2 = response factor.

Table S 5: Formulas calculated through stepwise regression analysis and used for the design of surface plots. T = temperature, t = time, m = moisture during germination. All other surface plots were made from the measured data.

**Samples**	**Formula**
Undigested quinoa malt inhibition	3.256-0.69*(t-5)+0.899*(T-11.5)-0.765*(T-11.5)*(t-5)
Undigested quinoa malt total peptide concentration	83.061-4.118*(m-48)+0.302*(t-5)+2.450*(T-11.5)+0.044*(m-48)*(m-48)-0.109*(T-11.5)*(T-11.5)-0.090*(t-5)*(T-11.5)
Digested quinoa malt total peptide concentration	130.522+6.196*(m-48)-7.472*(t-5)+3.589*(T-11.5)-7.393*(m-48)*(T-11.5)-10.256*(m-48)*(m-48)

Table S 6: Inhibitory potential of the quinoa malts when related to the original protein concentration of the extract.

**Moisture [%]**	**Temperature [°C]**	**Time [d]**	**Inhibition before digest [%]**	**Inhibition after digest [%]**
44	8	7	5.69	62.96
52	8	7	14.31	64.24
48	8	5	35.85	35.85
44	8	3	0.00	57.65
52	8	3	1.58	47.63
48	11.5	7	8.57	22.29
44	11.5	5	3.76	47.21
48	11.5	5	3.30	39.88
52	11.5	5	13.27	53.73
48	11.5	3	0.00	47.06
44	15	7	19.80	38.60
52	15	7	14.83	47.25
44	15	3	32.27	32.27
52	15	3	49.92	63.57

Table S 7: Concentrations of quantified peptides (APF, HI, HL, RI, RL, IR, and LR), in µmol/L in undigested samples (1-25, A = unmalted quinoa).

Basket	APF [µM]	HI [µM]	HL [µM]	RI [µM]	RL [µM]	IR [µM]	LR [µM]
1	0.15	0.05	0.05	0.05	0.04	0.00	1.02
2	0.14	0.08	0.1	0.06	0.07	0.00	3.08
3	0.14	0.12	0.08	0.06	0.06	0.00	2.50
4	0.19	0.13	0.15	0.05	0.00	0.00	2.37
5	0.14	0.08	0.06	0.04	0.03	0.00	1.04
6	0.00	0.01	0.02	0.00	0.00	0.00	0.00
7	0.00	0.01	0.01	0.00	0.00	0.00	0.00
8	0.00	0.01	0.00	0.00	0.00	0.00	0.00
9	0.12	0.01	0.02	0.00	0.00	0.00	0.00
10	0.14	0.04	0.03	0.00	0.00	0.00	1.20
11	0.13	0.04	0.06	0.04	0.03	0.00	1.42
12	0.14	0.03	0.03	0.00	0.00	0.00	1.18
13	0.13	0.05	0.05	0.05	0.04	0.00	2.39
14	0.17	0.11	0.11	0.00	0.00	0.00	1.72
15	0.18	0.08	0.08	0.00	0.02	0.00	3.71
16	0.12	0.05	0.07	0.04	0.03	0.00	1.18
17	0.00	0.05	0.02	0.00	0.00	0.00	0.81
18	0.00	0.04	0.03	0.00	0.00	0.00	0.72
19	0.00	0.14	0.18	0.00	0.00	0.00	1.53
20	0.16	0.09	0.11	0.00	0.00	0.00	0.71
21	0.02	0.02	0.00	0.00	0.01	0.16	0.14
22	0.00	0.02	0.03	0.00	0.00	0.00	0.32
23	0.13	0.02	0.03	0.00	0.00	0.00	0.29
24	0.15	0.14	0.15	0.05	0.05	0.00	1.15
25	0.25	0.83	0.69	0.00	0.00	0.00	1.38
A	0.00	0.00	0.00	0.00	0.00	0.00	0.00

Table S 8: Concentrations of quantified peptides (APF, HI, HL, RI, RL, IR, and LR), in µmol/L in digested samples (baskets 1-25, B = unmalted quinoa, C = enzyme control).

Basket	APF [µM]	HI [µM]	HL [µM]	RI [µM]	RL [µM]	IR [µM]	LR [µM]
1	16.88	0.80	6.60	1.23	3.21	41.46	27.15
2	15.06	0.46	3.29	1.04	1.62	28.47	31.47
3	17.13	1.18	7.74	1.64	3.93	56.11	41.05
4	20.12	1.52	8.52	0.9	2.18	51.00	33.97
5	25.85	1.44	11.26	1.27	3.86	61.43	35.15
6	25.02	0.31	6.18	0.79	2.24	51.47	41.86
7	19.34	0.24	4.46	1.38	2.39	41.3	37.23
8	19.98	0.41	6.65	0.59	3.76	65.81	39.87
9	23.26	0.43	6.10	1.46	3.54	54.03	40.94
10	21.01	0.45	3.68	1.40	1.85	57.29	34.53
11	19.37	0.53	5.03	1.51	2.14	48.97	38.57
12	23.55	0.61	4.51	1.01	1.42	47.91	38.22
13	20.42	0.46	4.89	1.44	2.02	50.97	40.06
14	18.71	0.83	4.53	0.63	0.90	93.58	38.43
15	14.54	1.24	8.33	1.63	3.87	61.9	40.54
16	18.66	0.63	7.54	1.17	3.27	54.41	47.1
17	28.16	0.59	4.74	0.49	4.38	157.9	49.91
18	22.29	0.47	3.77	0.88	1.9	69.57	35.09
19	17.62	0.61	4.53	1.14	2.87	57.18	24.87
20	19.04	0.46	2.63	0.73	1.29	59.07	27.2
21	27.23	11.28	1.09	17	2.7	38.53	54.92
22	21.40	0.50	5.20	1.37	2.88	47.87	38.09
23	33.87	0.38	4.29	0.34	1.98	170.05	77.52
24	18.87	3.88	11.91	1.79	4.37	69.31	27.83
25	20.74	0.74	4.53	1.33	1.83	55.04	45.39
B	19.85	0.25	4.48	0.53	2.01	31.3	37.58
C	4.34	0.17	0.86	0.49	1.16	16.07	13.64

Table S 9: Enzymes used for the simulated gastrointestinal digest and their protein sequences from https://www.uniprot.org.

Enzyme	Protein sequence
Chymo-<br>trypsin	>sp|P00766|CTRA_BOVIN Chymotrypsinogen A OS=Bos taurus OX=9913 PE=1 SV=1<br>CGVPAIQPVLSGLSRIVNGEEAVPGSWPWQVSLQDKTGFHFCGGSLINE<br>NWVVTAAHCGVTTSDVVVAGEFDQGSSSEKIQKLKIAKVFKNSKYNSLTI<br>NNDITLLKLSTAASFSQTVSAVCLPSASDDFAAGTTCVTTGWGLTRYTNA<br>NTPDRLQQASLPLLSNTNCKKYWGTKIKDAMICAGASGVSSCMGDSGG<br>PLVCKKNGAWTLVGIVSWGSSTCSTSTPGVYARVTALVNWVQQTLAAN
Trypsin	>sp|P00760|TRY1_BOVIN Serine protease 1 OS=Bos taurus OX=9913 GN=PRSS1 PE=1 SV=3<br>MKTFIFLALLGAAVAFPVDDDDKIVGGYTCGANTVPYQVSLNSGYHFCG<br>GSLINSQWVVSAAHCYKSGIQVRLGEDNINVVEGNEQFISASKSIVHPSY<br>NSNTLNNDIMLIKLKSAASLNSRVASISLPTSCASAGTQCLISGWGNTKSS<br>GTSYPDVLKCLKAPILSDSSCKSAYPGQITSNMFCAGYLEGGKDSCQGD<br>SGGPVVCSGKLQGIVSWGSGCAQKNKPGVYTKVCNYVSWIKQTIASN
Pepsin	>sp|P00792|PEPA_BOVIN Pepsin A OS=Bos taurus OX=9913 GN=PGA PE=1 SV=2<br>MSVVKIPLVKKKSLRQNLIENGKLKEFMRTHKYNLGSKYIREAATLVSEQ<br>PLQNYLDTEYFGTIGIGTPAQDFTVIFDTGSSNLWVPSIYCSSEACTNHN<br>RFNPQDSSTYEATSETLSIT<br>YGTGSMTGILGYDTVQVGGISDTNQIFGLSETEPGSFLYYAPFDGILGLA<br>YPSISSSGATPVFDNIWDQGLVSQDLFSVYLSSNEESGSVVIFGDIDSSY<br>YSGSLNWVPVSVEGYWQITVDSITMNGESIACSDGCQAIVDTGTSLLAG<br>PTTAISNIQSYIGASEDSSGEVVISCSSIDSLPDIVFTINGVQYPVPPSAYIL<br>QSNGICSSGFEGMDISTSSGDLWILGDVFIRQYFTVFDRGNNQIGLAPVA

Table S 10: Relative protein concentrations of the quinoa malts and the unmalted grains at different protein size groups.

**Moisture (%)**	**Duration (d)**	**Temperature (°C)**	**Relative protein concentration (ng/µl)**		
			<20 kDa	20 – 30 kDa	50 – 60 kDa
44	7	8	270.45	435.60	75.50
52	7	8	48.75	177.00	36.05
48	5	8	134.40	253.70	38.70
44	3	8	78.15	243.40	65.05
52	3	8	53.65	180.70	41.60
48	7	11.5	69.30	173.10	98.10
44	5	11.5	96.10	92.90	57.70
48	5	11.5	93.13	107.20	40.17
52	5	11.5	150.80	133.30	55.00
48	3	11.5	40.10	150.80	38.00
44	7	15	6.40	148.00	10.60
52	7	15	95.80	179.50	61.60
44	3	15	41.15	172.45	81.25
52	3	15	75.55	138.50	46.30
Unmalted quinoa			56.30	266.30	64.40

Table S 11: Correlation of the relative protein concentrations at different protein fractions and the concentration of DPP-IV inhibitory peptides. The correlation coefficient of the Pearson correlation is given as well the p-value as an indicator of significance.

**Peptide concentration**		**Relative protein concentrations (ng/µl)**		
		**<20 kDa**	**20 – 30 kDa**	**50 – 60 kDa**
APF	Correlation coefficient	0.380	-0.059	0.161
	Significance (p-value)	0.146	0.828	0.552
HI	Correlation coefficient	0.018	-0.147	-0.112
	Significance (p-value)	0.946	0.588	0.681
HL	Correlation coefficient	0.045	-0.136	-0.058
	Significance (p-value)	0.869	0.616	0.831
RI	Correlation coefficient	0.391	0.311	-0.123
	Significance (p-value)	0.134	0.241	0.650
RL	Correlation coefficient	0.674	0.352	-0.034
	Significance (p-value)	0.004	0.181	0.900
IR	Correlation coefficient	0.163	-0.267	-0.368
	Significance (p-value)	0.547	0.318	0.161
LR	Correlation coefficient	0.450	-0.047	0.023
	Significance (p-value)	0.081	0.862	0.932
Inhibition	Correlation coefficient	-0.145	-0.221	-0.107
	Significance (p-value)	0.593	0.410	0.694

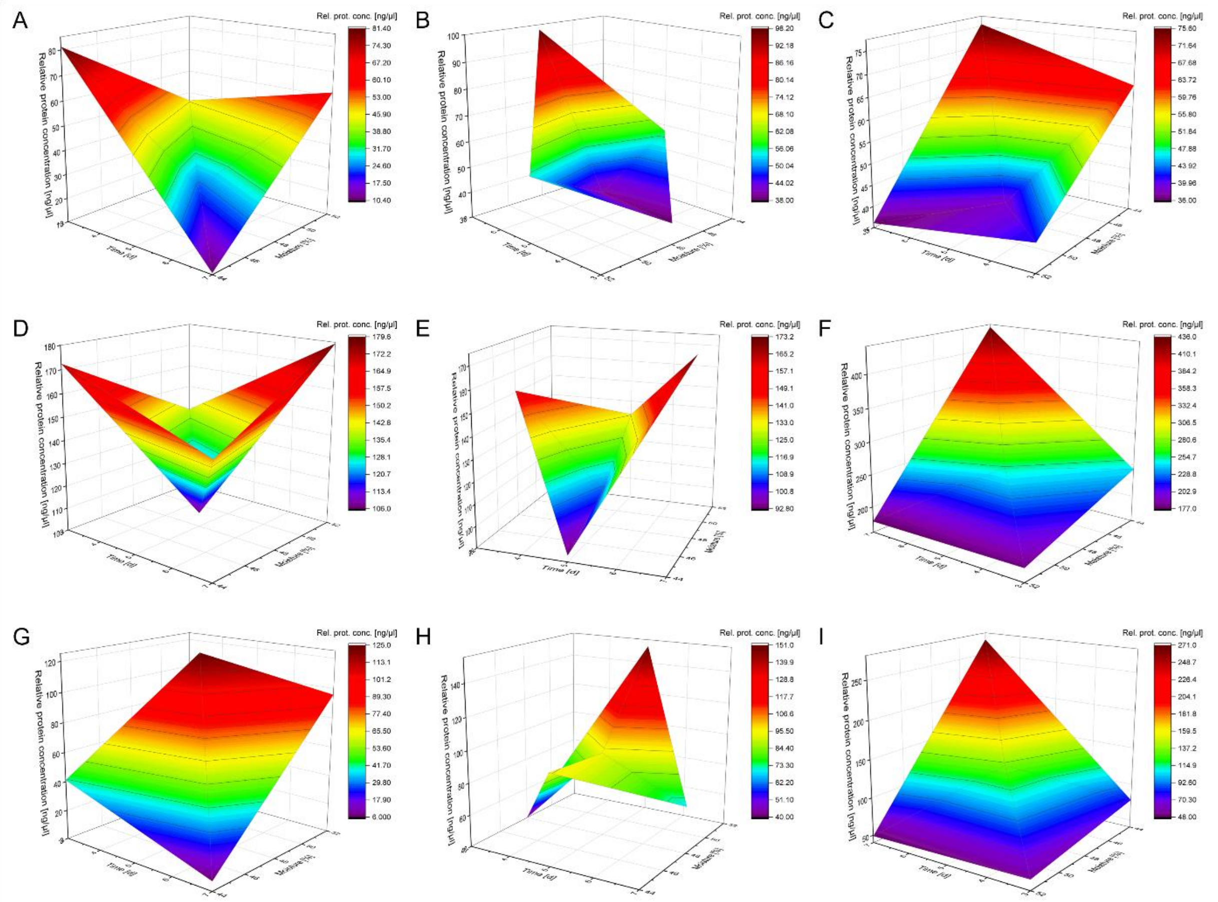

Figure S 1: Surface plot of the measured relative protein concentrations of malts germinated at different moistures over time. The plot shows the data from the protein concentrations of the fraction below 20 kDa from malts germinated at 8 °C (A), 11.5 °C (B), or 15 °C (C), the protein size fraction 20 – 30 kDa from malts germinated at 8 °C (D), 11.5 °C (E), or 15 °C (F), and the protein size fraction 50 – 60 kDa from malts germinated at 8 °C (G), 11.5 °C (H), or 15 °C (I).

References

(1) Gallego, M.; Aristoy, M.-C.; Toldrá, F. Dipeptidyl peptidase IV inhibitory peptides generated in spanish dry-cured ham. *Meat science* **2014**, *96* (2 Pt A), 757–761. DOI: 10.1016/j.meatsci.2013.09.014.

(2) Nongonierma, A. B.; FitzGerald, R. J. Dipeptidyl peptidase IV inhibitory and antioxidative properties of milk protein-derived dipeptides and hydrolysates. *Peptides* **2013**, *39*, 157–163. DOI: 10.1016/j.peptides.2012.11.016.

(3) Nongonierma, A. B.; Dellafiora, L.; Paolella, S.; Galaverna, G.; Cozzini, P.; FitzGerald, R. J. In silico approaches applied to the study of peptide analogs of Ile-Pro-Ile in relation to their dipeptidyl peptidase IV inhibitory properties. *Frontiers in endocrinology* **2018**, *9*, 329. DOI: 10.3389/fendo.2018.00329.

(4) Lammi, C.; Zanoni, C.; Arnoldi, A.; Vistoli, G. Peptides derived from soy and lupin protein as dipeptidyl-peptidase IV inhibitors: In vitro biochemical screening and in silico molecular modeling study. *Journal of Agricultural and Food Chemistry* **2016**, *64* (51), 9601–9606. DOI: 10.1021/acs.jafc.6b04041.

(5) Huang, S.-L.; Jao, C.-L.; Ho, K.-P.; Hsu, K.-C. Dipeptidyl-peptidase IV inhibitory activity of peptides derived from tuna cooking juice hydrolysates. *Peptides* **2012**, *35* (1), 114–121. DOI: 10.1016/j.peptides.2012.03.006.

(6) Lin, Y.-S.; Han, C.-H.; Lin, S.-Y.; Hou, W.-C. Synthesized Peptides from Yam Dioscorin Hydrolysis in Silico Exhibit Dipeptidyl Peptidase-IV Inhibitory Activities and Oral Glucose Tolerance Improvements in Normal Mice. *Journal of*

Agricultural and Food Chemistry **2016**, *64* (33), 6451-6458. DOI: 10.1021/acs.jafc.6b02403.

(7) Lacroix, I. M. E.; Li-Chan, E. C. Y. Evaluation of the potential of dietary proteins as precursors of dipeptidyl peptidase (DPP)-IV inhibitors by an in silico approach. *Journal of Functional Foods* **2012**, *4* (2), 403–422. DOI: 10.1016/j.jff.2012.01.008.

(8) Sila, A.; Alvarez, O. M.; Haddar, A.; Frikha, F.; Dhulster, P.; Nedjar-Arroume, N.; Bougatef, A. Purification, identification and structural modelling of DPP-IV inhibiting peptides from barbel protein hydrolysate. *Journal of chromatography. B, Analytical technologies in the biomedical and life sciences* **2016**, *1008*, 260–269. DOI: 10.1016/j.jchromb.2015.11.054.

(9) Neves, A. C.; Harnedy, P. A.; O'Keeffe, M. B.; Alashi, M. A.; Aluko, R. E.; FitzGerald, R. J. Peptide identification in a salmon gelatin hydrolysate with antihypertensive, dipeptidyl peptidase IV inhibitory and antioxidant activities. *Food Research International* **2017**, *100*, 112-120. DOI: 10.1016/j.foodres.2017.06.065.

(10) Kuo-Chiang, H.; Yu-Shan, T.; Shih-Li, H.; Chia-Ling, J. Dipeptidyl peptidase-IV inhibitory activity of peptides in porcine skin gelatin hydrolysates. In *Bioactive Food Peptides in Health and Disease*, Blanca, H.-L., Chia-Chien, H. Eds.; IntechOpen, 2013; p Ch. 8.

(11) *BIOPEP-UWM database*. Chair of Food Biochemistry at the Faculty of Food Science, University of Warmia and Mazury in Olsztyn, https://biochemia.uwm.edu.pl/en/start/ (accessed 19.12.2023).

(12) Nongonierma, A. B.; FitzGerald, R. J. Structure activity relationship modelling of milk protein-derived peptides with dipeptidyl peptidase IV (DPP-IV) inhibitory activity. *Peptides* **2016**, *79*, 1–7. DOI: 10.1016/j.peptides.2016.03.005.

(13) Tulipano, G.; Sibilia, V.; Caroli, A. M.; Cocchi, D. Whey proteins as source of dipeptidyl dipeptidase IV (dipeptidyl peptidase-4) inhibitors. *Peptides* **2011**, *32* (4), 835-838. DOI: 10.1016/j.peptides.2011.01.002.

(14) Silveira, S. T.; Martínez-Maqueda, D.; Recio, I.; Hernández-Ledesma, B. Dipeptidyl peptidase-IV inhibitory peptides generated by tryptic hydrolysis of a whey protein concentrate rich in β-lactoglobulin. *Food chemistry* **2013**, *141* (2), 1072–1077. DOI: 10.1016/j.foodchem.2013.03.056.

(15) Nongonierma, A. B.; FitzGerald, R. J. An in silico model to predict the potential of dietary proteins as sources of dipeptidyl peptidase IV (DPP-IV) inhibitory peptides. *Food chemistry* **2014**, *165*, 489–498. DOI: 10.1016/j.foodchem.2014.05.090.

(16) Xu, F.; Yao, Y.; Xu, X.; Wang, M.; Pan, M.; Ji, S.; Wu, J.; Jiang, D.; Ju, X.; Wang, L. Identification and quantification of DPP-IV-inhibitory peptides from hydrolyzed-rapeseed-protein-derived napin with analysis of the interactions

between key residues and protein domains. *Journal of Agricultural and Food Chemistry* **2019**, *67* (13), 3679–3690. DOI: 10.1021/acs.jafc.9b01069.

(17) Araki, M.; Kanegawa, N.; Iwata, H.; Sagae, Y.; Ito, K.; Masuda, K.; Okuno, Y. Hydrophobic interactions at subsite s1' of human dipeptidyl peptidase IV contribute significantly to the inhibitory effect of tripeptides. *Heliyon* **2020**, *6* (6), e04227. DOI: 10.1016/j.heliyon.2020.e04227.

(18) Lacroix, I. M. E.; Li-Chan, E. C. Y. Isolation and characterization of peptides with dipeptidyl peptidase-IV inhibitory activity from pepsin-treated bovine whey proteins. *Peptides* **2014**, *54*, 39-48. DOI: 10.1016/j.peptides.2014.01.002.

(19) Ji, W.; Zhang, C.; Ji, H. Purification, identification and molecular mechanism of two dipeptidyl peptidase IV (DPP-IV) inhibitory peptides from antarctic krill (euphausia superba) protein hydrolysate. *Journal of chromatography. B, Analytical technologies in the biomedical and life sciences* **2017**, *1064*, 56–61. DOI: 10.1016/j.jchromb.2017.09.001.

(20) Liu, R.; Zhou, L.; Zhang, Y.; Sheng, N.-J.; Wang, Z.-K.; Wu, T.-Z.; Wang, X.-Z.; Wu, H. Rapid Identification of Dipeptidyl Peptidase-IV (DPP-IV) Inhibitory Peptides from Ruditapes philippinarum Hydrolysate. In *Molecules*, 2017; Vol. 22.

(21) Uenishi, H.; Kabuki, T.; Seto, Y.; Serizawa, A.; Nakajima, H. Isolation and identification of casein-derived dipeptidyl-peptidase 4 (DPP-4)-inhibitory peptide LPQNIPPL from gouda-type cheese and its effect on plasma glucose in

rats. *International Dairy Journal* **2012**, *22* (1), 24-30. DOI: 10.1016/j.idairyj.2011.08.002.

(22) Taga, Y.; Hayashida, O.; Kusubata, M.; Ogawa-Goto, K.; Hattori, S. Production of a novel wheat gluten hydrolysate containing dipeptidyl peptidase-IV inhibitory tripeptides using ginger protease. *Bioscience, Biotechnology, and Biochemistry* **2017**, *81* (9), 1823-1828. DOI: 10.1080/09168451.2017.1345615.

(23) Wang, F.; Zhang, Y.; Yu, T.; He, J.; Cui, J.; Wang, J.; Cheng, X.; Fan, J. Oat globulin peptides regulate antidiabetic drug targets and glucose transporters in Caco-2 cells. *Journal of Functional Foods* **2018**, *42*, 12–20. DOI: 10.1016/j.jff.2017.12.061.

(24) Lammi, C.; Bollati, C.; Ferruzza, S.; Ranaldi, G.; Sambuy, Y.; Arnoldi, A. Soybean- and Lupin-Derived Peptides Inhibit DPP-IV Activity on In Situ Human Intestinal Caco-2 Cells and Ex Vivo Human Serum. In *Nutrients*, 2018; Vol. 10.

(25) Hatanaka, T.; Inoue, Y.; Arima, J.; Kumagai, Y.; Usuki, H.; Kawakami, K.; Kimura, M.; Mukaihara, T. Production of dipeptidyl peptidase IV inhibitory peptides from defatted rice bran. *Food chemistry* **2012**, *134* (2), 797–802. DOI: 10.1016/j.foodchem.2012.02.183.

(26) Song, J. J.; Wang, Q.; Du, M.; Ji, X. M.; Mao, X. Y. Identification of dipeptidyl peptidase-IV inhibitory peptides from mare whey protein hydrolysates. *Journal of dairy science* **2017**, *100* (9), 6885–6894. DOI: 10.3168/jds.2016-11828.

(27) Uchida, M.; Ohshiba, Y.; Mogami, O. Novel dipeptidyl peptidase-4–inhibiting peptide derived from β-lactoglobulin. *Journal of Pharmacological Sciences* 2011, *117* (1), 63-66. DOI: 10.1254/jphs.11089SC.

(28) Harnedy-Rothwell, P. A.; McLaughlin, C. M.; O'Keeffe, M. B.; Le Gouic, A. V.; Allsopp, P. J.; McSorley, E. M.; Sharkey, S.; Whooley, J.; McGovern, B.; O'Harte, F. P. M.; et al. Identification and characterisation of peptides from a boarfish (capros aper) protein hydrolysate displaying in vitro dipeptidyl peptidase-IV (DPP-IV) inhibitory and insulinotropic activity. *Food research international (Ottawa, Ont.)* 2020, *131*, 108989. DOI: 10.1016/j.foodres.2020.108989.
